# Cardioprotective Effect of Stem-Leaf Saponins From *Panax notoginseng* on Mice With Sleep Deprivation by Inhibiting Abnormal Autophagy Through PI3K/Akt/mTOR Pathway

**DOI:** 10.3389/fcvm.2021.694219

**Published:** 2021-09-16

**Authors:** Yin Cao, Qinglin Li, Yingbo Yang, Zunji Ke, Shengqi Chen, Mingrui Li, Wenjing Fan, Hui Wu, Jinfeng Yuan, Zhengtao Wang, Xiaojun Wu

**Affiliations:** ^1^Shanghai Key Laboratory of Compound Chinese Medicines, The Ministry of Education (MOE) Key Laboratory for Standardization of Chinese Medicines, The State Administration of TCM (SATCM) Key Laboratory for New Resources and Quality Evaluation of Chinese Medicine, Institute of Chinese Materia Medica, Shanghai University of Traditional Chinese Medicine, Shanghai, China; ^2^Key Laboratory of Xin'an Medicine, Ministry of Education, Anhui Key Laboratory of R&D of Chinese Medicine, Anhui University of Chinese Medicine, Hefei, China; ^3^Kanion Pharmaceutical Co., Ltd, Lianyungang, China; ^4^Academy of Integrative Medicine, Shanghai University of Traditional Chinese medicine, Shanghai, China

**Keywords:** stem-leaf saponin from *Panax notoginseng*, autophagy, sleep deprivation, cardioprotection, apoptosis

## Abstract

Sleep deprivation (SD) may lead to serious myocardial injury in cardiovascular diseases. Saponins extracted from the roots of *Panax notoginseng*, a traditional Chinese medicine beneficial to blood circulation and hemostasis, are the main bioactive components exerting cardiovascular protection in the treatment of heart disorders, such as arrhythmia, ischemia and reperfusion injury, and cardiac hypertrophy. This study aimed to explore the protective effect of stem-leaf saponins from *Panax notoginseng* (SLSP) on myocardial injury in SD mice. SD was induced by a modified multi-platform method. Cardiac morphological changes were assessed by hematoxylin and eosin (H&E) staining. Heart rate and ejection fraction were detected by specific instruments. Serum levels of atrial natriuretic peptide (ANP) and lactate dehydrogenase (LDH) were measured with biochemical kits. Transmission electron microscopy (TEM), immunofluorescent, and Western blotting analysis were used to observe the process and pathway of autophagy and apoptosis in heart tissue of SD mice. *In vitro*, rat H9c2 cells pretreated with rapamycin and the effect of SLSP were explored by acridine orange staining, transient transfection, flow cytometry, and Western blotting analysis. SLSP prevented myocardial injury, such as morphological damage, accumulation of autophagosomes in heart tissue, abnormal high heart rate, serum ANP, and serum LDH induced by SD. In addition, it reversed the expressions of proteins involved in the autophagy and apoptosis and activated PI3K/Akt/mTOR signaling pathway that is disturbed by SD. On H9c2 cells induced by rapamycin, SLSP could markedly resume the abnormal autophagy and apoptosis. Collectively, SLSP attenuated excessive autophagy and apoptosis in myocardial cells in heart tissue induced by SD, which might be acted through activating PI3K/Akt/mTOR signaling pathway.

## Introduction

Sleep deprivation (SD) caused by undesirable lifestyle habits and sleep disorders, such as insomnia, obstructive sleep apnea, and neurological diseases, has become a worldwide health problem (1). It can increase catecholamine, coronary vasomotor tone, blood pressure, and heart rate, thereby disturbs the balance of oxygen supply and demand, resulting in excessive vasoconstriction, fibrosis, and cardiac remodeling in cardiac diseases (2). More and more evidences indicate the causality of SD with the increased morbidity and mortality of cardiovascular diseases (3). Blood biomarkers indicating myocardial injury, such as atrial natriuretic peptide (ANP), B-type natriuretic peptide (BNP), creatine phosphokinase (CK-MB), lactate dehydrogenase (LDH), and alkaline phosphatase (ALP) have been exposed to be abnormally changed in SD relevant cardiac diseases (4–7).

Autophagy is a cellular process for bulk degradation and recycling of cytoplasmic components, such as proteins and organelles, which involves sequestration of cytosolic constituents in autophagosomes and degradation in lysosomes (8). Basal or constitutional autophagy is important or even essential for the maintenance of cellular homeostasis (9). Dysregulated autophagy has been discovered in many cardiac diseases, such as dilated cardiomyopathy (10–12), valvular disease (13), and ischemic heart disease (14–16). Many reports revealed the close link or causality between the dysregulated autophagy and apoptosis in myocardial injury (17). Interestingly, drugs either activating or deactivating autophagy have both been demonstrated to alleviate myocardial injury (17), suggesting the beneficial effects of the recovery of aberrant autophagy on cardiac diseases.

Saponins extracted from the roots of *Panax notoginseng*, a traditional Chinese medicine beneficial to blood circulation and hemostasis, are the main bioactive components exerting cardiovascular protection in the treatment of heart disorders, such as arrhythmia, ischemia and reperfusion injury, and cardiac hypertrophy. The saponins from the flower of *P. notoginseng* were shown to ameliorate acute myocardial infarction in rats (18). Our previous study found that the stem-leaf saponins from *P. notoginseng* (SLSP) protect the hippocampal neurons, and improve the impaired memory in mice induced by SD through regulating autophagy and apoptosis (19). However, whether SLSP can also modulate the aberrant autophagy of injured myocardium in SD-induced mice has not been clarified. In the present study, the protective effects of SLSP on myocardium in SD-induced mice and the possible underlying mechanism were explored from the aspect of inhibiting aberrant autophagy and apoptosis. The current study findings may provide a novel treatment regimen for the prevention of cardiac injury induced by SD in the clinic.

### Reagent

**Figure 1:**
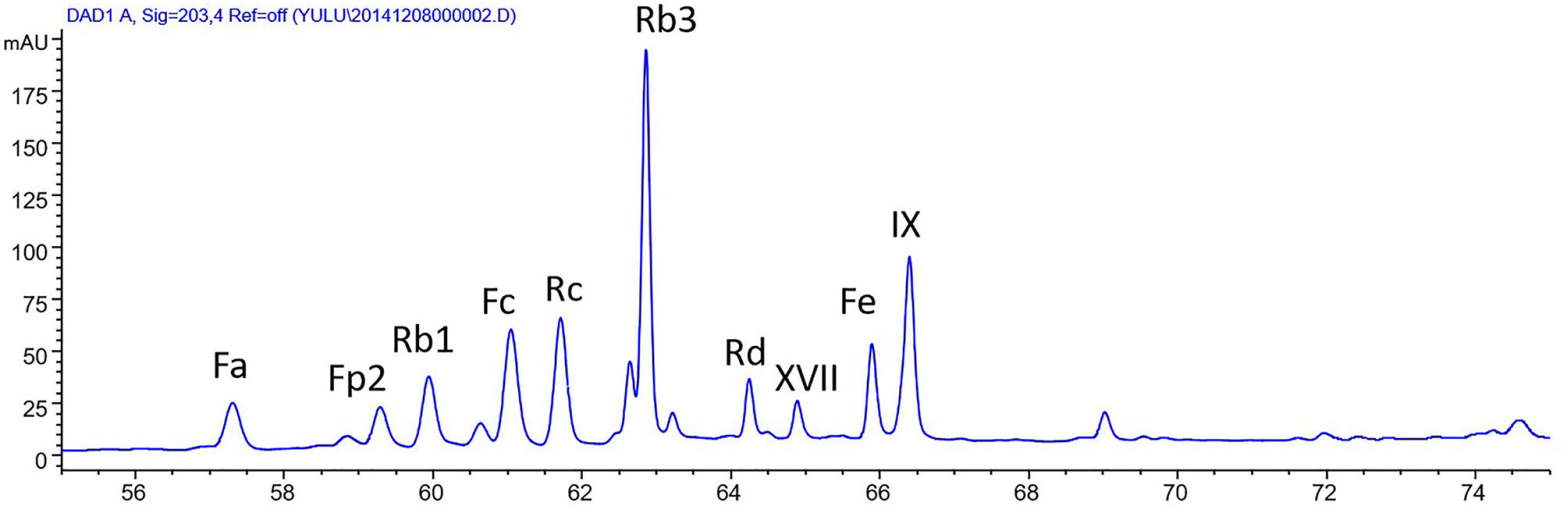
High-performance liquid chromatography (HPLC) analysis of stem-leaf saponins from *Panax notoginseng* (SLSP).

The stem-leaf saponins from *P. notoginseng* (>95% purity) was bought from Qidan Limited Liability Company (Wenshan, China). Saponins in SLSP contain as several ginsenosides and notoginsenosides as following: Rb3 (17.4%), notoginsenoside Fc (11.8%), ginsenoside Rc (11.1%), notoginsenoside IX (7.72%), notoginsenoside FP2 (5.59%), notoginsenoside Fe (5.45%), ginsenoside Rb1 (4.86%), notoginsenoside Fa (4.13%), and ginsenoside Rd (3.04%). High-performance liquid chromatography (HPLC) analysis of SLSP was shown in Figure 1. The antibodies against Akt (9272), Bax (2772), Bcl-2 (2870), Beclin-1 (3495), LC3B (3868), phospho-Akt (9271), phospho-inositide 3- kinase (PI3K) p85 (4228), and PI3K p85 (4257) were obtained from Cell Signaling Technology (Danvers, MA, USA). The antibodies against p62 (ab109012), p-mTOR (ab84400), mTOR (ab87540), and β-actin (ab8227) were purchased from Abcam (Cambridge, MA, USA). Rapamycin and 3-methyladenine (3-MA) were provided by Selleckchem (Boston, MA, USA). The kits for the measurements of serum LDH and ANP were obtained from Nanjing Jiancheng Bio-company (Nanjing, China).

### Animals and Treatment

In this study, 50 male C57BL/6 mice, aged 5 weeks and weighing 18–22 g, were randomly divided into normal group, SD group, and SLSP groups (25, 50, and 100 mg/kg). After adaptive feeding for a week, the mice in each group were given the drug by gavage for 9 days continuously. Except for the normal group, the mice in each group were subjected to modified multi-platform sleep deprivation for 48 h on the 8th and 9th day according to a modified multiple platform method (20). During SD, they could eat and drink freely. The mice were offered by the animal Research Center of Shanghai University of Traditional Chinese Medicine and provided a standard feeding environment (23 ± 2°C, light/dark cycle for 12 h). The heart rate (HR) was recorded and analyzed with Acknowledge 4.1 (BIOPAC Systems Inc., CA, USA). Vevo 2100 187 system equipped with a 30-MHz MS400 transducer was used to detect the ejection fraction. After administrated with an overdose of 2% pentobarbital sodium, the mice were sacrificed. The serum was collected to measure LDH and ANP with respective kits.

### Histopathological Analysis and Immunohistochemistry

The heart tissues were dissected and fixed in 4% of paraformaldehyde solution for more than 24 h. For hematoxylin and eosin (H&E) staining, the sections were stained by hematoxylin and eosin according to the process described previously (21).

For immunohistochemistry, the heart tissues were washed with phosphoric acid buffer saline (PBS), soaked in 10 and 30% sucrose solution, respectively, for more than 24 h. Afterward, the heart was embedded, frozen, and cut into slices with a thickness of 20 μm. After the infiltration and blocking for 30 min, the sections were incubated with a primary antibody against LC3B overnight at 4°C, followed by the incubation with a secondary antibody combined with Alexa Fluor 488. Fluorescent images were taken using the Olympus VS120 virtual Slide scanner (Olympus Corporation, Tokyo, Japan).

### Transmission Electron Microscopy (TEM)

Following perfusion with PBS and fixation with 2% glutaraldehyde, the heart tissues of mice were sectioned and cut into ultra-thin sections. After dehydration, the sections were stained with uranyl acetate and lead citrate. The ultrastructure images were taken under HT-7700 transmission electron microscope (Hitachi, Tokyo, Japan).

### Cell Culture and Treatment

Rat H9c2 cell line was obtained from Cell Bank of Type Culture Collection of the Chinese Academy of Sciences. The cells were maintained in DMEM basic medium, supplied with 10% fetal bovine serum and 1% penicillin/streptomycin. Prior to the drug treatment, the cells were seeded at a density of 5 × 10^5^ cells/ml in cell culture flasks or 24-well-plates and cultured overnight. After pretreated with rapamycin (50 μg/ml) for 4 h, the cells were incubated with complete medium, 3-MA (2.5 mmol/L) or SLSP at doses of 12.5, 25, and 50 μg/ml for 48 h. Then, the cells were lysed in lysis buffer with protease and phosphatase inhibitors for further Western blotting analysis.

### Transient Transfection

The H9c2 cells were seeded at a density of 4 × 10^5^ cells/ml in 24-well-plates. When growing to 70–80% confluence, the cells were transiently transfected with GFP-LC3 or tf-LC3 plasmids (0.5 μg/well) using Lipofectamine 2000 in accordance with the manual of the manufacturer. After 6 h, the opti-medium was removed and replaced with rapamycin (50 μg/ml) for 4 h. Then, rapamycin was removed and incubated with 3-MA (2.5 mM) or SLSP (12.5, 25, and 50 μg/ml) for 48 h. The fluorescent images were taken by an inverted fluorescence microscope.

### Acridine Orange Staining

The H9c2 cells were induced with rapamycin and treated with 3-MA or SLSP as mentioned earlier. Ethidium bromide (EB) solution and acridine orange (AO) solution were mixed in equal quantity as a working solution and 80 μl of the working solution was added to 4 ml PBS. The medium was discarded and the cells were rinsed with PBS. The diluted working fluid of 200 μl was added to each well. After half an hour, the images were observed and photographed under a fluorescence microscope after 2–5 min at room temperature. Generally, the living cells are uniformly stained in green, while autophagic cells have an orange cytoplasm.

### Flow Cytometry

The H9c2 cells were induced with rapamycin and treated with 3-MA or SLSP as described previously. Then, the cells were collected with trypsin without ethylenediaminetetraacetic acid (EDTA). After washing two times with PBS, the cells were incubated with propidium iodide (PI, 2.5 μg/ml) and annexin V (2 μg/ml) for 10 min. Flow cytometry was carried out on a flow cytometer (Guava easyCyte HT, Millipore, Germany).

### Western Blotting Analysis

Twenty microgram proteins from each sample were separated on 12% gel and transferred onto polyvinylidene difluoride (PVDF) membranes. After blocking with non-fat milk solution (5%) for 1 h, the membranes were incubated with respective primary antibodies overnight at 4°C. Thereafter, they were washed with PBST and incubated with secondary antibodies for 1 h at room temperature. The protein bands were observed by ECL Prime Kit and quantified with ImageJ 1.46r software.

### Statistical Analysis

All data are presented as mean ± SEM. One-way ANOVA with Dunnett's *post-hoc* analysis was performed to analyze the differences among the groups using IBM SPSS Statistics 21 (SPSS Inc., IL, USA).

## Results

### SLSP Reduced Cardiac Injury Induced by SD in Mice

**Figure 2:**
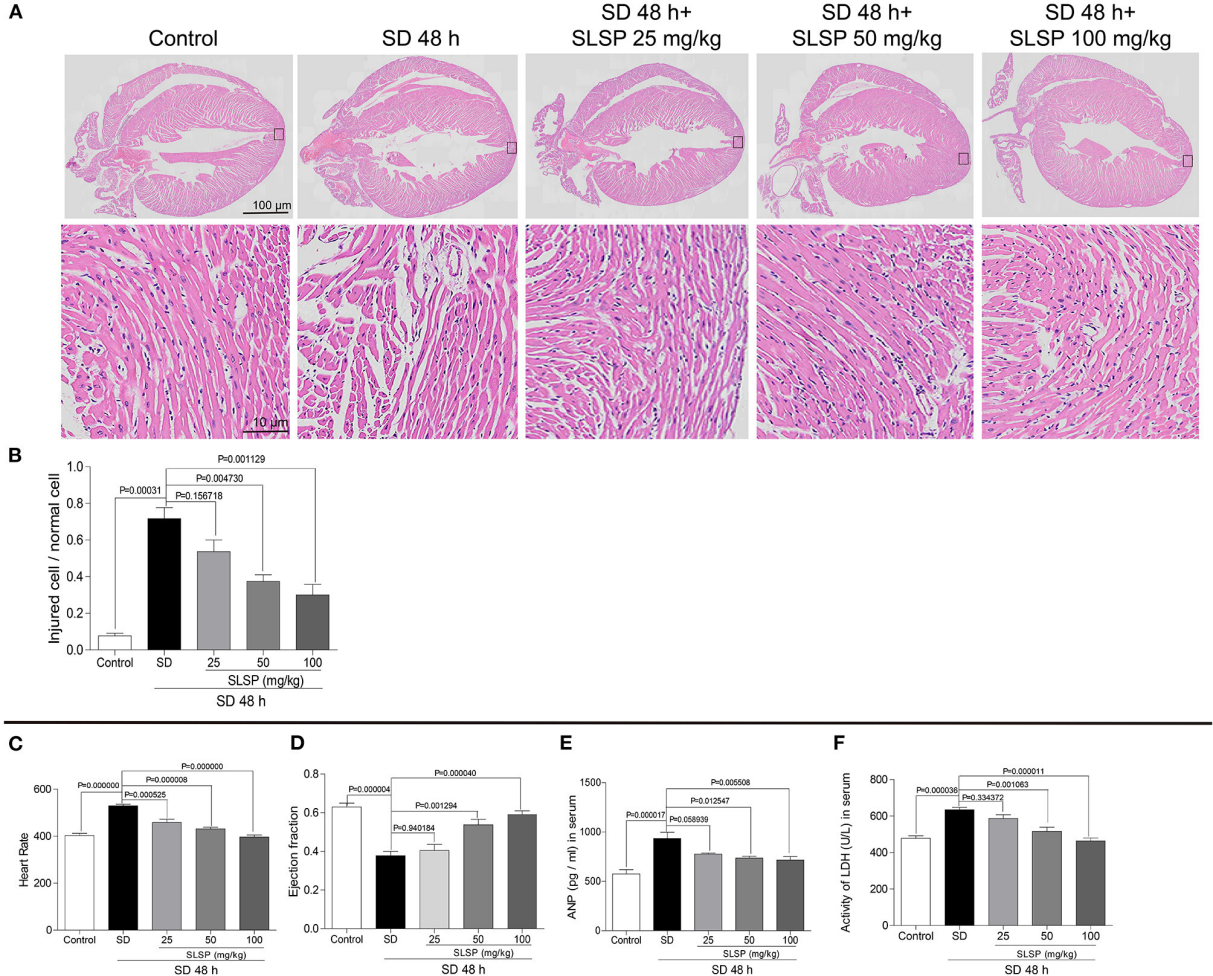
SLSP inhibited abnormal changes in cardiac tissue morphology **(A,B)**, heart rate **(C)**, ejection fraction **(D)**, serum ANP **(E)**, and serum LDH **(F)** in SD mice. *N* = 3/group.

As shown in Figure 2A, the myocardial cells in the normal group mice were closely aligned, with clear transverse striae and less extracellular stroma. However, SD changed the morphological structure of myocardial cells, resulting in myocardial fiber disorder, enlarged intercellular space, blurred transverse striae, disordered cell arrangement, and evacuation. SLSP treatment prevented the abnormal change of myocardial cells. For instance, myocardial cells in the SLSP treated group mice were slightly disordered, with intermittent enlargement and blurred transverse striae. In Figure 2B, SLSP used at 50 and 100 mg/kg decreased the ratio of damaged cells significantly compared with the SD group (*P* = 0.004730 and *P* = 0.001129). Moreover, SD increased the heart rate of mice significantly compared with the control group (as shown in Figure 2C, *P* = 0.000000), as well as the serum LDH and ANP levels (Figures 2E,F, *P* = 0.00017 and *P* = 0.000036). Additionally (Figure 2D), SLSP used at 50 and 100 mg/kg significantly increased the declined cardiac ejection fraction induced by SD (*P* = 0.001294 and *P* = 0.000040). These results clearly demonstrated that SLSP could rescue the myocardial injury caused by SD.

### SLSP Inhibited Excessive Autophagy and Apoptosis in Heart Tissue of Mice Induced by SD

**Figure 3:**
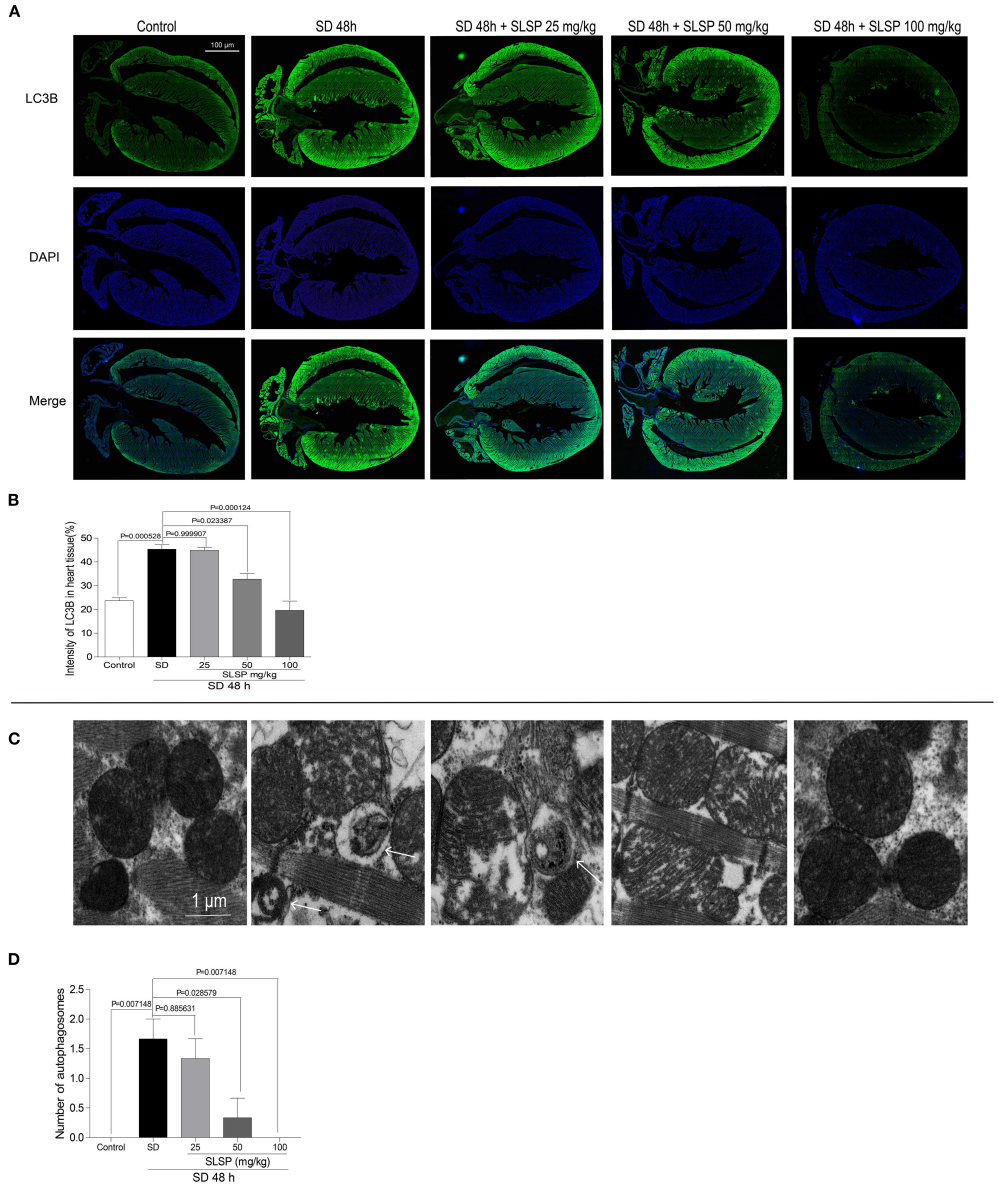
SLSP suppressed the formation of autophagosomes and the increase of LC3B in the heart tissue of SD mice. **(A)** Expression of LC3B in heart tissue of mice by IHC. **(B)** Intensity of LC3B in heart tissue. **(C)** Transmission electron microscopy (TEM) images of heart tissue. **(D)** Number of autophagosomes exposed by TEM in heart tissue. *N* = 3/group.

**Figure 4:**
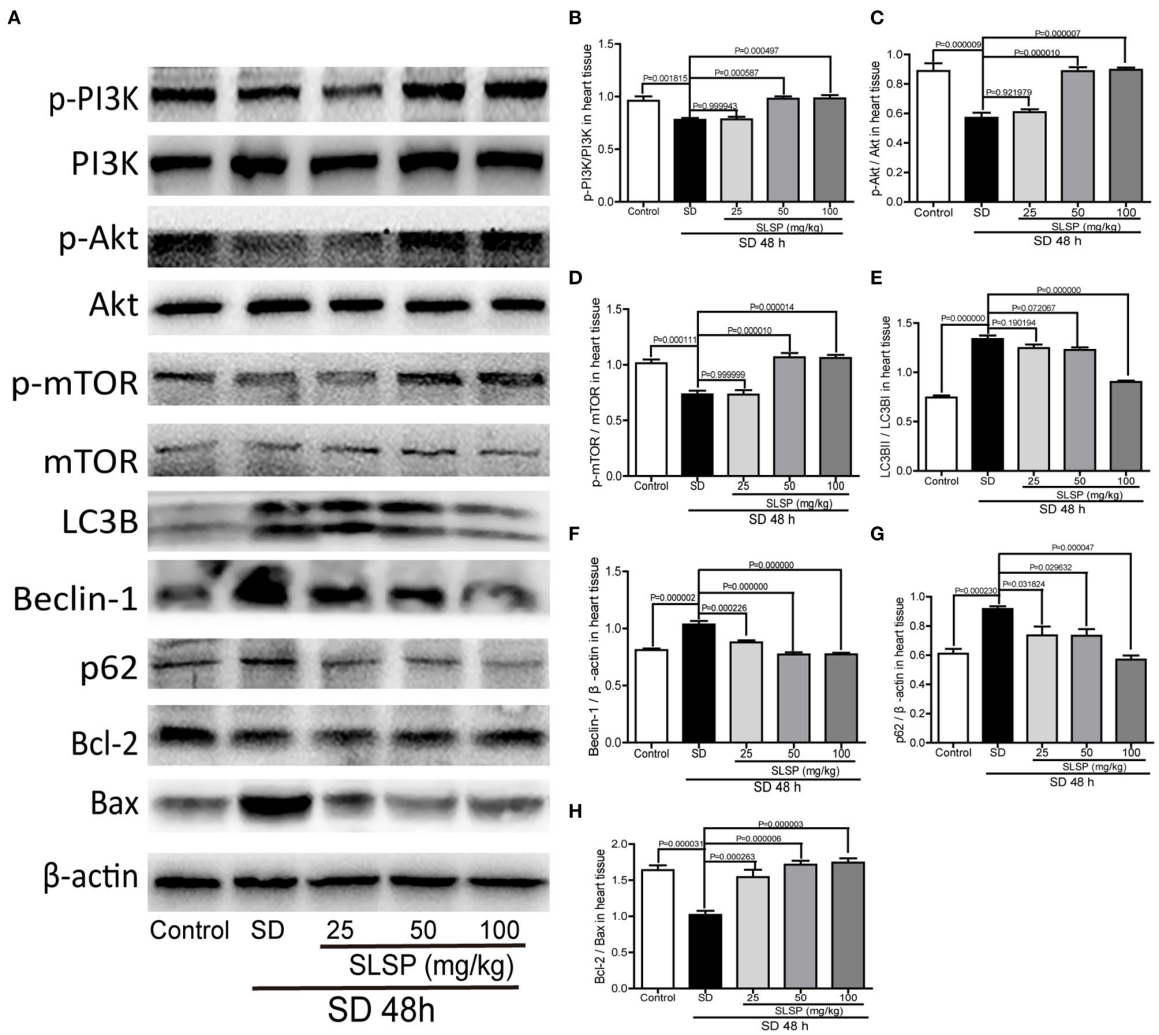
SLSP inhibited the expressions of proteins related to autophagy and apoptosis in heart tissue of SD mice. **(A)** Western blotting bands of respective proteins in heart tissue of mice. **(B–H)** Gray intensity analysis. *N* = 5/group.

Consistently, as exposed in Figures 3A,B, LC3B immunostaining revealed that SLSP used at 50 and 100 mg/kg decreased the intensity of LC3B in heart tissue of sleep-deprived mice significantly (*P* = 0.023387 and *P* = 0.000124). Meanwhile, compared with the SD mice, the number of autophagosomes in SLSP treated mice were decreased (Figures 3C,D). SD decreased the phosphorylation of PI3K, Akt and mTOR (Figures 4A–D) while increased the expressions of Beclin-1, LC3B, and p62 in mice compared with the control group (Figures 4A,E–G). SD also changed the expression of apoptotic protein Bcl-2 and Bax, therefore, the ratio of Bcl-2 to Bax was reduced remarkably (Figures 4A,H *P* = 0.002222). However, SLSP, especially administered at 100 mg/kg, inhibited the effects of SD on the expressions of proteins involved in apoptosis and autophagy in mice (Figure 4). These results suggested that SLSP could protect the heart tissue from excessive autophagy and apoptosis.

### SLSP Attenuated Excessive Autophagy and Apoptosis in H9c2 Cells

**Figure 5:**
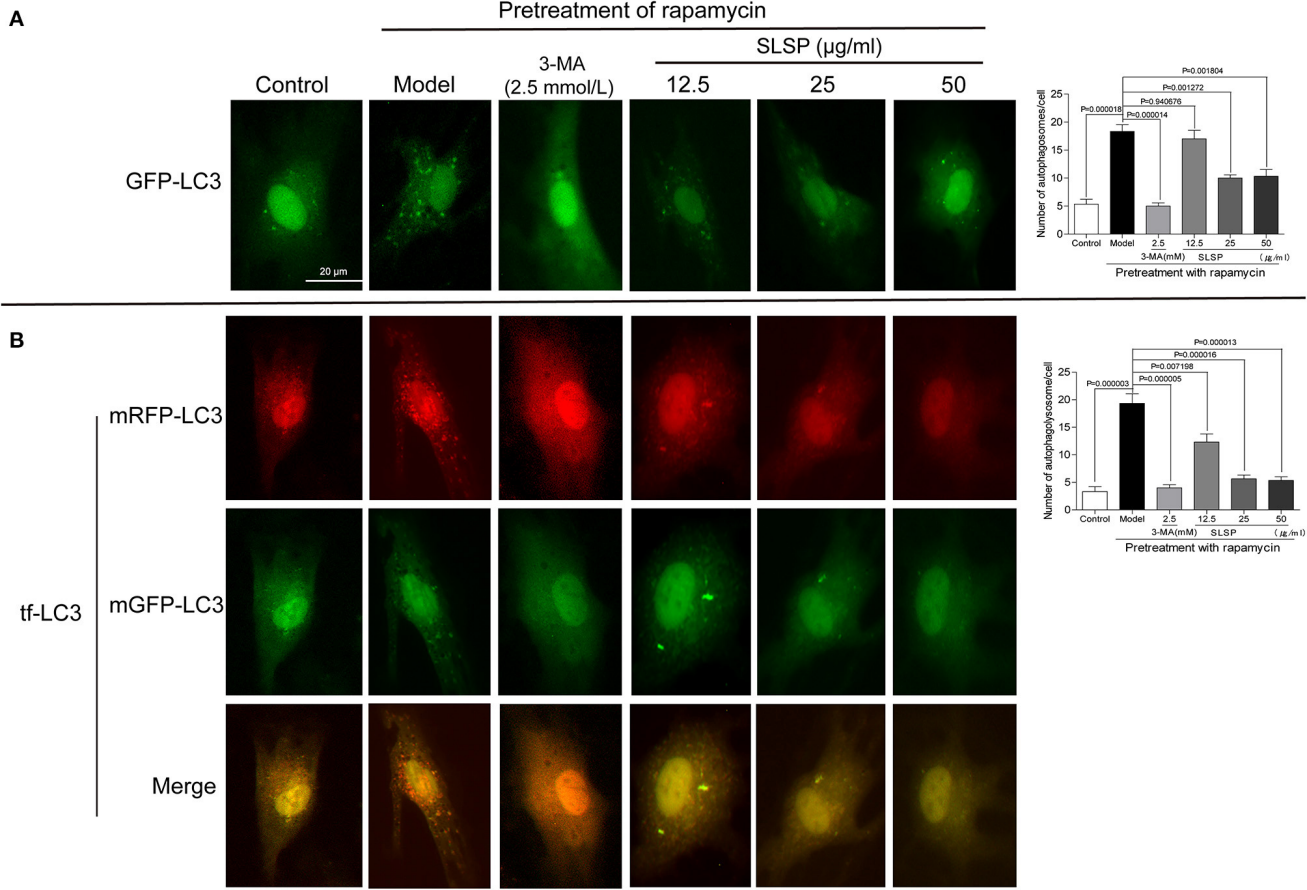
SLSP reduced autophagosomes and autolysosomes in H9c2 cells transfected with GFP-LC3 and tf-LC3. **(A)** SLSP decreased autophagosomes in H9c2 cells transfected with GFP-LC3. **(B)** SLSP mitigated autophagosomes and autolysosomes in H9c2 cells transfected with tf-LC3. Scale bar: 10 μm. *N* = 3/group.

**Figure 6:**
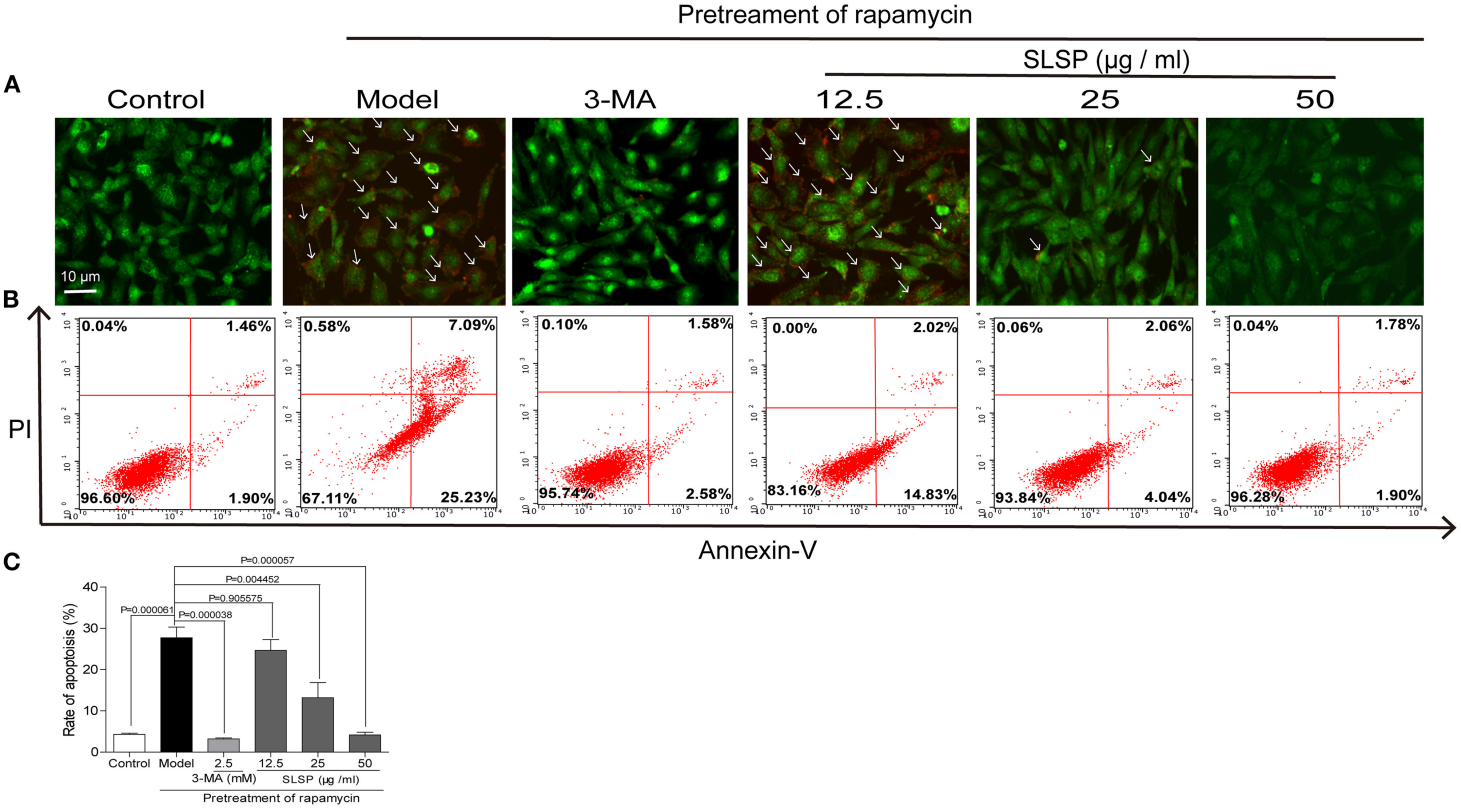
SLSP inhibited the autophagy and early apoptosis of H9c2 cells. **(A)** SLSP reduced acidic vesicular organelles in H9c2 cells pretreated with rapamycin according to acridine orange (AO) staining. Arrows were added to mark the cells with acidic vesicular organelles. **(B)** SLSP decreased the percentage of apoptotic H9c2 cells pretreated with rapamycin through AnnexinV/PI staining. H9c2 cells were incubated with different concentrations (12.5, 25, and 50 μg/ml) of SLSP for 48 h after being pretreated with rapamycin (50 μg/ml) for 4 h and then subjected to annexin V and propidium iodide (PI) staining. **(C)** Rate of apoptosis. *N* = 3/group.

As shown in Figure 5A, in H9c2 cells transfected with a green fluorescent protein (GFP)-LC3 plasmid, rapamycin induced prominent autophagosome formation indicated by increased GFP puncta in the cytoplasm. SLSP, as well as 3-MA, significantly decreased the number of GFP puncta. Moreover, in H9c2 cells transfected with tandem fluorescent-tagged LC3 reporter (tf-LC3), SLSP not only reduced the formation of autophagosomes but also decreased the formation of autolysosomes, which were indicated by GFP and RFP puncta, respectively (Figure 5B). Similarly, as shown in Figure 6A, SLSP treatment remarkably reduced the number of acidic vesicular organelles stained in orange color by AO marked by arrows, especially at concentrations of 25 and 50 μg/ml. These results implicated that SLSP could reduce the accumulation of autophagosomes and thus mitigate the formation of autolysosomes.

As displayed in Figures 6B,C, pretreatment with rapamycin increased the percentage of early apoptotic cells while 3-MA and SLSP reduced such apoptosis process. These results indicated that SLSP could effectively suppress the autophagy and apoptosis of H9c2 cells induced by rapamycin.

### SLSP Inhibited Expressions of Proteins Related to Autophagy and Apoptosis Through PI3K/Akt/mTOR Pathway in H9c2 Cells

**Figure 7:**
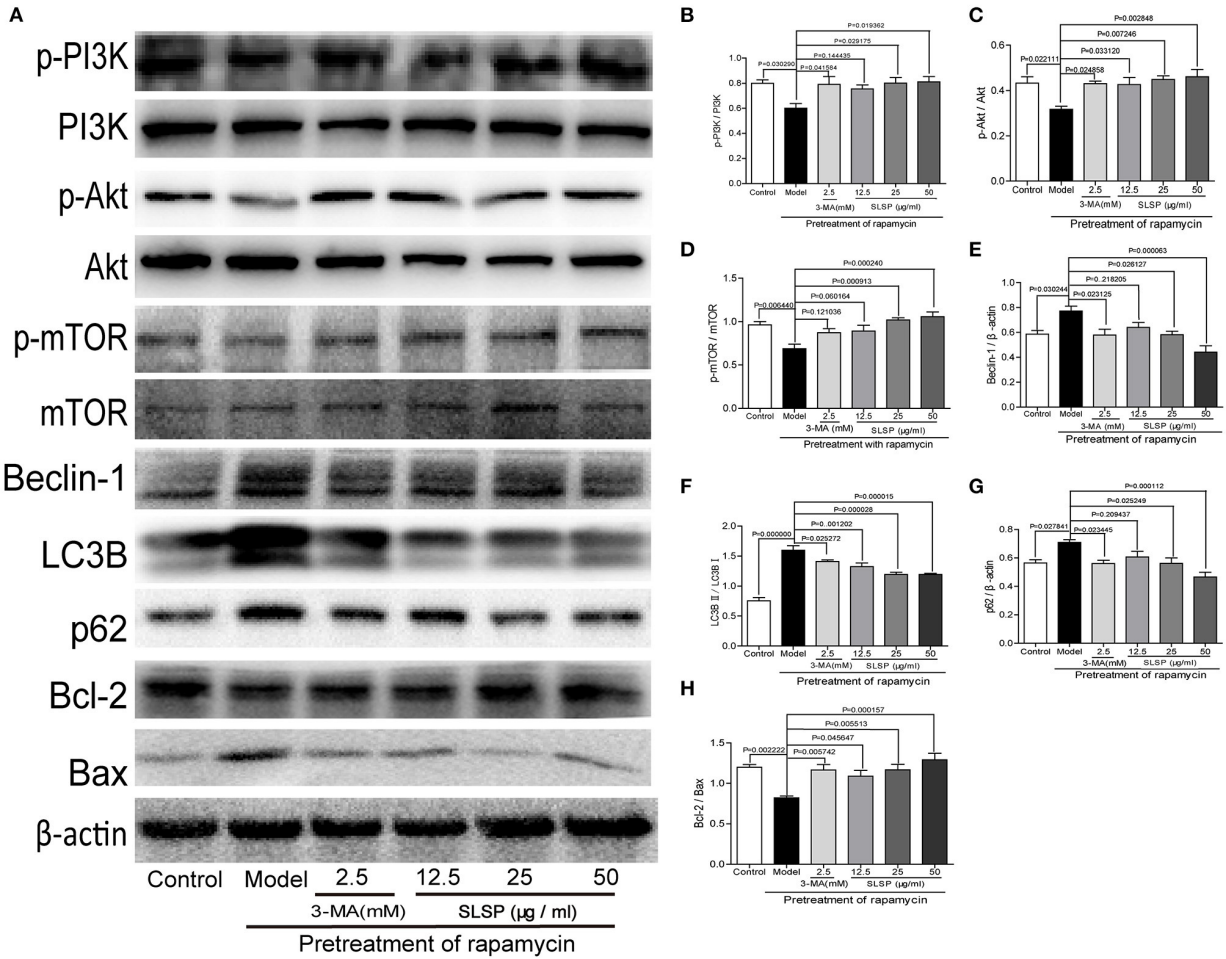
SLSP inhibited the abnormal expressions of proteins related to autophagy and apoptosis in H9c2 cells pretreated with rapamycin. **(A)** Western blotting bands of related proteins in H9c2 cells. **(B–H)** Gray intensity analysis. *N* = 5/group.

In H9c2 cells, rapamycin induced significant changes in proteins involved in autophagy and apoptosis. As shown in Figure 7, rapamycin decreased the phosphorylation of PI3K, Akt, and mTOR (*P* = 0.030290, *P* = 0.022111, and *P* = 0.006440), as well as the ratio of Bcl-2 to Bax (*P* = 0.002222), while markedly increased the expressions of Beclin-1 and p62, and the ratio of LC3BII to LC3B (*P* = 0.030244, *P* = 0.027841, and *P* = 0.000000). These results suggested that rapamycin induced excessive autophagy and apoptosis in H9c2 cells. SLSP especially at higher concentrations reversed the autophagy induced by rapamycin, and it could also alleviate apoptosis of H9c2 cells through regulating PI3K/Akt/mTOR signaling pathway.

## Discussion

SD has been demonstrated to cause a significant increase in cardiac contractility, blood pressure, heart rate, and stress hormone secretion (22). ANP is one of the markers for the clinical diagnosis of hypertrophy and cardiac insufficiency, which is also relevant to hypertension, myocardial infarction, cardiomyopathy, and myocardial interstitial fibrosis (23). When ventricular volume load and pressure overload increase, ANP is released from the cardiac myocytes. LDH plays an important catalytic role in the occurrence and development of cardiovascular diseases (24), which reflects the permeability and the damage extent of the sarcolemma (25). In the present study, the pathological changes of SD mice, such as nuclear shrinkage and enlarged intercellular space accumulated comparing with the control group. SLSP treatment, especially at 50 and 100 mg/kg inhibited such pathological changes. Moreover, SLSP treatment alleviated the increase of heart rate, serum ANP, and serum LDH and elevated ejection fraction in SD mice. These results revealed the protective effect of SLSP on myocardium in SD mice.

Autophagy is a major factor in the regulation of cardiac homeostasis under basal and stress conditions (25). Beclin-1 regulates autophagy and increases autophagy-related protein expression that includes factors such as light chain 3 protein-II (LC3-II) (26). A p62 is a marker that can be used to assess the rate of autophagic flux in cells, as the high level of this protein is linked to the decreased autophagy (27). Excessive autophagy was shown to promote apoptosis through the degradation of anti-apoptotic and cell survival factors (9). The interaction between Beclin-1 and Bcl-2 inhibits the autophagy function of the Beclin-1 class III PI3K complex (28). Therefore, modulation of myocardial autophagy may be one of the efficacious strategies for the prevention of myocardial injury. In the present study, SLSP was found to prevent the increased expressions of LC3B, Beclin-1, and p62, as well as the number of autophagosomes in the heart tissue of mice. Similar results were obtained in rapamycin induced H9c2 cells. Meanwhile, it also mitigated the apoptosis of the injured myocardial cells both *in vivo* and *in vitro*, as shown by increased Bcl-2 but decreased Bax. And SLSP markedly reduced the number of apoptotic H9c2 cells induced by rapamycin. These results indicated that SLSP might protect the myocardial cells against SD induced injury through suppressing excessive autophagy and apoptosis.

**Figure 8:**
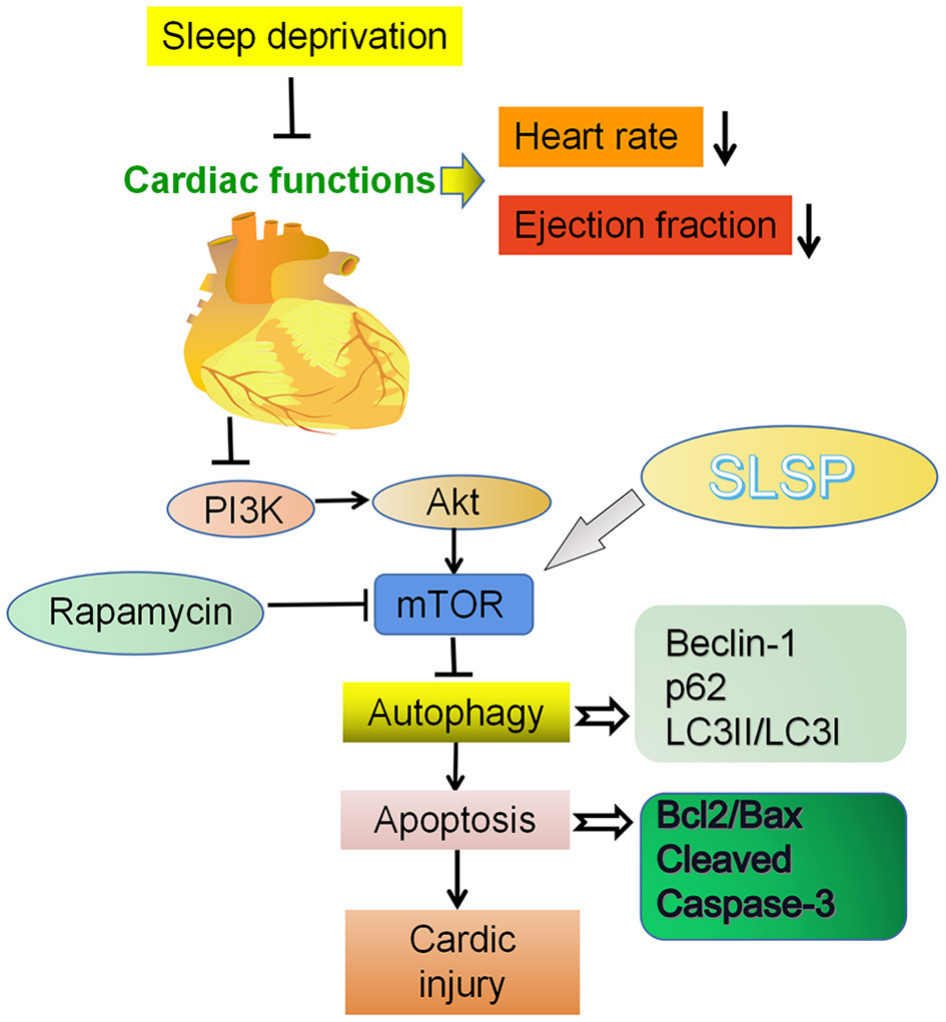
Cardioprotective effect of SLSP on a sleep deprived mice by inhibiting abnormal autophagy through PI3K/Akt/mTOR pathway.

Autophagy is regulated by diverse signaling pathways and factors (29), and nutrient sensor mTOR is likely the core regulator of the process (30, 31). mTOR is modulated by PI3K/Akt pathway, and the activation of the PI3K/Akt/mTOR pathway could inhibit excessive autophagy and block the cardiac disease progress (32). In the present study, SLSP was found to activate the suppressed PI3K/Akt/mTOR pathway in injured myocardial cells induced by SD and rapamycin, implicating that SLSP might inhibit the excessive autophagy by regulating the PI3K/Akt/mTOR signaling pathway (Figure 8). However, the exact mechanism of SLSP in the activation of the PI3K/Akt/mTOR pathway still needs further investigation.

In conclusion, the present study demonstrated that SLSP exerted cardiac protection in SD mice by inhibiting aberrant autophagy and apoptosis through the PI3K/Akt/mTOR signaling pathway.

## Data Availability Statement

The original contributions presented in the study are included in the article/Supplementary Material, further inquiries can be directed to the corresponding author/s.

## Ethics Statement

The animal study was reviewed and approved by Experimental Animal committee of Anhui University of ChineseMedicine.

## Author Contributions

YC, XW, and ZW: conception and design. YC, XW, ZK, YY, QL, SC, and ML: development of methodology. YC, XW, WF, HW, and JY: acquisition of data. YC and XW: analysis and interpretation of data. YC and XW: writing, review, and revision of the manuscript. XW and ZW: study supervision. All authors contributed to the article and approved the submitted version.

## Funding

This work was financially supported by the National Natural Science Foundation of China (81530096), Shanghai E-Research Institute of Bioactive Constituent in TCM plan, the Opening Project of Shanghai Key Laboratory of Compound Chinese Medicines (17DZ2273300), Youth Project of Anhui Natural Science Foundation (2108085QH372), Key Program for International Cooperation and Exchange of the National Natural Science Foundation of China (81920108033).

## Conflict of Interest

YY was employed by company Kanion Pharmaceutical Co., Ltd. The remaining authors declare that the research was conducted in the absence of any commercial or financial relationships that could be construed as a potential conflict of interest.

## Publisher's Note

All claims expressed in this article are solely those of the authors and do not necessarily represent those of their affiliated organizations, or those of the publisher, the editors and the reviewers. Any product that may be evaluated in this article, or claim that may be made by its manufacturer, is not guaranteed or endorsed by the publisher.

## Abbreviations

3-MA: 3-methyladenine
mTOR: mammalian target of rapamycin
p62: ubiquitin-binding protein p62 or sequestosome-1
p-Akt: phosphorylated PKB
p-PI3K: phosphorylated PI3K
SD: sleep deprivation
SLSP: stem-leaf saponins from *Panax notoginseng*.
