# Corrigendum: Cardioprotective Effect of Stem-Leaf Saponins From *Panax notoginseng* on Mice With Sleep Deprivation by Inhibiting Abnormal Autophagy Through PI3K/Akt/mTOR Pathway

**DOI:** 10.3389/fcvm.2021.832174

**Published:** 2022-01-07

**Authors:** Yin Cao, Qinglin Li, Yingbo Yang, Zunji Ke, Shengqi Chen, Mingrui Li, Wenjing Fan, Hui Wu, Jinfeng Yuan, Zhengtao Wang, Xiaojun Wu

**Affiliations:** ^1^Shanghai Key Laboratory of Compound Chinese Medicines, The Ministry of Education (MOE) Key Laboratory for Standardization of Chinese Medicines, The State Administration of TCM (SATCM) Key Laboratory for New Resources and Quality Evaluation of Chinese Medicine, Institute of Chinese Materia Medica, Shanghai University of Traditional Chinese Medicine, Shanghai, China; ^2^Key Laboratory of Xin'an Medicine, Ministry of Education, Anhui Key Laboratory of R&D of Chinese Medicine, Anhui University of Chinese Medicine, Hefei, China; ^3^Kanion Pharmaceutical Co., Ltd, Lianyungang, China; ^4^Academy of Integrative Medicine, Shanghai University of Traditional Chinese medicine, Shanghai, China

**Keywords:** stem-leaf saponin from *Panax notoginseng*, autophagy, sleep deprivation, cardioprotection, apoptosis

There is an error in the Funding statement. The correct number for **Youth Project of Anhui Natural Science Foundation** is **2108085QH372**. The corrected funding statement appears below.

## Funding

This work was financially supported by the National Natural Science Foundation of China (81530096), Shanghai E-Research Institute of Bioactive Constituent in TCM plan, the Opening Project of Shanghai Key Laboratory of Compound Chinese Medicines (17DZ2273300), Youth Project of Anhui Natural Science Foundation (2108085QH372), Key Program for International Cooperation and Exchange of the National Natural Science Foundation of China (81920108033).

There is an error in the article title. The word “derivation” in title should be corrected as “deprivation.”

The authors apologize for these errors and state that this does not change the scientific conclusions of the article in any way. The original article has been updated.

## Publisher's Note

All claims expressed in this article are solely those of the authors and do not necessarily represent those of their affiliated organizations, or those of the publisher, the editors and the reviewers. Any product that may be evaluated in this article, or claim that may be made by its manufacturer, is not guaranteed or endorsed by the publisher.

