# Supplementary figures and images for: Cardioprotective Effect of Stem-Leaf Saponins From Panax notoginseng on Mice With Sleep Deprivation by Inhibiting Abnormal Autophagy Through PI3K/Akt/mTOR Pathway
Source: Front Cardiovasc Med. 2021 Sep 16;8:694219. doi: 10.3389/fcvm.2021.694219 (PMC8483245; doi:10.3389/fcvm.2021.694219)

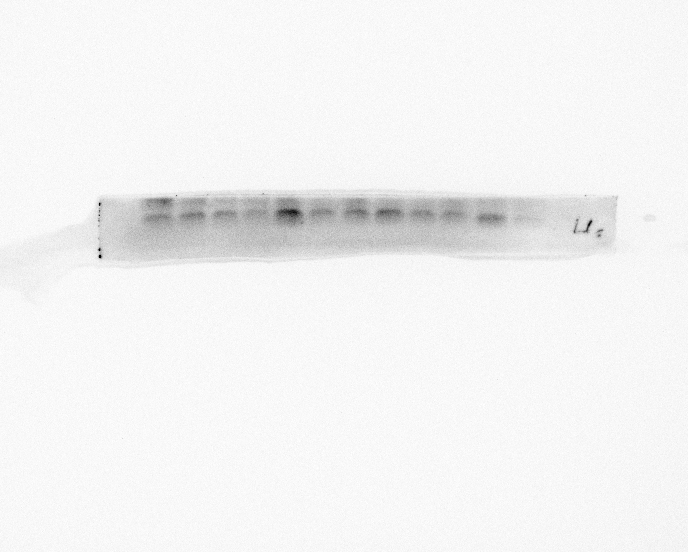

Supplement: Supplementary file 1 [file Data_Sheet_1.ZIP › original WB/SLSP-H9C2 - ╕▒▒╛/LC/LC-1-2.tif]

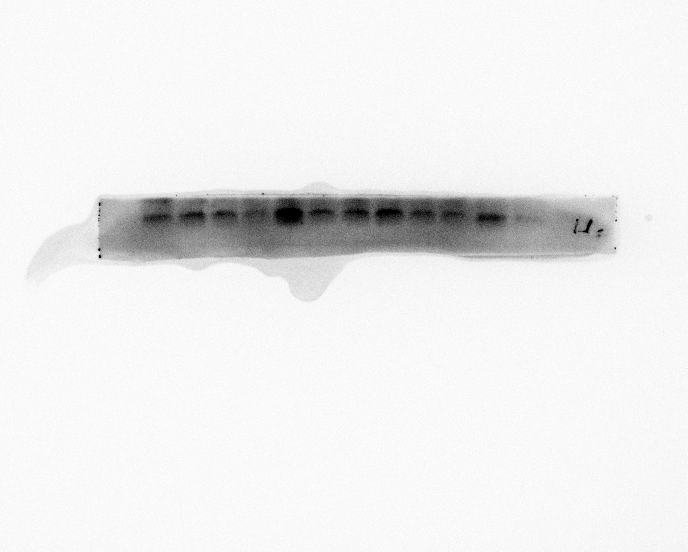

Supplement: Supplementary file 1 [file Data_Sheet_1.ZIP › original WB/SLSP-H9C2 - ╕▒▒╛/LC/LC-2-1.tif]

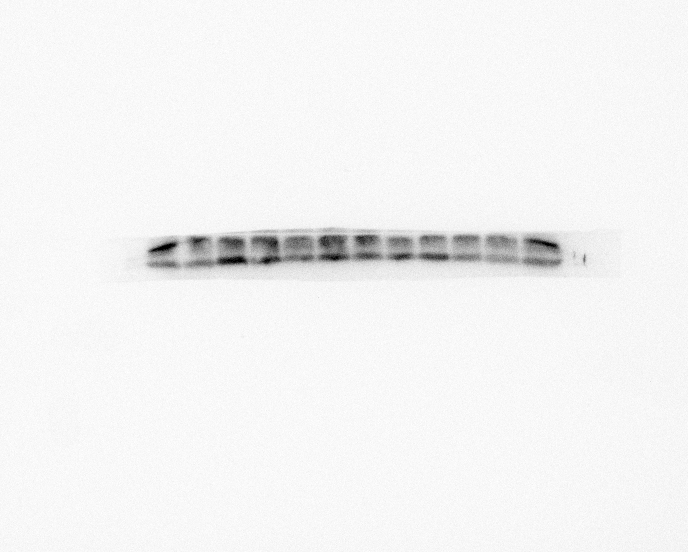

Supplement: Supplementary file 1 [file Data_Sheet_1.ZIP › original WB/SLSP-H9C2 - ╕▒▒╛/LC/LC-2-2.tif]

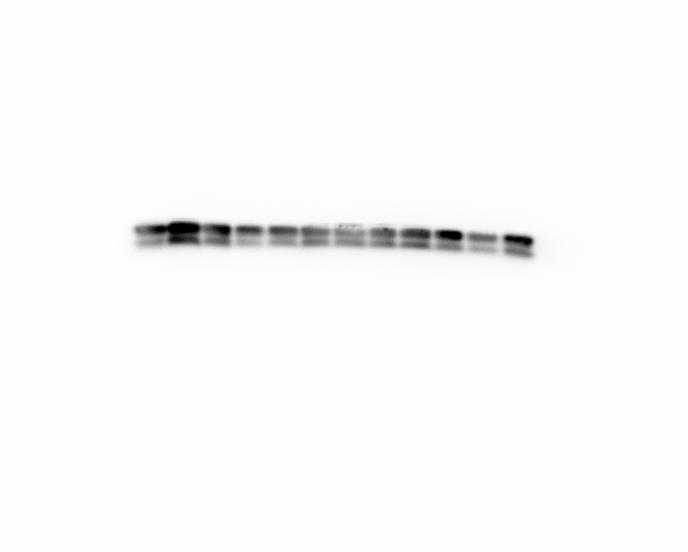

Supplement: Supplementary file 1 [file Data_Sheet_1.ZIP › original WB/SLSP-H9C2 - ╕▒▒╛/LC/lc-3-2.tif]

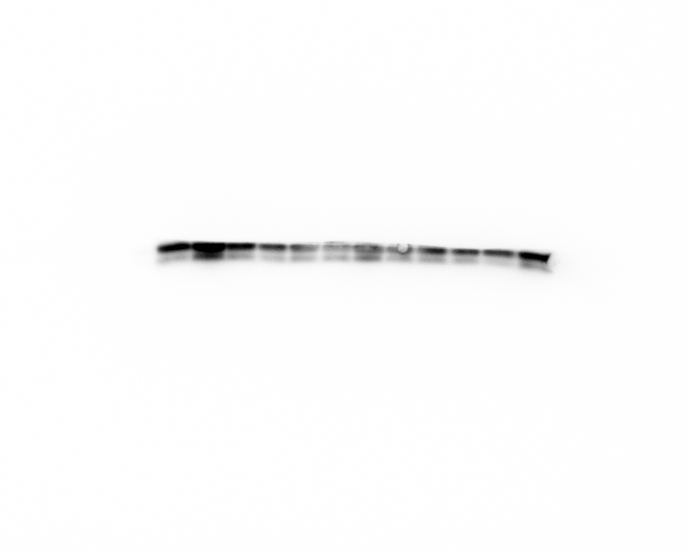

Supplement: Supplementary file 1 [file Data_Sheet_1.ZIP › original WB/SLSP-H9C2 - ╕▒▒╛/LC/lc-4.jpg]

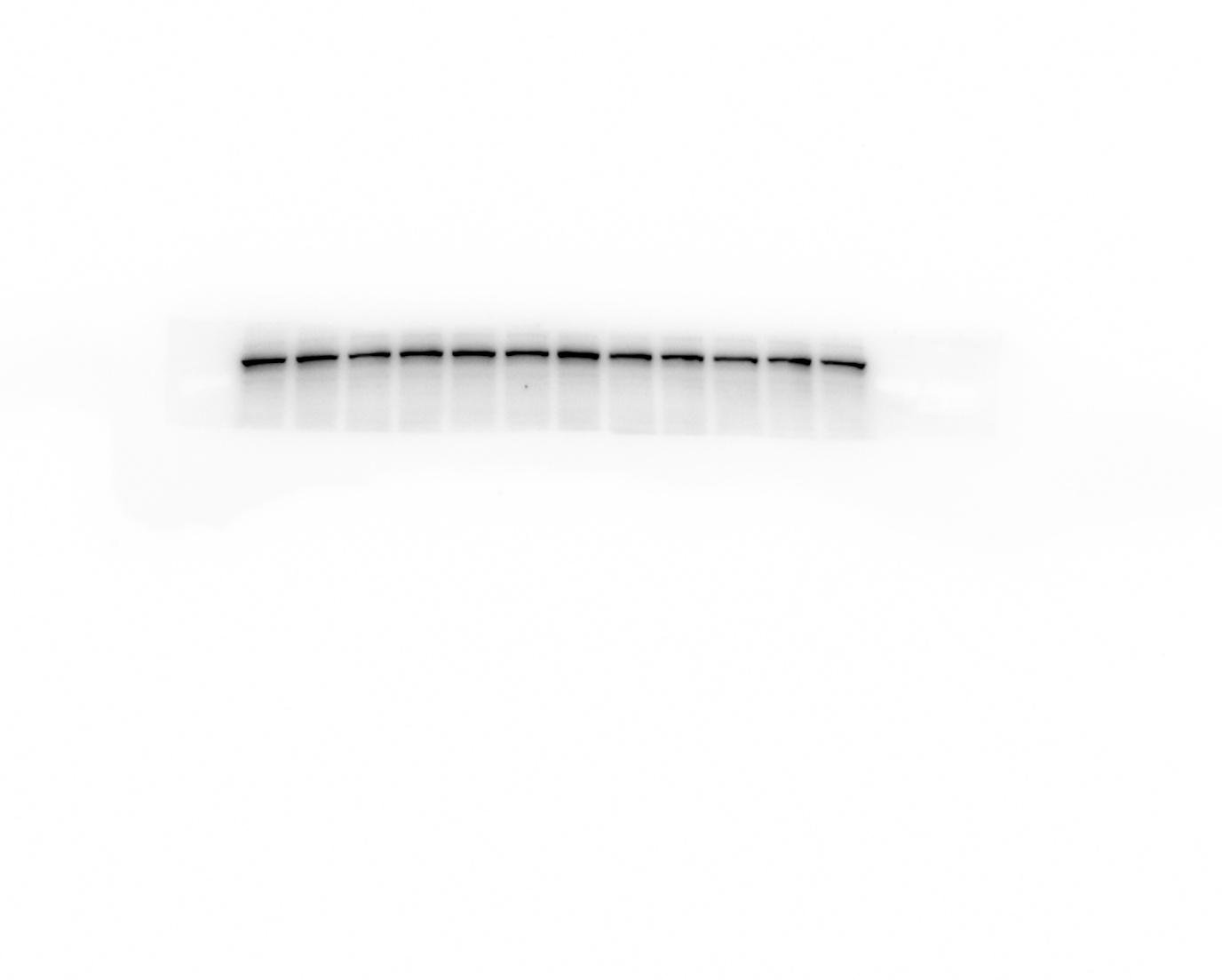

Supplement: Supplementary file 1 [file Data_Sheet_1.ZIP › original WB/SLSP-H9C2 - ╕▒▒╛/PI3K/PI3K-1.Tif]

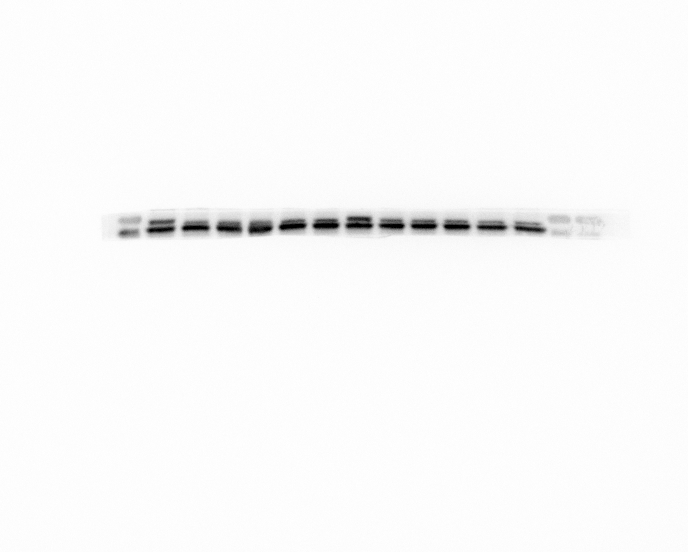

Supplement: Supplementary file 1 [file Data_Sheet_1.ZIP › original WB/SLSP-H9C2 - ╕▒▒╛/PI3K/PI3K-2.tif]

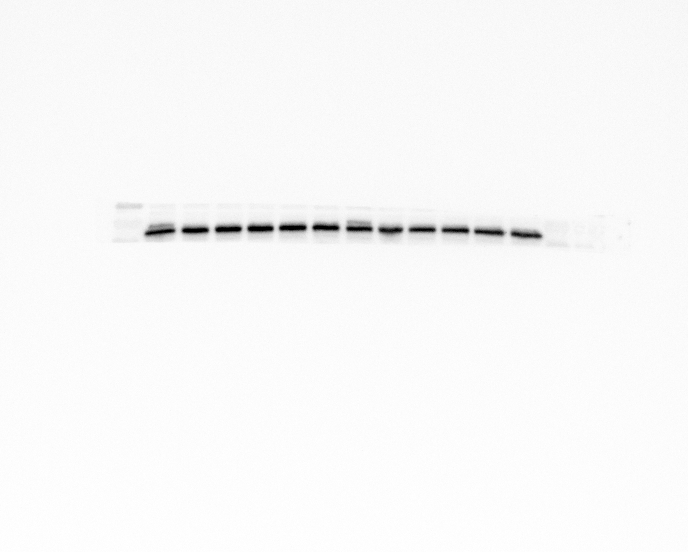

Supplement: Supplementary file 1 [file Data_Sheet_1.ZIP › original WB/SLSP-H9C2 - ╕▒▒╛/PI3K/PI3K-3.tif]

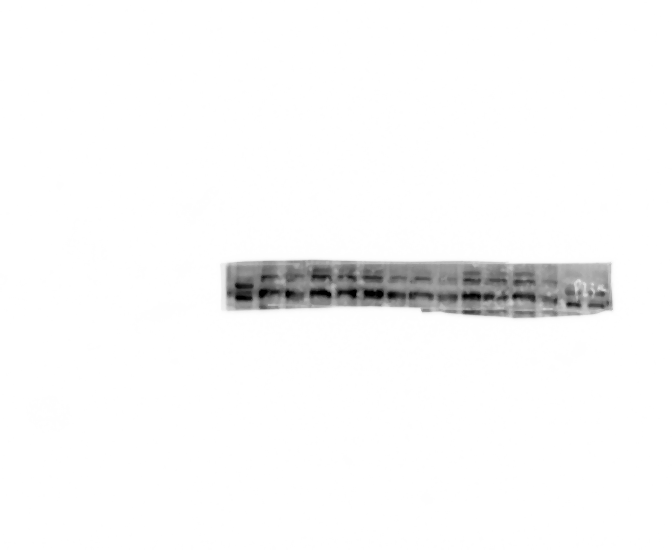

Supplement: Supplementary file 1 [file Data_Sheet_1.ZIP › original WB/SLSP-H9C2 - ╕▒▒╛/PPI3K/001-shine.tif]

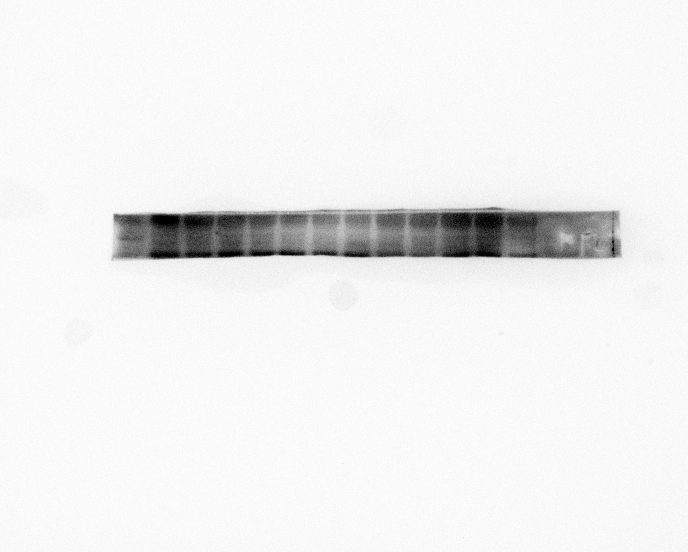

Supplement: Supplementary file 1 [file Data_Sheet_1.ZIP › original WB/SLSP-H9C2 - ╕▒▒╛/PPI3K/PPI3K.tif]

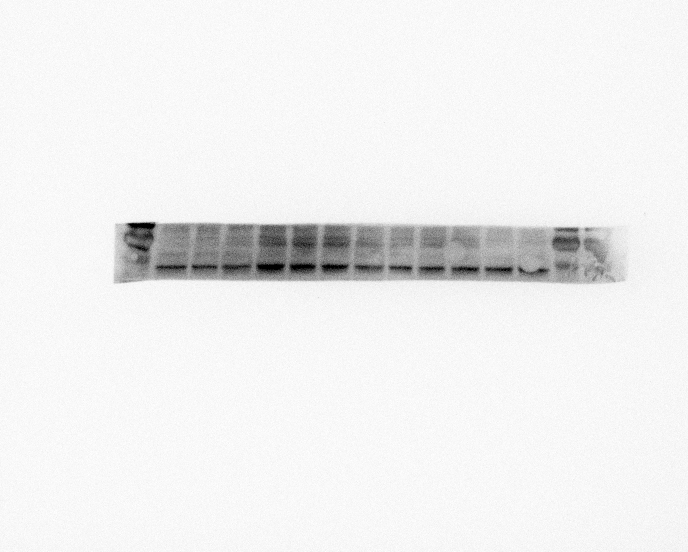

Supplement: Supplementary file 1 [file Data_Sheet_1.ZIP › original WB/SLSP-H9C2 - ╕▒▒╛/PPI3K/ppi3k-2-2.tif]

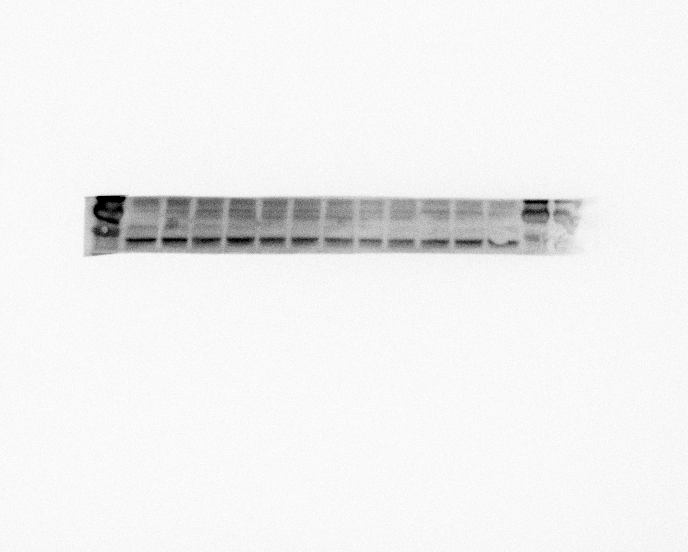

Supplement: Supplementary file 1 [file Data_Sheet_1.ZIP › original WB/SLSP-H9C2 - ╕▒▒╛/PPI3K/ppi3k-2.tif]

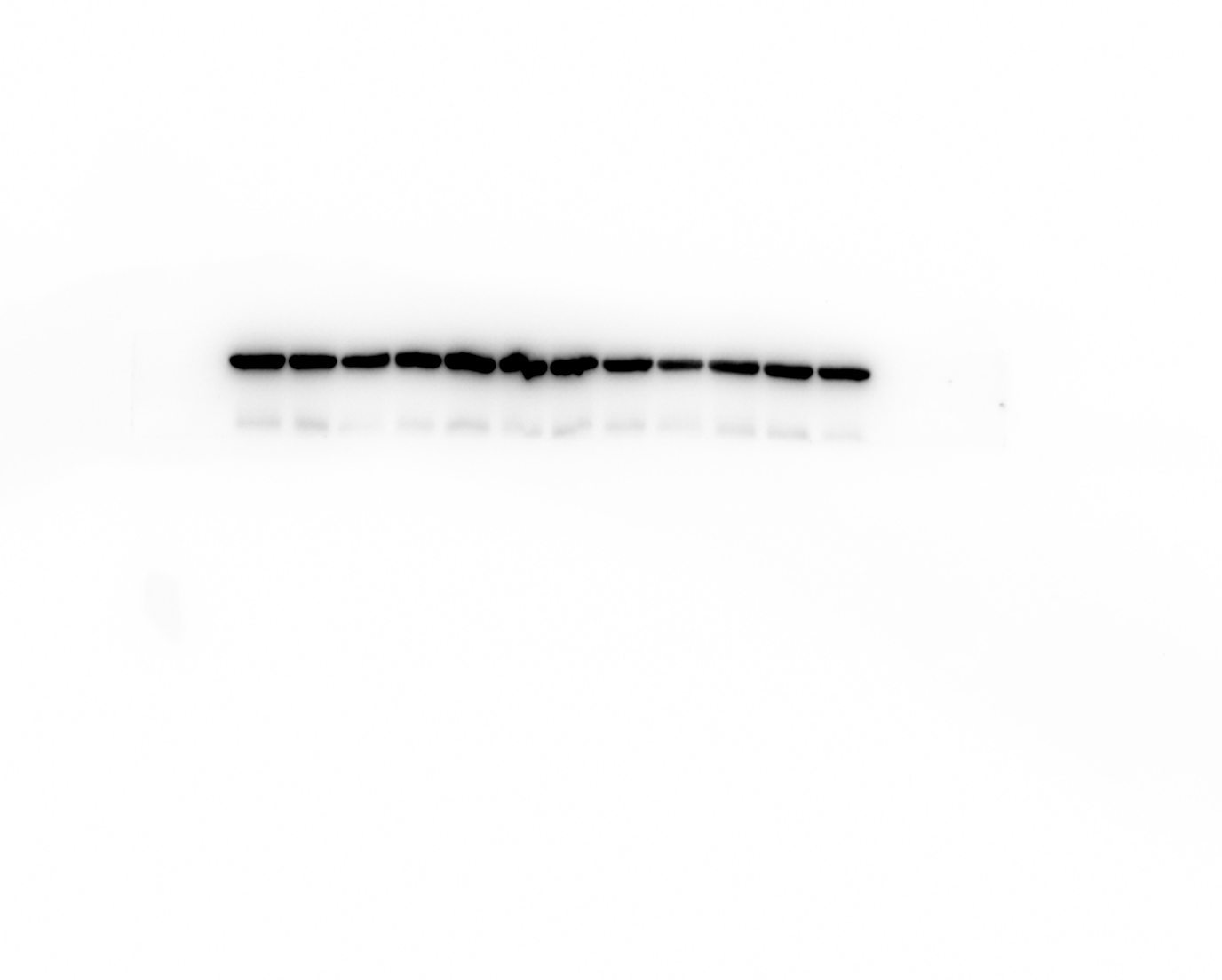

Supplement: Supplementary file 1 [file Data_Sheet_1.ZIP › original WB/SLSP-H9C2 - ╕▒▒╛/actin/ACTIN-1.Tif]

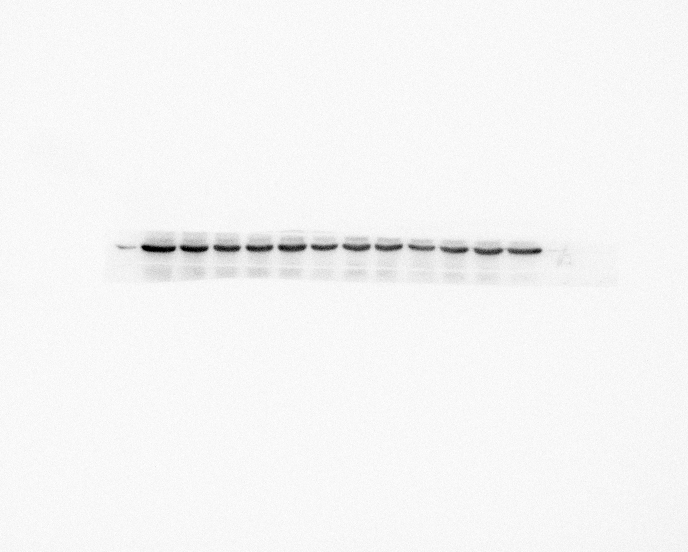

Supplement: Supplementary file 1 [file Data_Sheet_1.ZIP › original WB/SLSP-H9C2 - ╕▒▒╛/actin/ACTIN.tif]

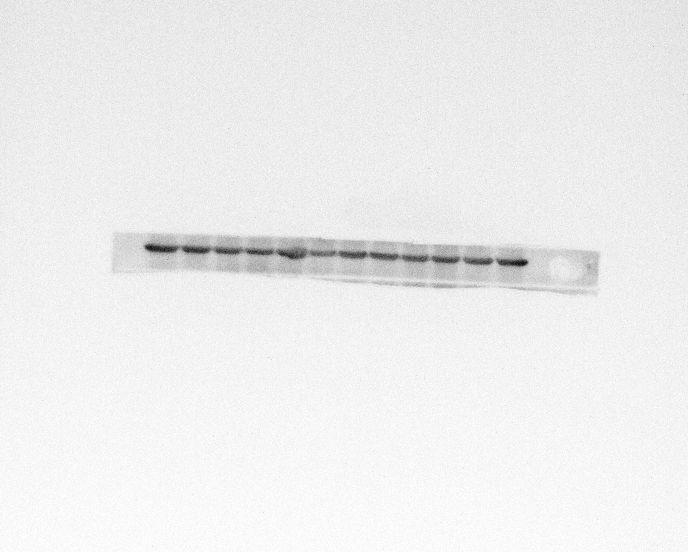

Supplement: Supplementary file 1 [file Data_Sheet_1.ZIP › original WB/SLSP-H9C2 - ╕▒▒╛/actin/actin-1-2.tif]

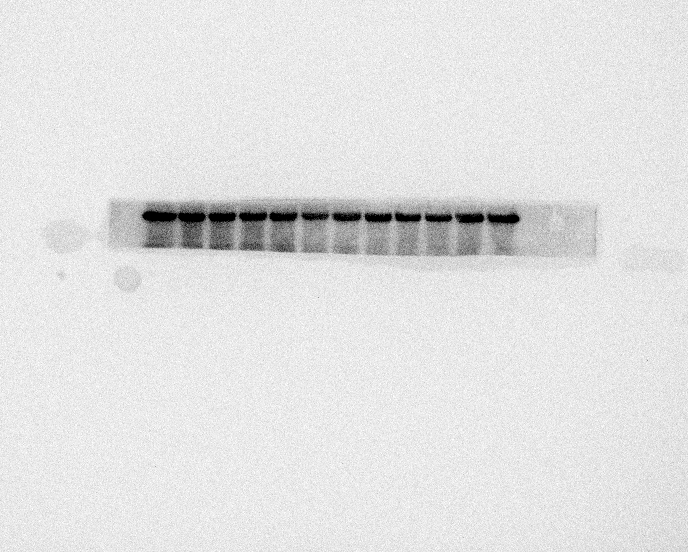

Supplement: Supplementary file 1 [file Data_Sheet_1.ZIP › original WB/SLSP-H9C2 - ╕▒▒╛/actin/actin-2-2.tif]

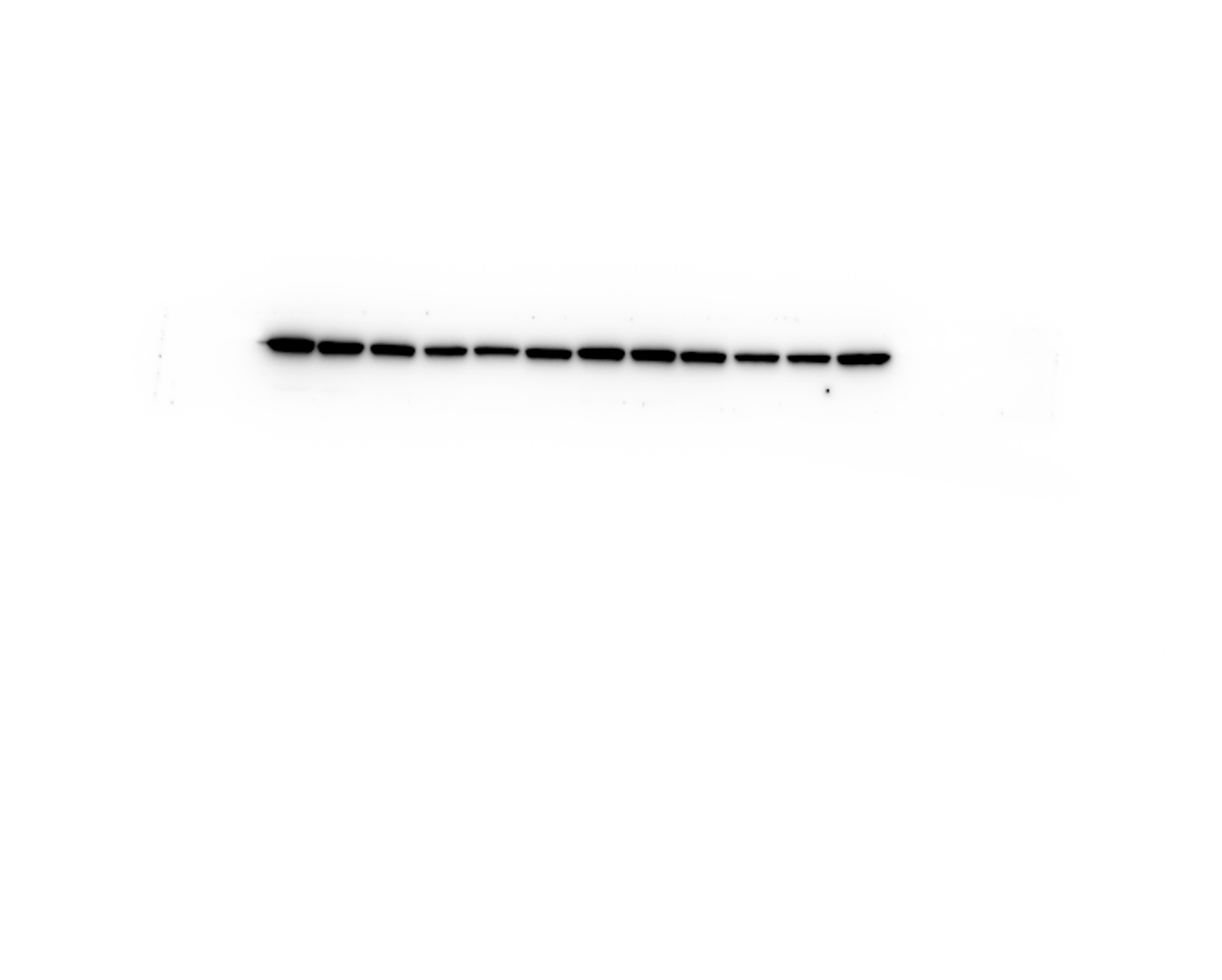

Supplement: Supplementary file 1 [file Data_Sheet_1.ZIP › original WB/SLSP-H9C2 - ╕▒▒╛/akt/AKT-1-1.Tif]

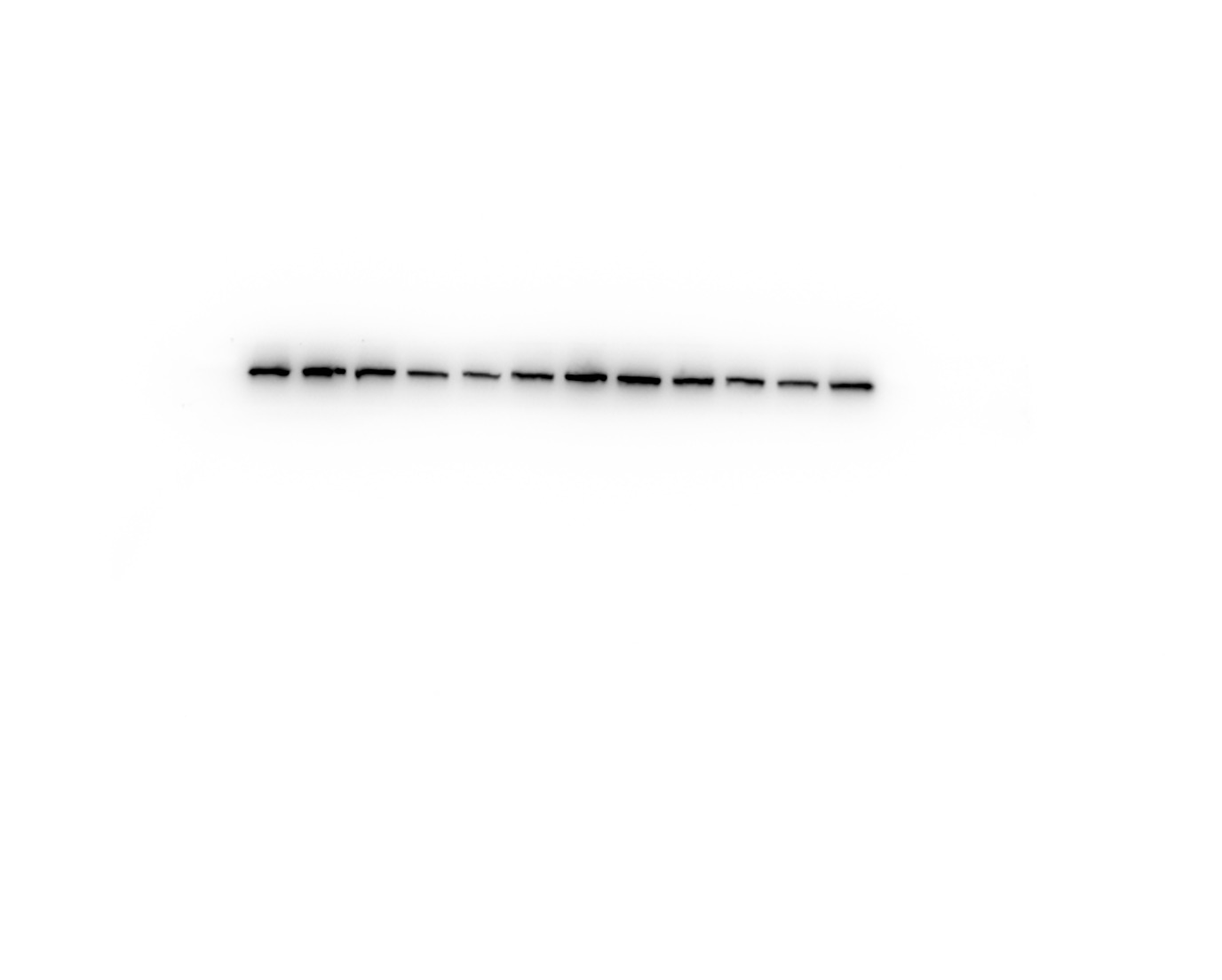

Supplement: Supplementary file 1 [file Data_Sheet_1.ZIP › original WB/SLSP-H9C2 - ╕▒▒╛/akt/AKT-1.Tif]

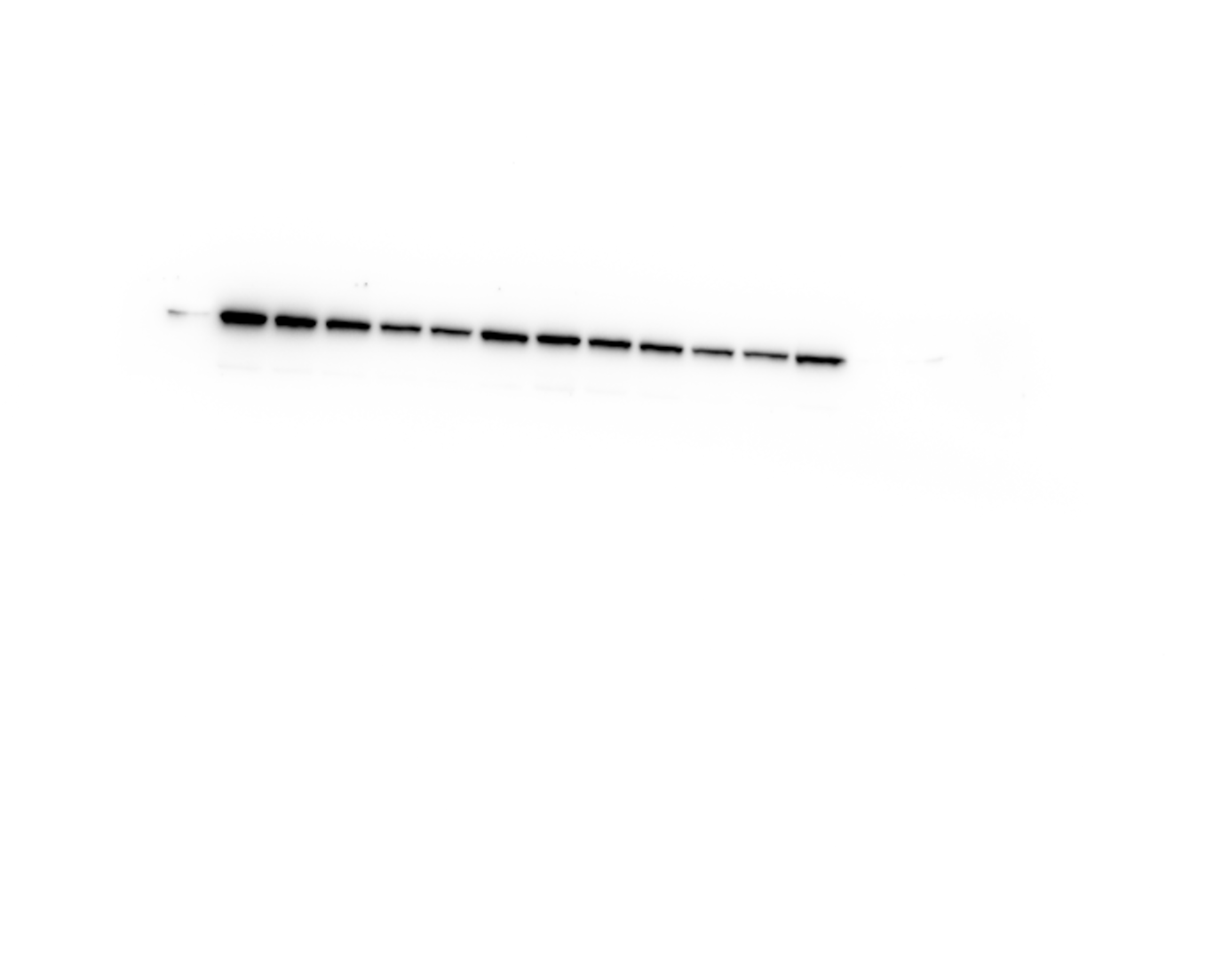

Supplement: Supplementary file 1 [file Data_Sheet_1.ZIP › original WB/SLSP-H9C2 - ╕▒▒╛/akt/AKT-2-1.Tif]

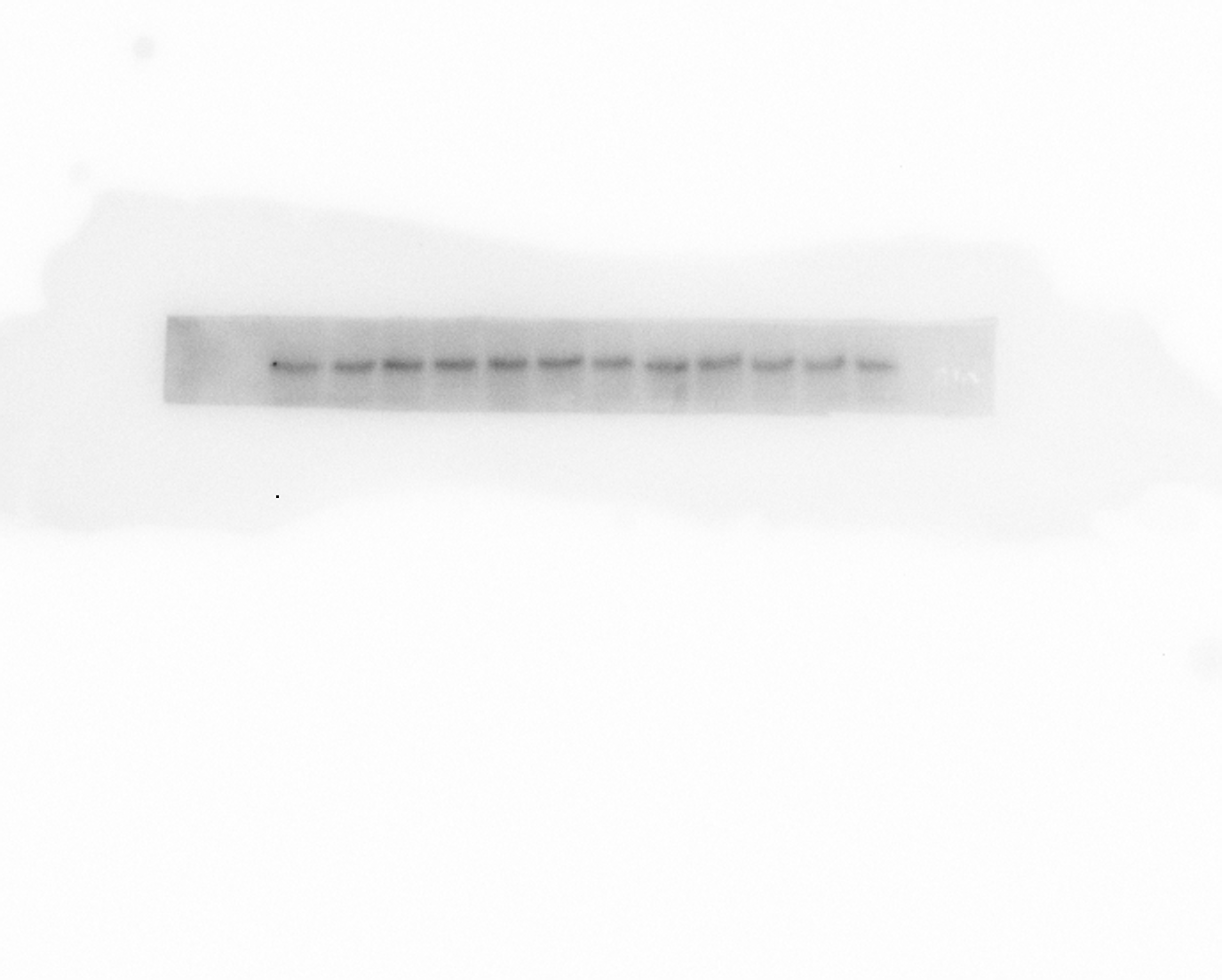

Supplement: Supplementary file 1 [file Data_Sheet_1.ZIP › original WB/SLSP-H9C2 - ╕▒▒╛/bax/BAX-1.Tif]

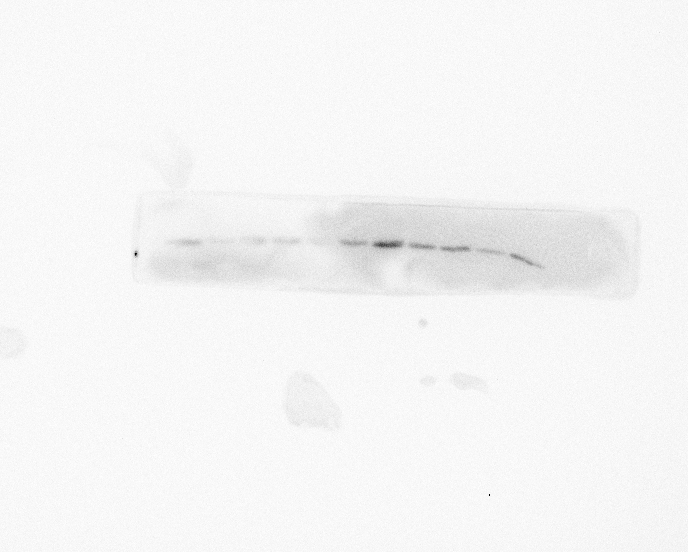

Supplement: Supplementary file 1 [file Data_Sheet_1.ZIP › original WB/SLSP-H9C2 - ╕▒▒╛/bax/BAX.tif]

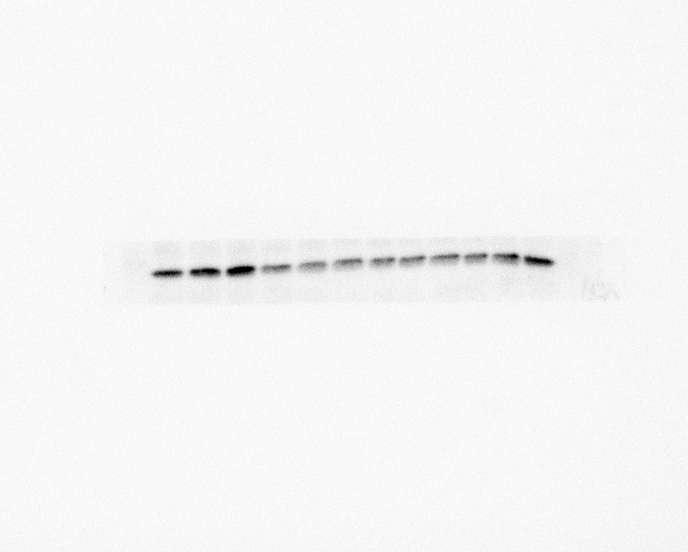

Supplement: Supplementary file 1 [file Data_Sheet_1.ZIP › original WB/SLSP-H9C2 - ╕▒▒╛/bax/bax-1-2.tif]

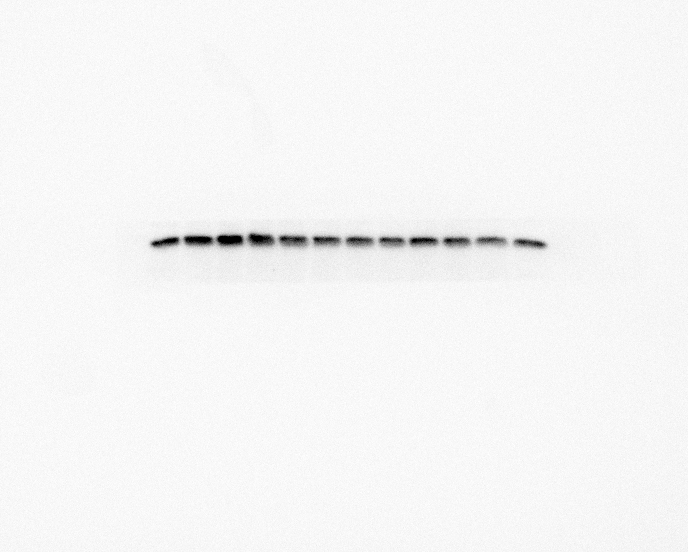

Supplement: Supplementary file 1 [file Data_Sheet_1.ZIP › original WB/SLSP-H9C2 - ╕▒▒╛/bax/bax-2-2.tif]

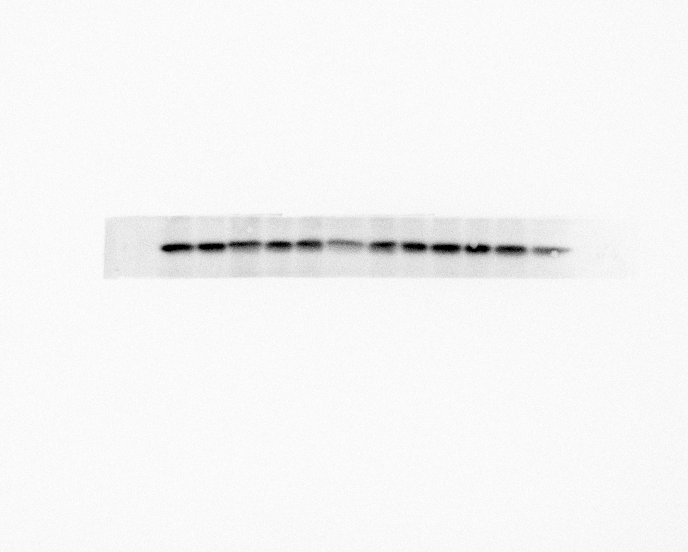

Supplement: Supplementary file 1 [file Data_Sheet_1.ZIP › original WB/SLSP-H9C2 - ╕▒▒╛/bax/bax-2.tif]

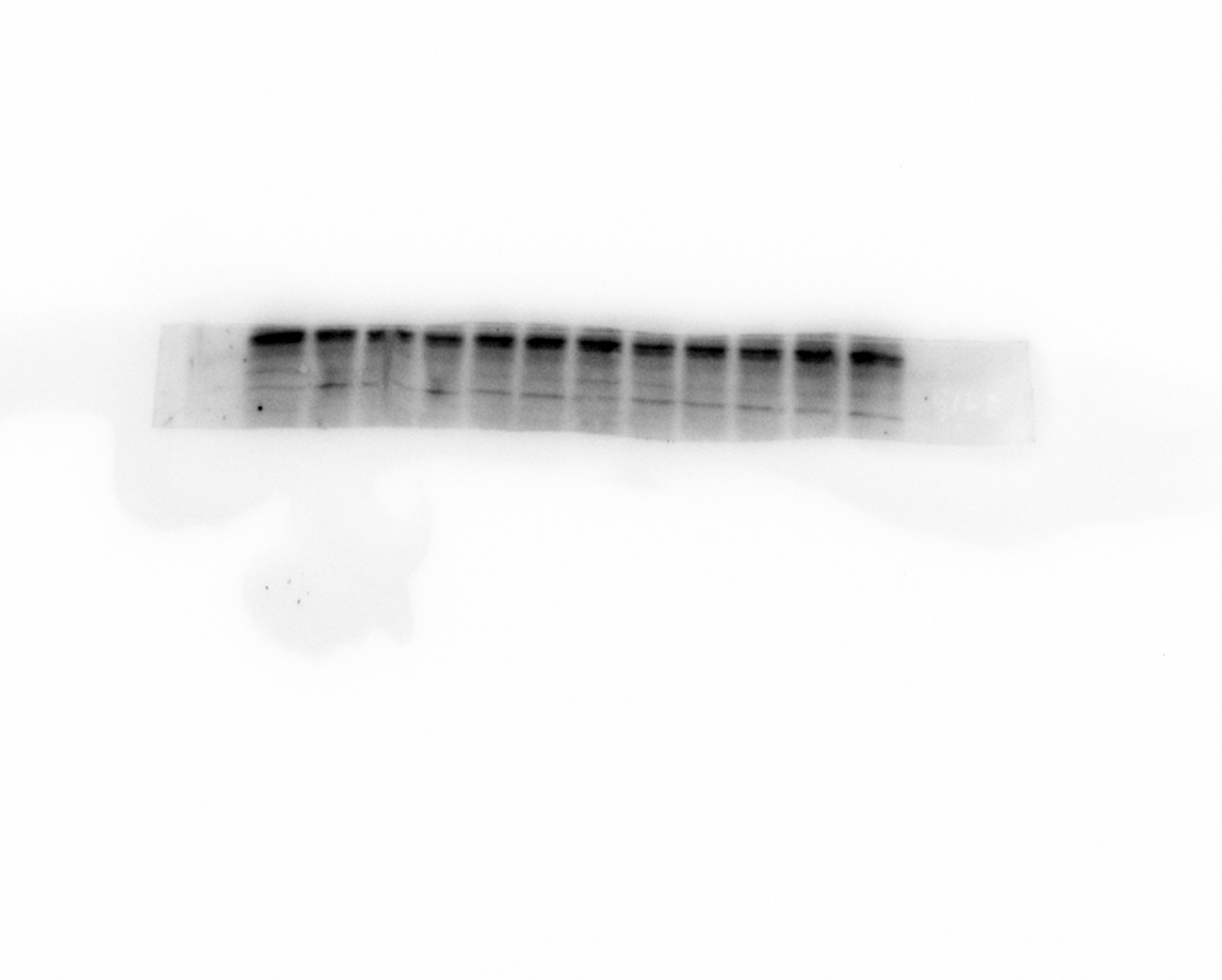

Supplement: Supplementary file 1 [file Data_Sheet_1.ZIP › original WB/SLSP-H9C2 - ╕▒▒╛/bcl2/BCL2-1-1.Tif]

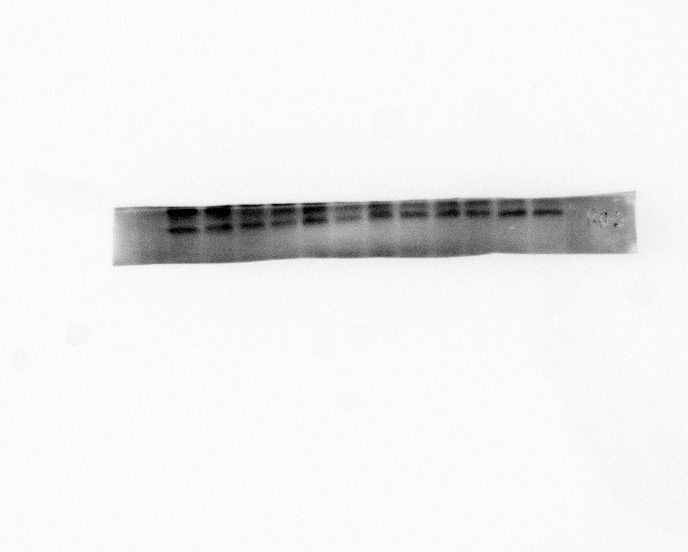

Supplement: Supplementary file 1 [file Data_Sheet_1.ZIP › original WB/SLSP-H9C2 - ╕▒▒╛/bcl2/BCL2-2-2.tif]

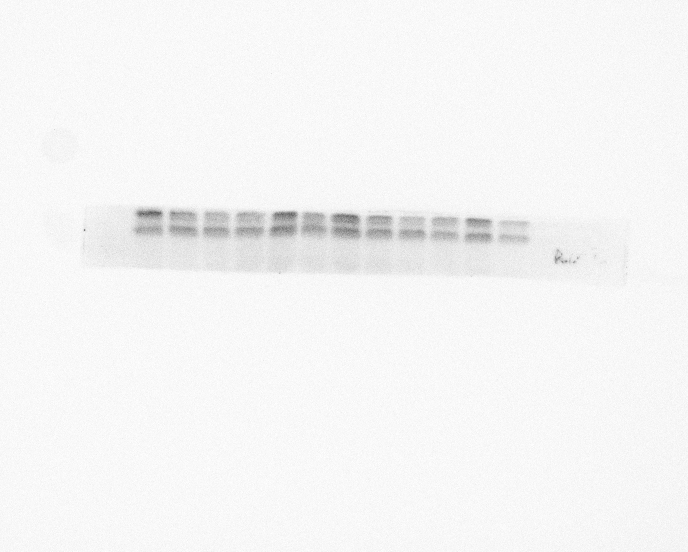

Supplement: Supplementary file 1 [file Data_Sheet_1.ZIP › original WB/SLSP-H9C2 - ╕▒▒╛/bcl2/BCL2-2-3.tif]

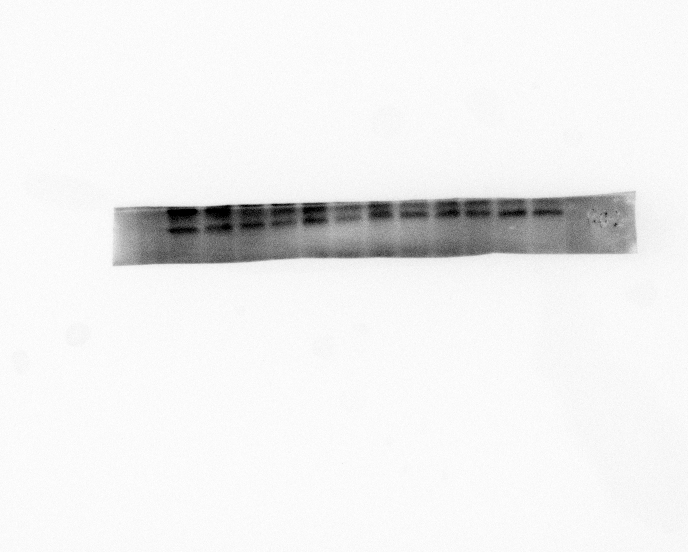

Supplement: Supplementary file 1 [file Data_Sheet_1.ZIP › original WB/SLSP-H9C2 - ╕▒▒╛/bcl2/BCL2-2-4.jpg]

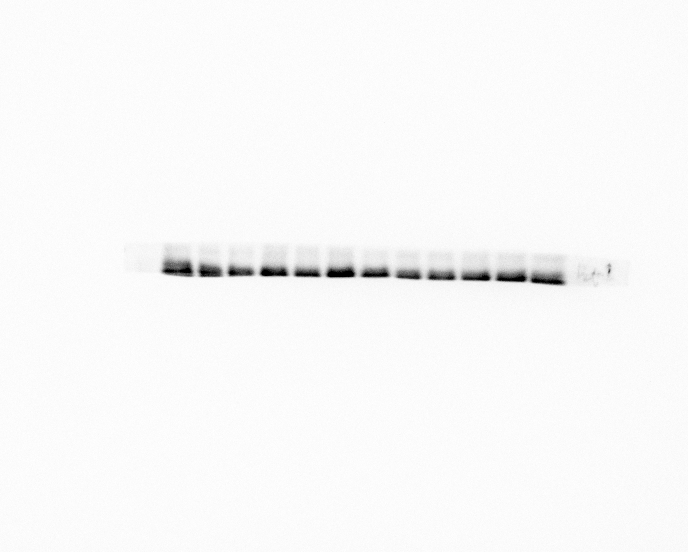

Supplement: Supplementary file 1 [file Data_Sheet_1.ZIP › original WB/SLSP-H9C2 - ╕▒▒╛/bcl2/bcl2-3-2.tif]

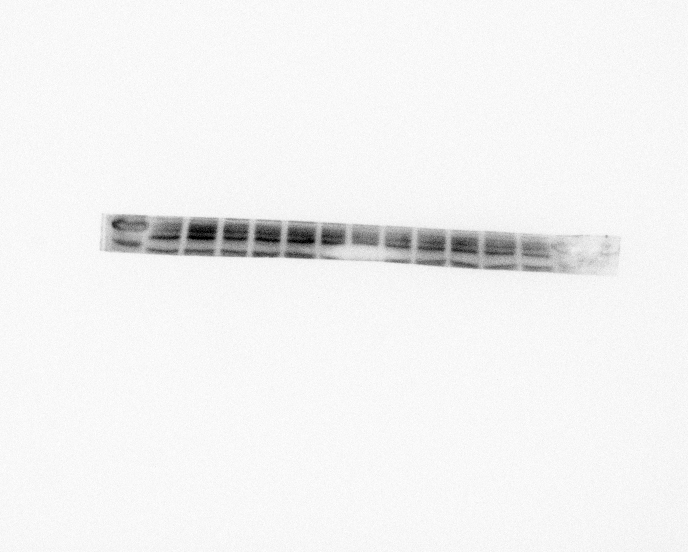

Supplement: Supplementary file 1 [file Data_Sheet_1.ZIP › original WB/SLSP-H9C2 - ╕▒▒╛/beclin-1/bec-2.tif]

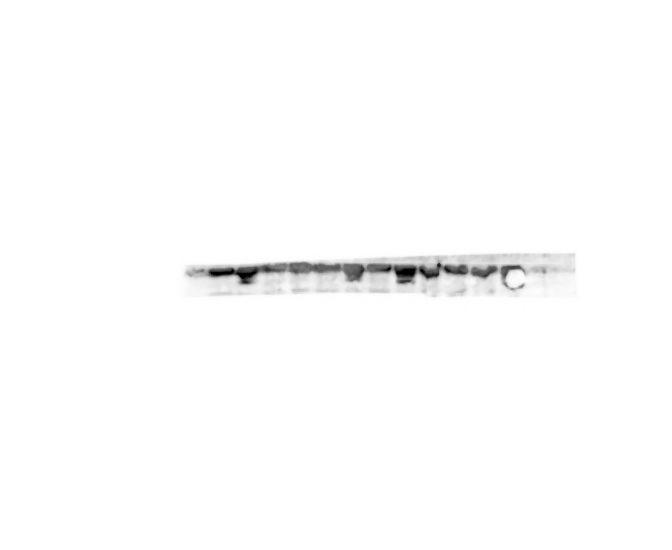

Supplement: Supplementary file 1 [file Data_Sheet_1.ZIP › original WB/SLSP-H9C2 - ╕▒▒╛/beclin-1/beclin-1.tif]

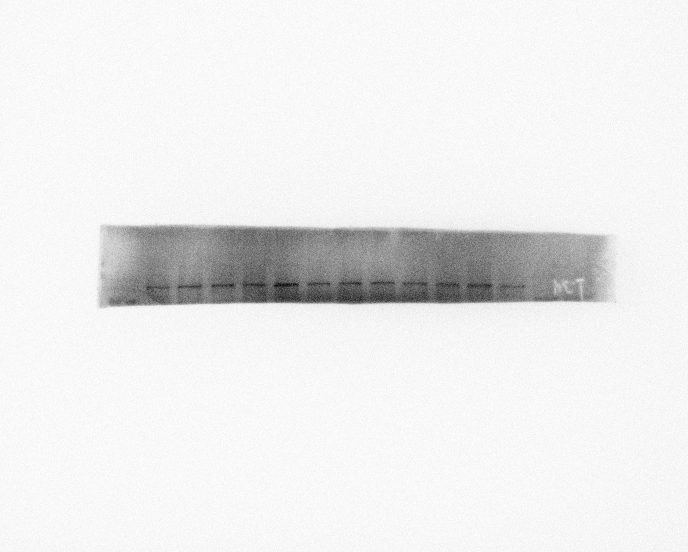

Supplement: Supplementary file 1 [file Data_Sheet_1.ZIP › original WB/SLSP-H9C2 - ╕▒▒╛/mtor/M1-2.tif]

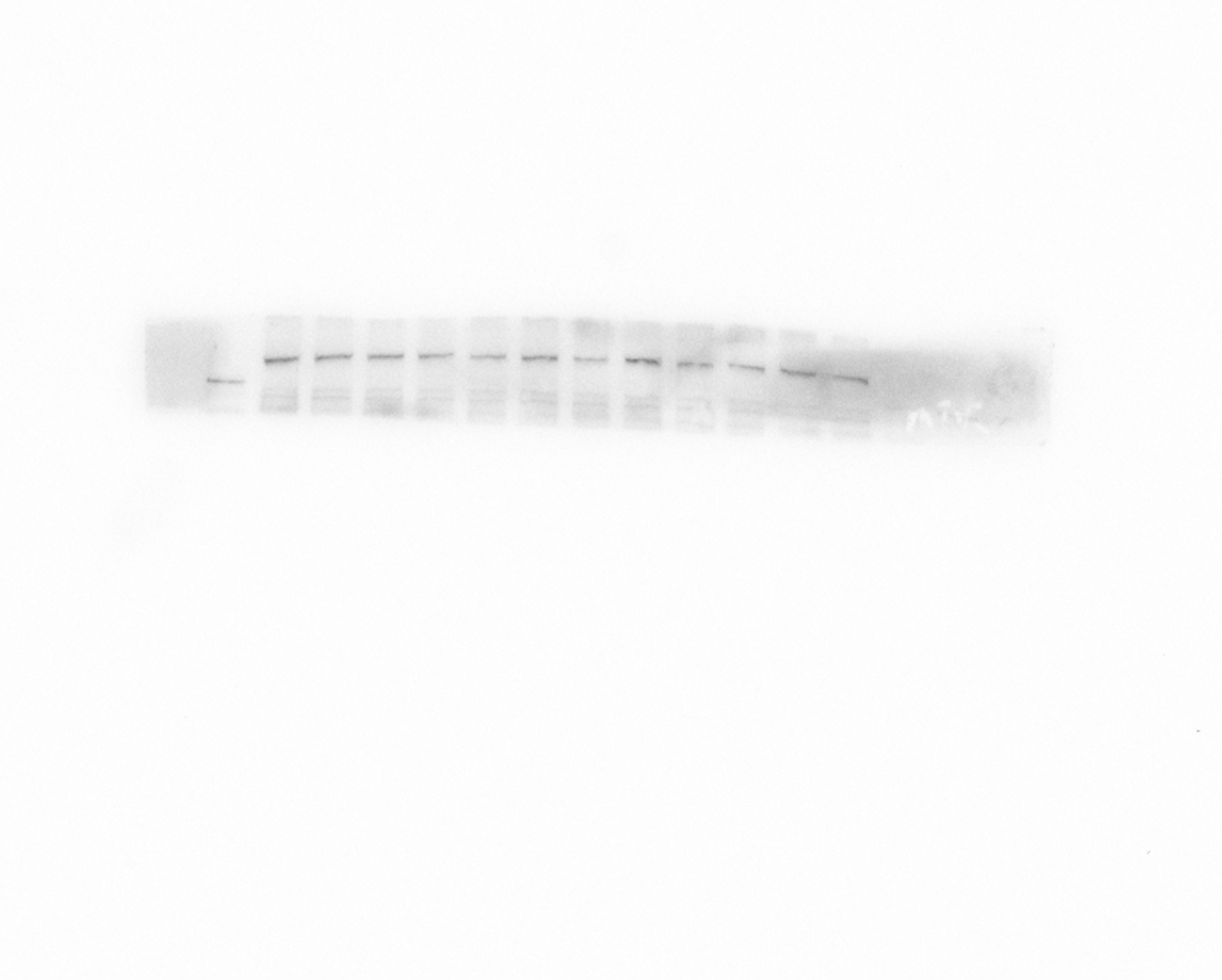

Supplement: Supplementary file 1 [file Data_Sheet_1.ZIP › original WB/SLSP-H9C2 - ╕▒▒╛/mtor/M1-5.Tif]

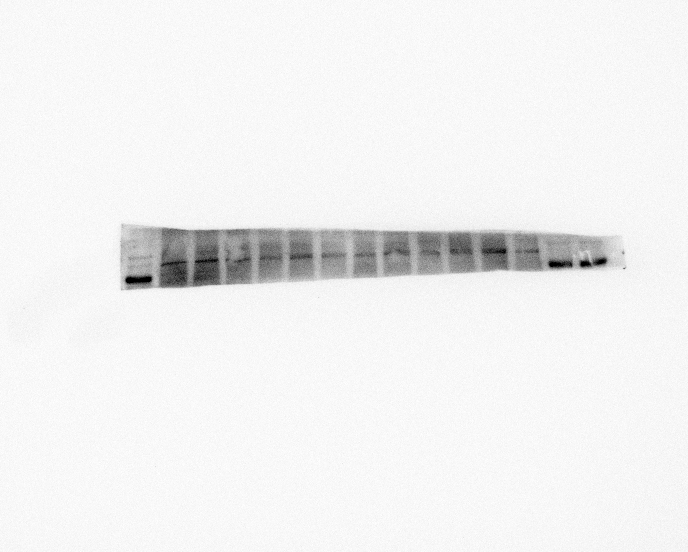

Supplement: Supplementary file 1 [file Data_Sheet_1.ZIP › original WB/SLSP-H9C2 - ╕▒▒╛/mtor/M2.tif]

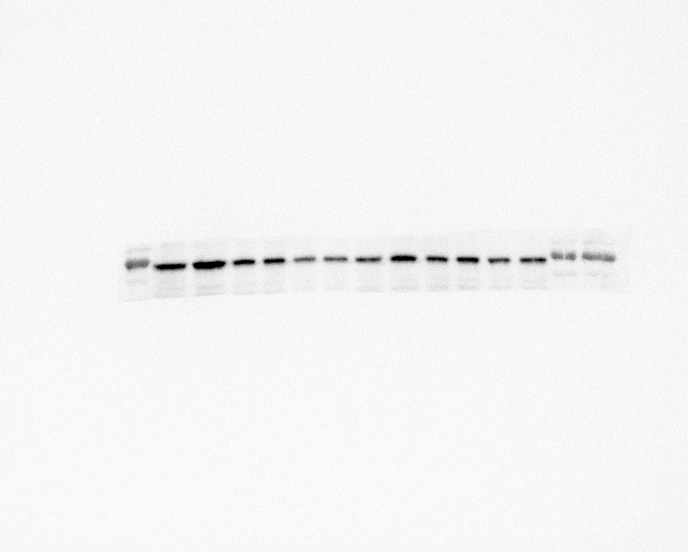

Supplement: Supplementary file 1 [file Data_Sheet_1.ZIP › original WB/SLSP-H9C2 - ╕▒▒╛/p62/P62-4.tif]

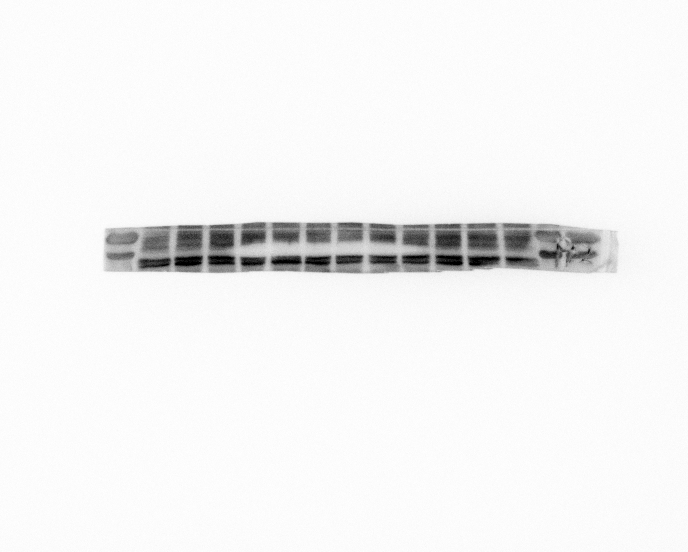

Supplement: Supplementary file 1 [file Data_Sheet_1.ZIP › original WB/SLSP-H9C2 - ╕▒▒╛/p62/p62-2.tif]

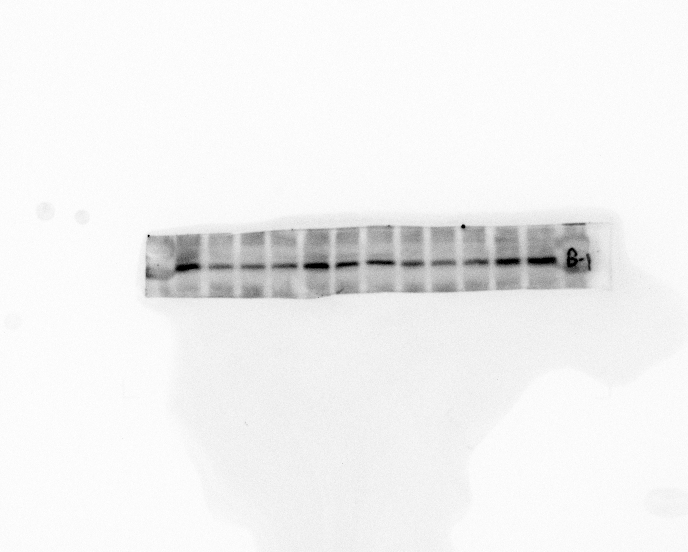

Supplement: Supplementary file 1 [file Data_Sheet_1.ZIP › original WB/SLSP-H9C2 - ╕▒▒╛/pakt/pakt-1.tif]

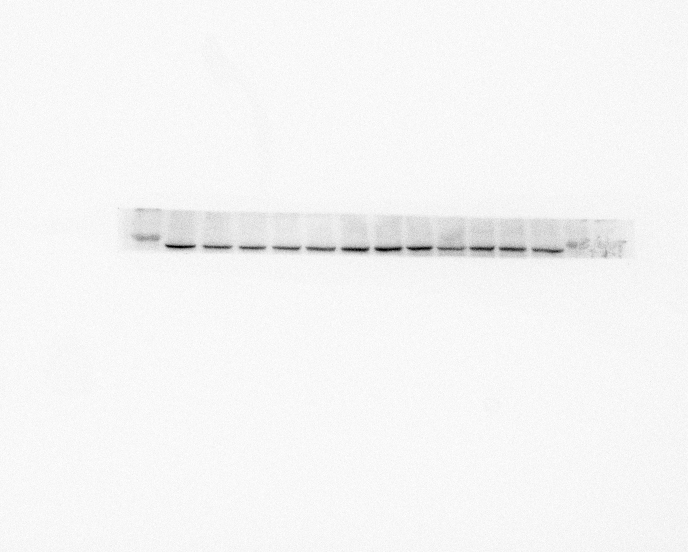

Supplement: Supplementary file 1 [file Data_Sheet_1.ZIP › original WB/SLSP-H9C2 - ╕▒▒╛/pakt/pakt-2.tif]

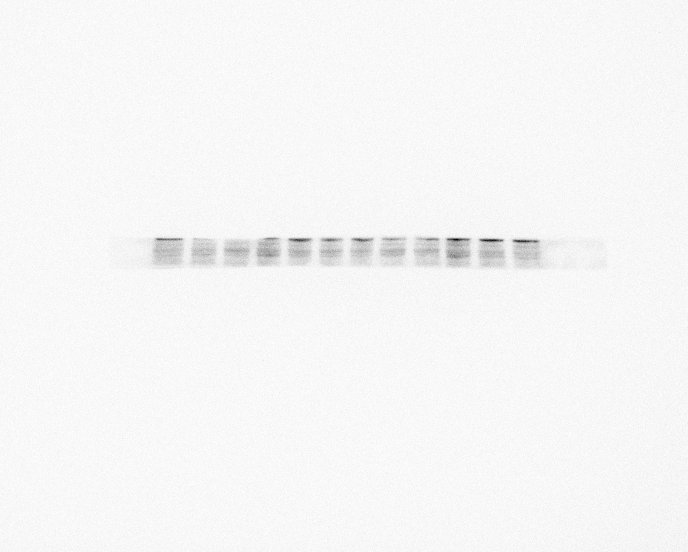

Supplement: Supplementary file 1 [file Data_Sheet_1.ZIP › original WB/SLSP-H9C2 - ╕▒▒╛/pakt/pakt-3.tif]

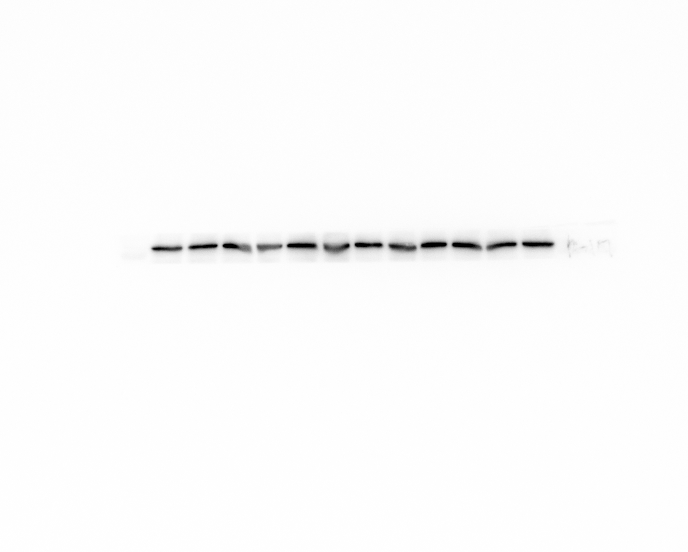

Supplement: Supplementary file 1 [file Data_Sheet_1.ZIP › original WB/SLSP-H9C2 - ╕▒▒╛/pakt/pakt-4.tif]

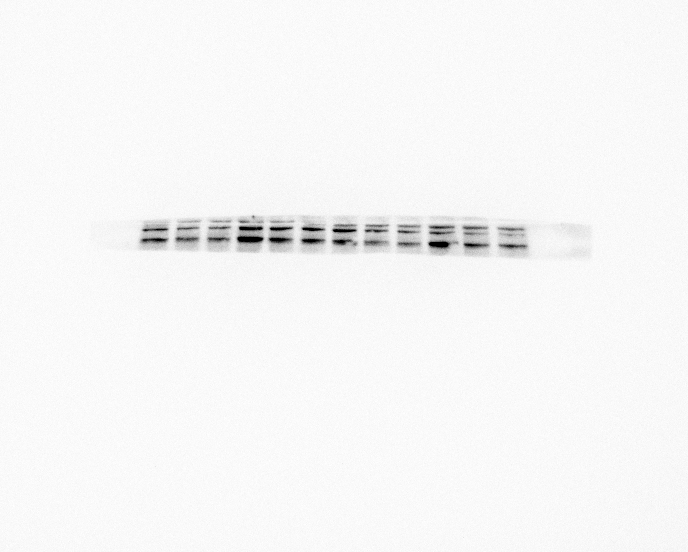

Supplement: Supplementary file 1 [file Data_Sheet_1.ZIP › original WB/SLSP-H9C2 - ╕▒▒╛/pakt/pakt-5.tif]

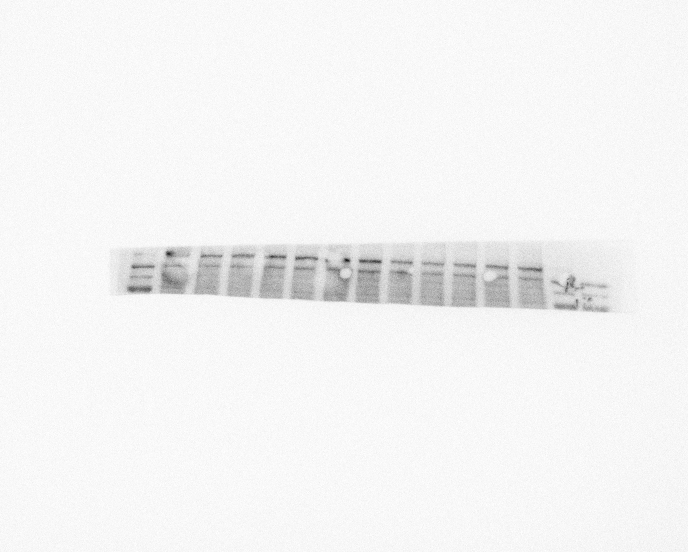

Supplement: Supplementary file 1 [file Data_Sheet_1.ZIP › original WB/SLSP-H9C2 - ╕▒▒╛/pmtor/pm-2-2.tif]

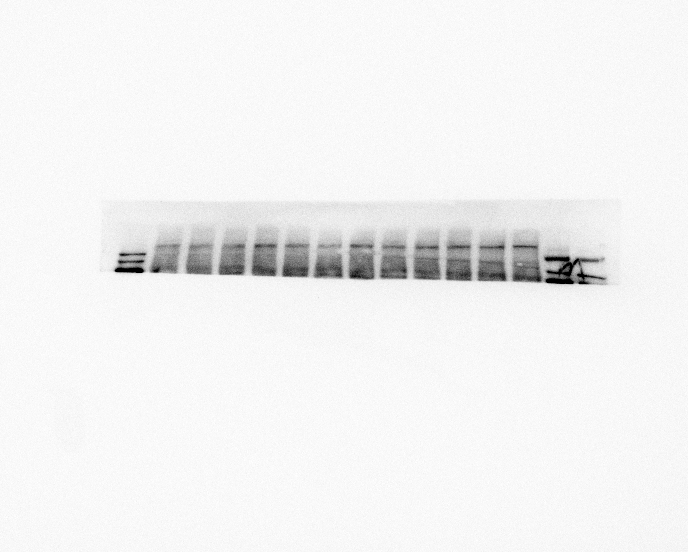

Supplement: Supplementary file 1 [file Data_Sheet_1.ZIP › original WB/SLSP-H9C2 - ╕▒▒╛/pmtor/pm-3.tif]

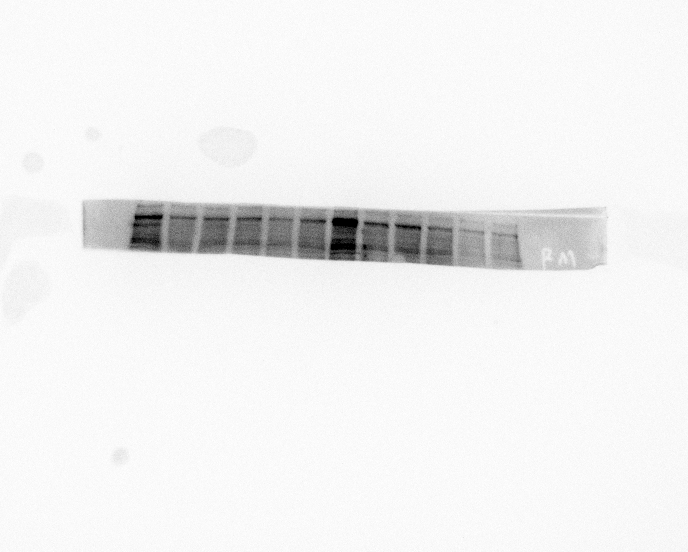

Supplement: Supplementary file 1 [file Data_Sheet_1.ZIP › original WB/SLSP-H9C2 - ╕▒▒╛/pmtor/pm-4-2.tif]

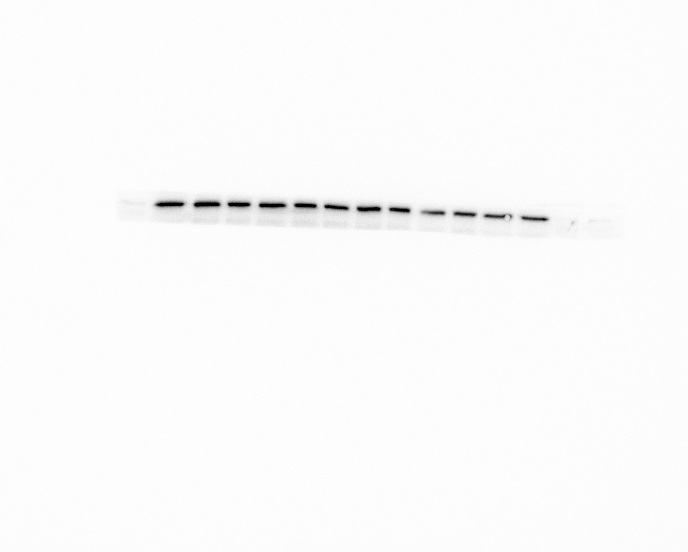

Supplement: Supplementary file 1 [file Data_Sheet_1.ZIP › original WB/SLSP-HEART - ╕▒▒╛/AKT/AKT-1.tif]

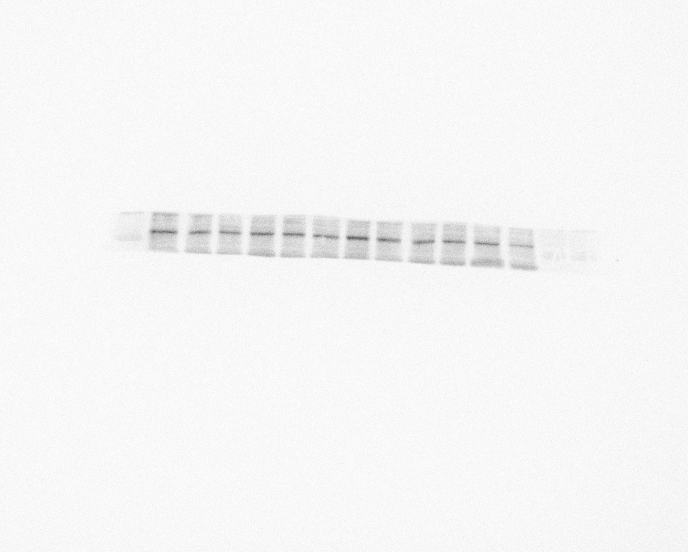

Supplement: Supplementary file 1 [file Data_Sheet_1.ZIP › original WB/SLSP-HEART - ╕▒▒╛/AKT/AKT-2.tif]

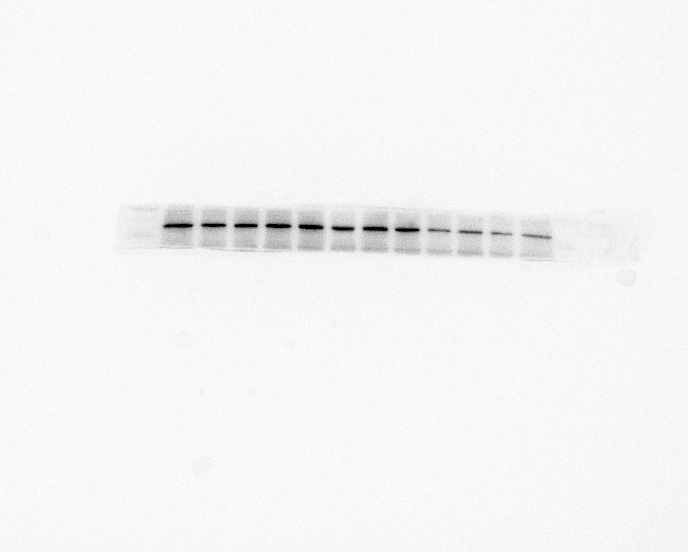

Supplement: Supplementary file 1 [file Data_Sheet_1.ZIP › original WB/SLSP-HEART - ╕▒▒╛/AKT/AKT-3.tif]

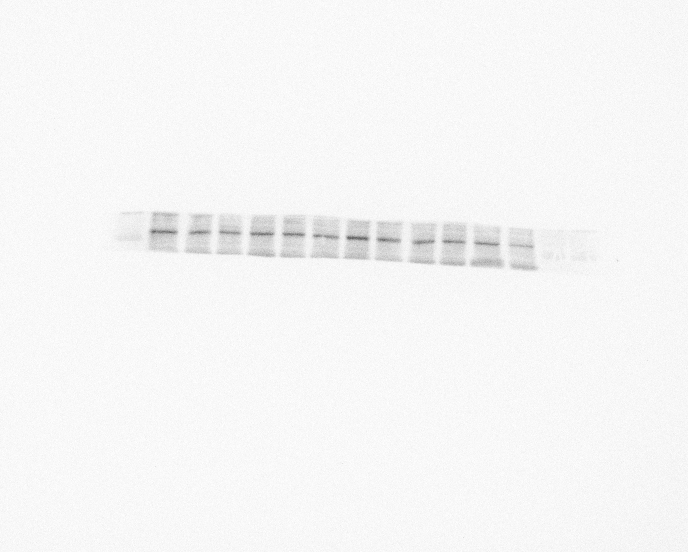

Supplement: Supplementary file 1 [file Data_Sheet_1.ZIP › original WB/SLSP-HEART - ╕▒▒╛/AKT/AKT-4.jpg]

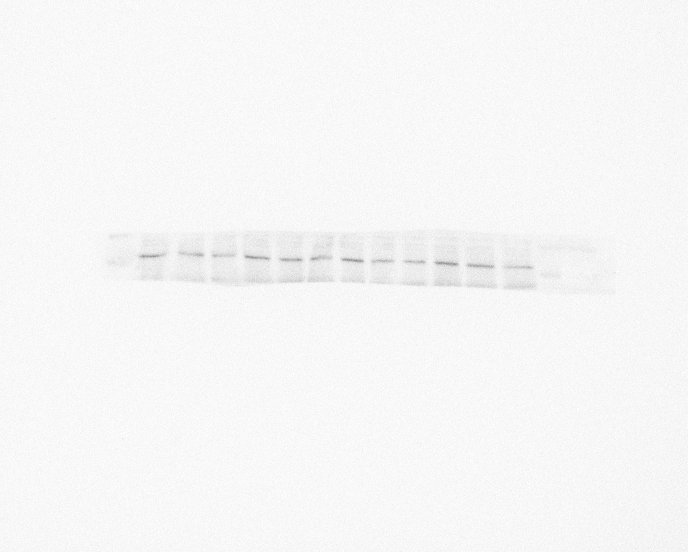

Supplement: Supplementary file 1 [file Data_Sheet_1.ZIP › original WB/SLSP-HEART - ╕▒▒╛/AKT/AKT-5.tif]

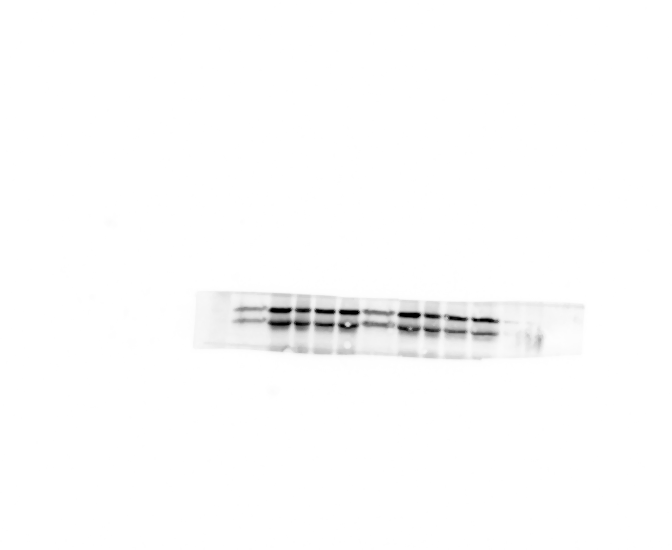

Supplement: Supplementary file 1 [file Data_Sheet_1.ZIP › original WB/SLSP-HEART - ╕▒▒╛/BAX/bax-1.tif]

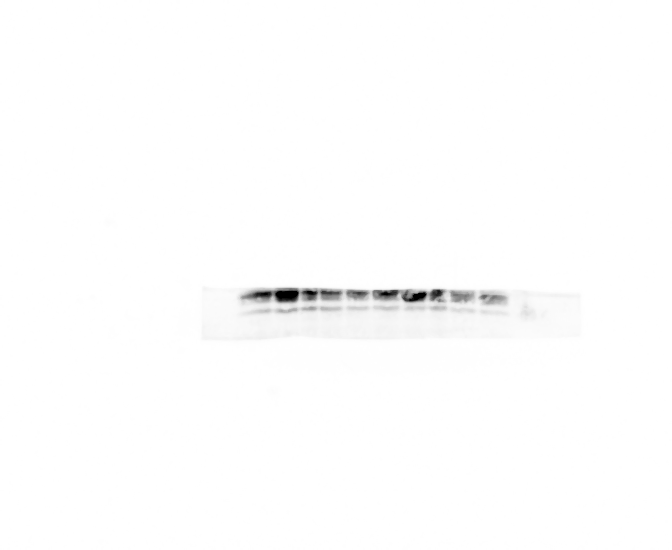

Supplement: Supplementary file 1 [file Data_Sheet_1.ZIP › original WB/SLSP-HEART - ╕▒▒╛/BAX/bax-2.tif]

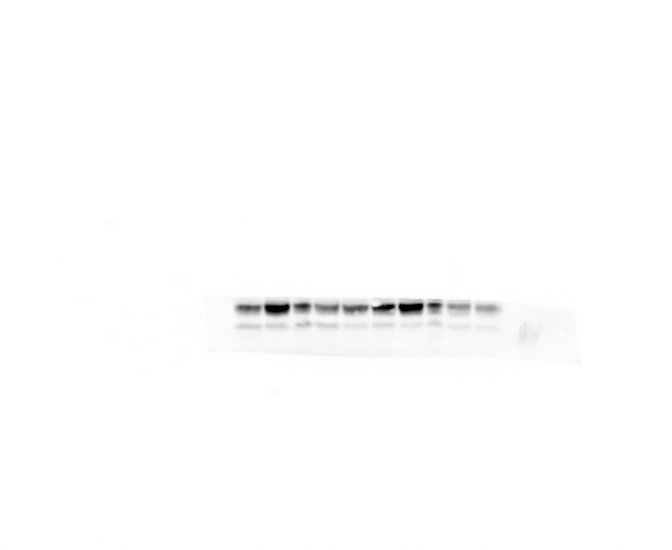

Supplement: Supplementary file 1 [file Data_Sheet_1.ZIP › original WB/SLSP-HEART - ╕▒▒╛/BAX/bax-3.tif]

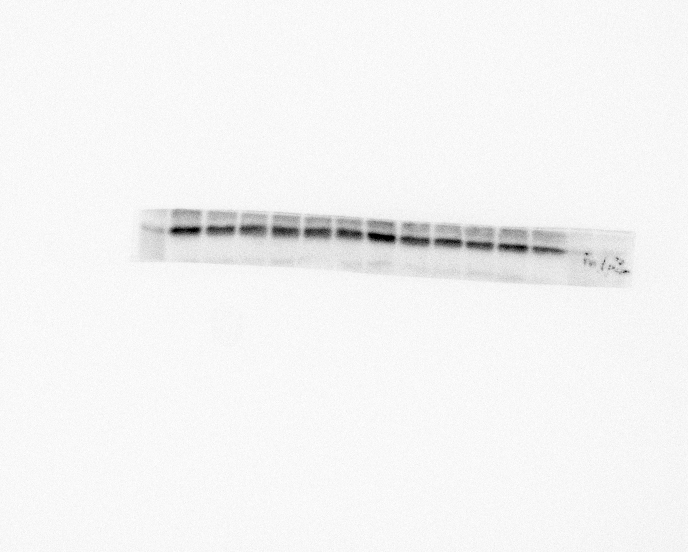

Supplement: Supplementary file 1 [file Data_Sheet_1.ZIP › original WB/SLSP-HEART - ╕▒▒╛/BCL2/BCL2-2.tif]

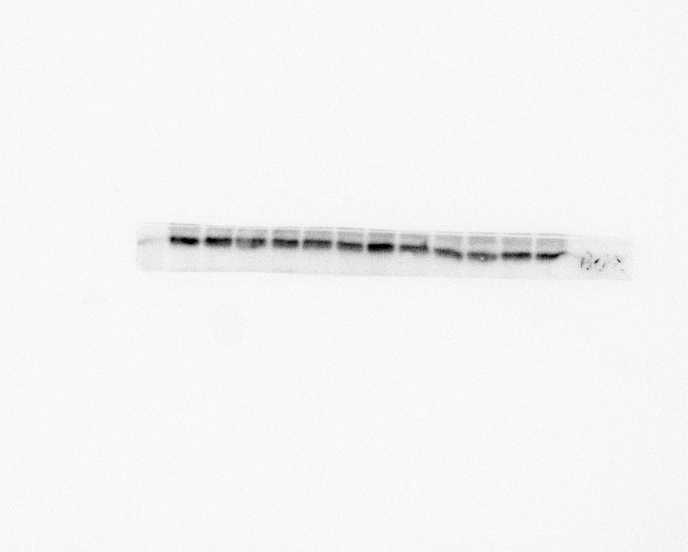

Supplement: Supplementary file 1 [file Data_Sheet_1.ZIP › original WB/SLSP-HEART - ╕▒▒╛/BCL2/BCL2-3.tif]

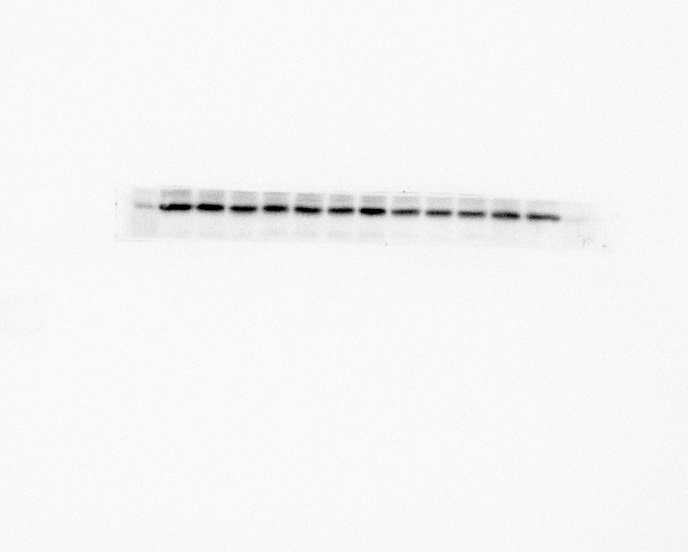

Supplement: Supplementary file 1 [file Data_Sheet_1.ZIP › original WB/SLSP-HEART - ╕▒▒╛/BCL2/BCL2-4.tif]

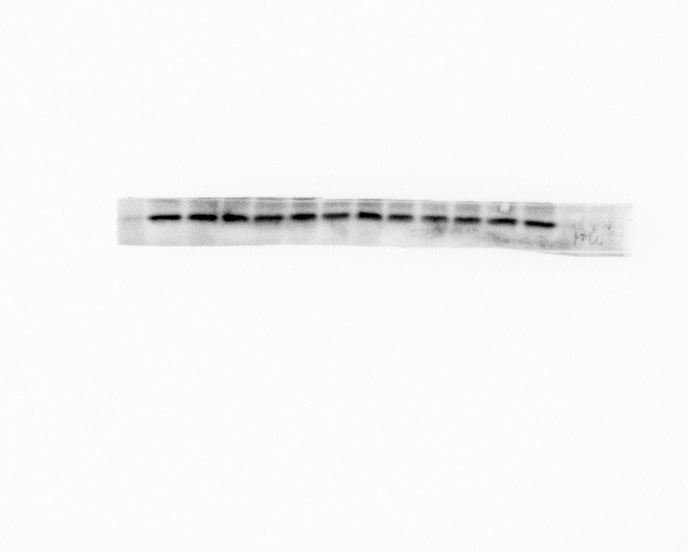

Supplement: Supplementary file 1 [file Data_Sheet_1.ZIP › original WB/SLSP-HEART - ╕▒▒╛/BCL2/BCL2-5.tif]

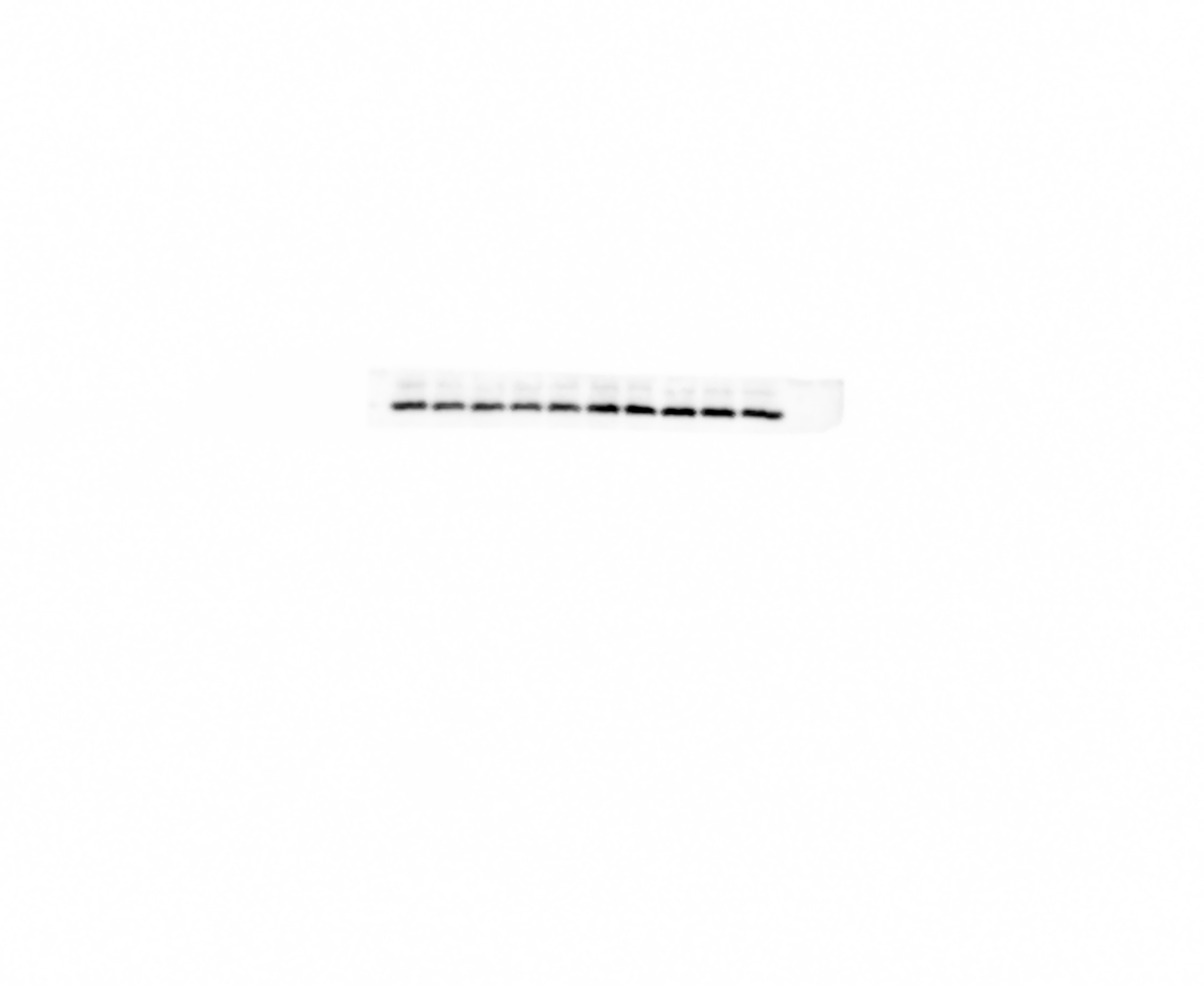

Supplement: Supplementary file 1 [file Data_Sheet_1.ZIP › original WB/SLSP-HEART - ╕▒▒╛/BCL2/bcl2-1.tif]

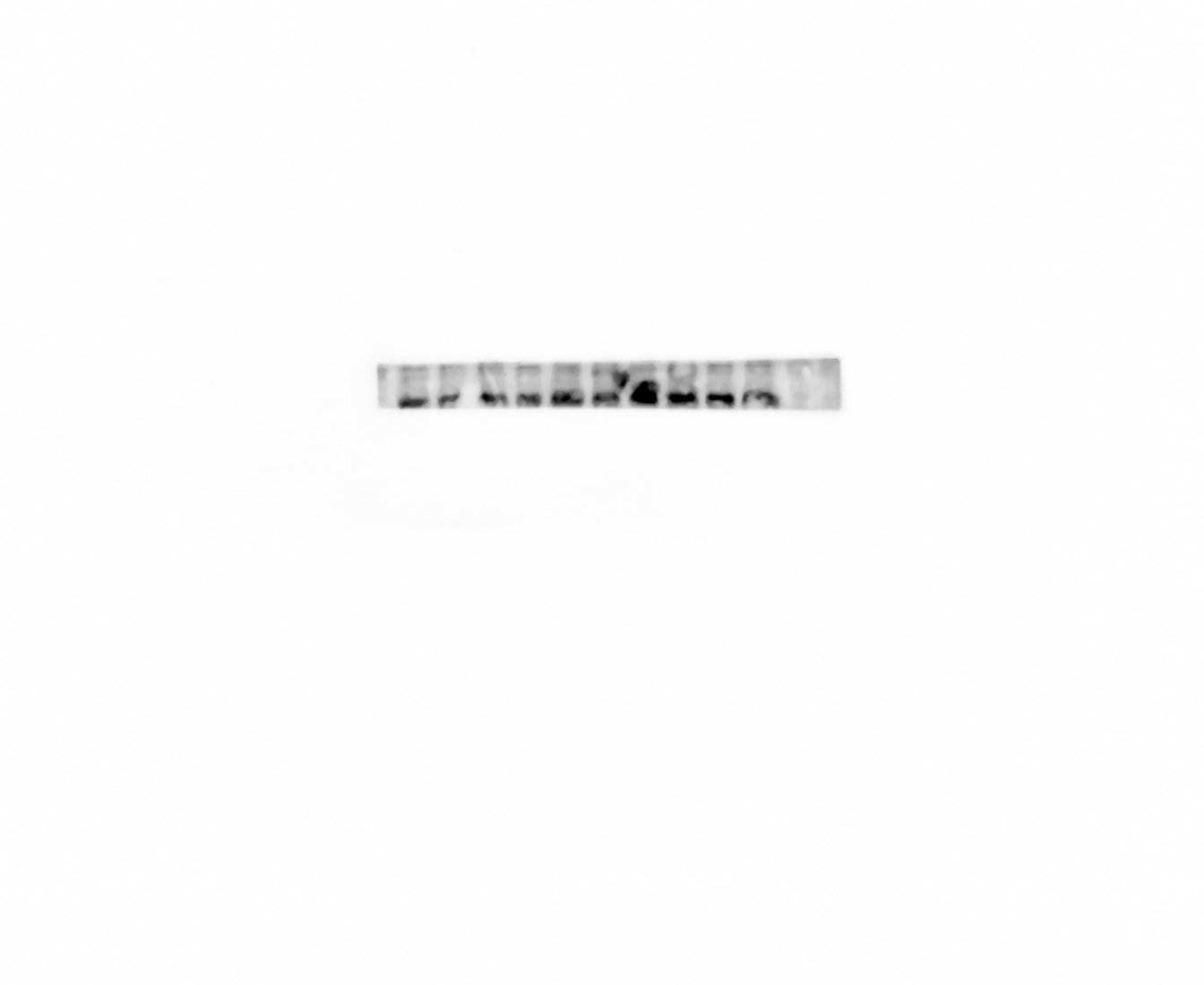

Supplement: Supplementary file 1 [file Data_Sheet_1.ZIP › original WB/SLSP-HEART - ╕▒▒╛/Beclin-1/001-shine[].tif]

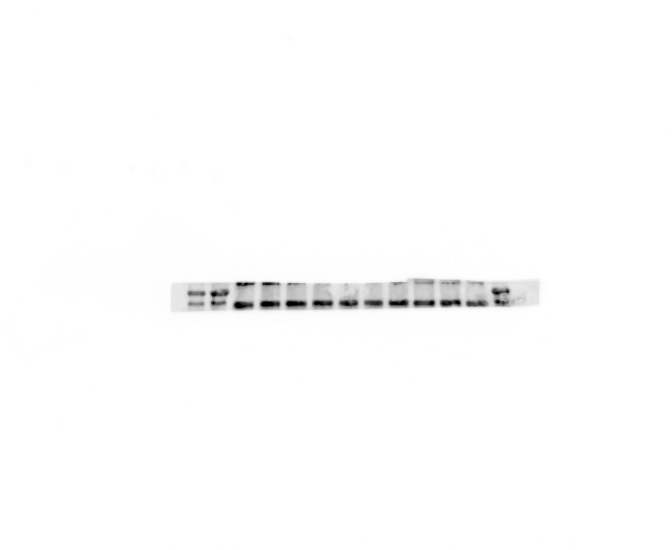

Supplement: Supplementary file 1 [file Data_Sheet_1.ZIP › original WB/SLSP-HEART - ╕▒▒╛/Beclin-1/beclin-1.tif]

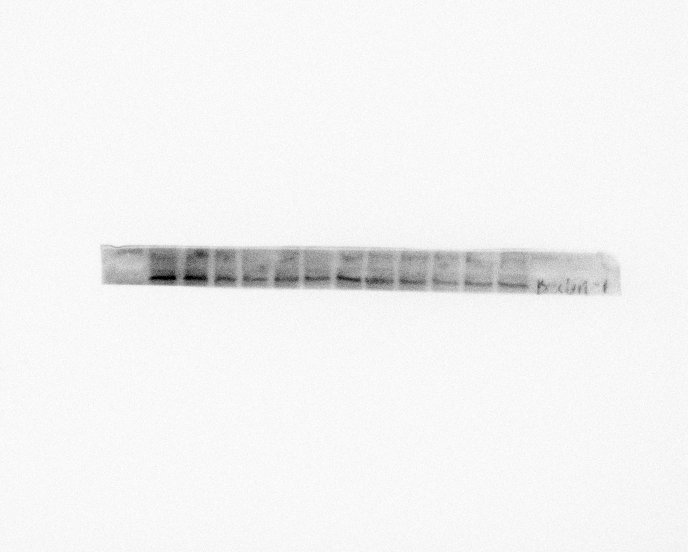

Supplement: Supplementary file 1 [file Data_Sheet_1.ZIP › original WB/SLSP-HEART - ╕▒▒╛/Beclin-1/beclin-2.tif]

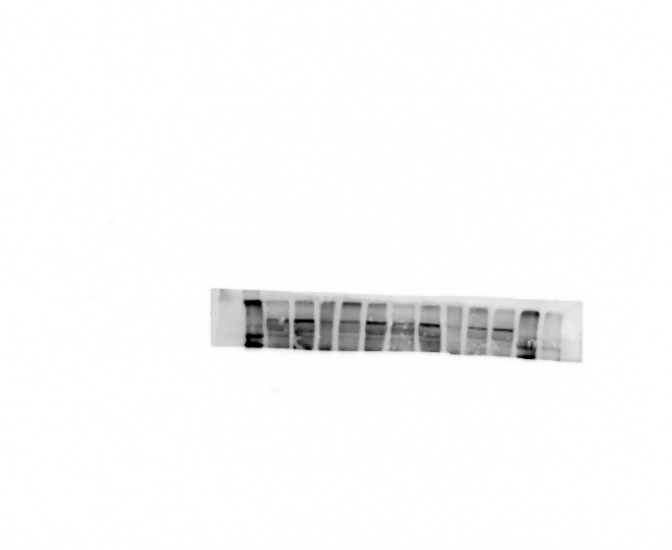

Supplement: Supplementary file 1 [file Data_Sheet_1.ZIP › original WB/SLSP-HEART - ╕▒▒╛/Beclin-1/beclin-3.tif]

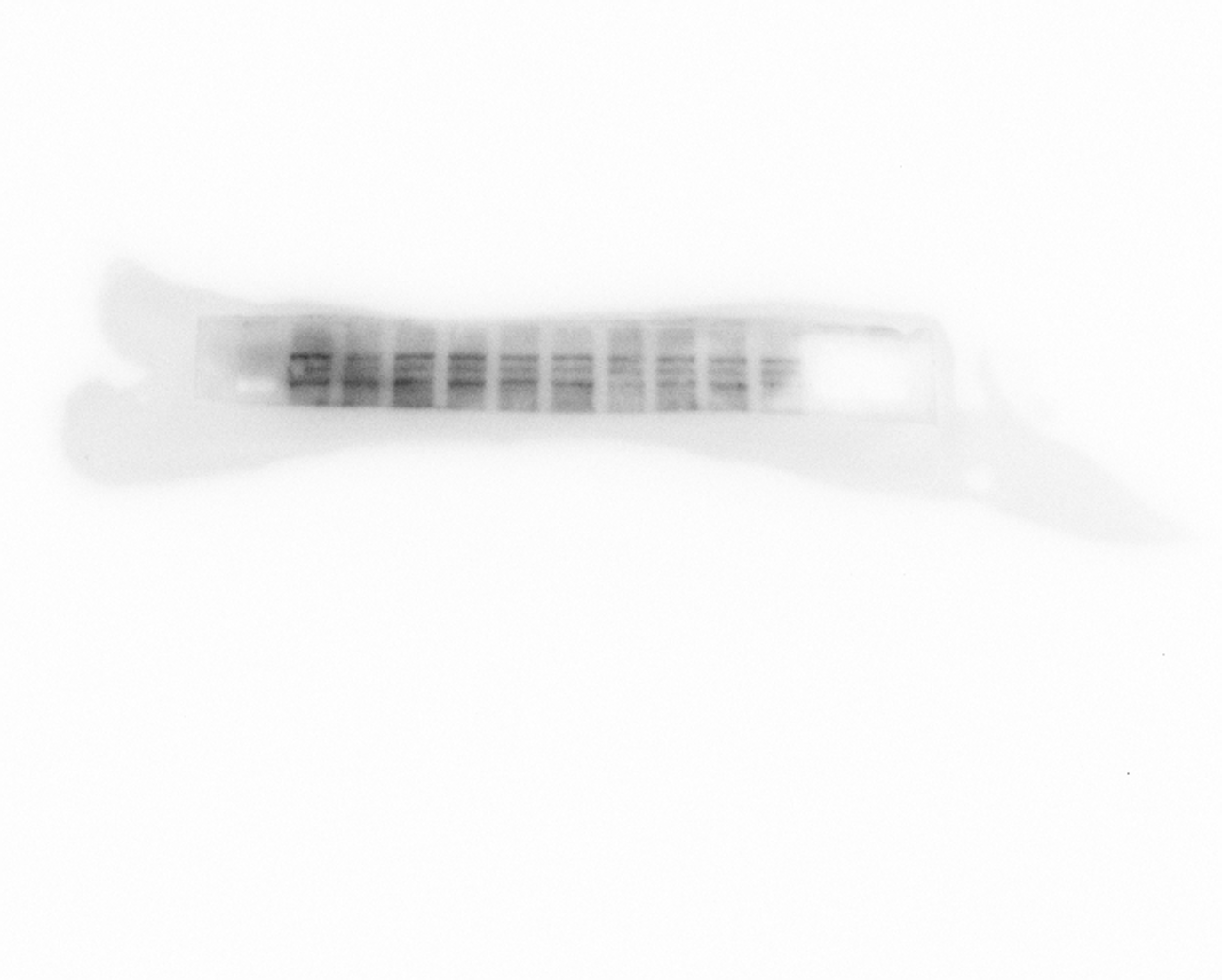

Supplement: Supplementary file 1 [file Data_Sheet_1.ZIP › original WB/SLSP-HEART - ╕▒▒╛/P-PI3K/PPI3K-1.Tif]

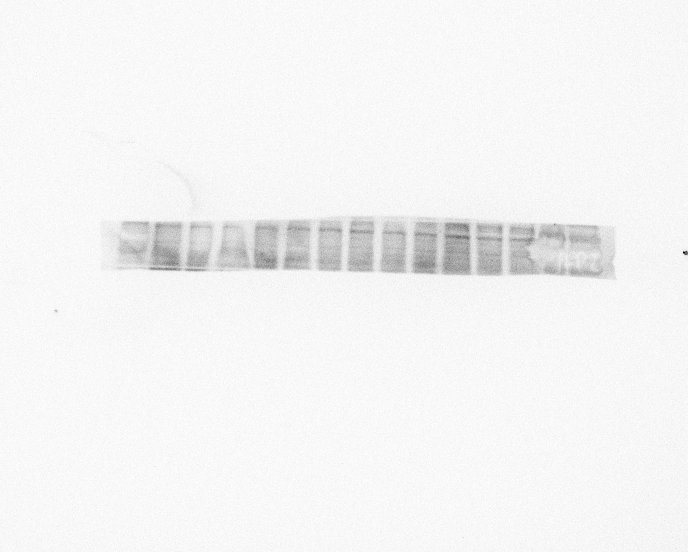

Supplement: Supplementary file 1 [file Data_Sheet_1.ZIP › original WB/SLSP-HEART - ╕▒▒╛/P-PI3K/PPI3K-2.tif]

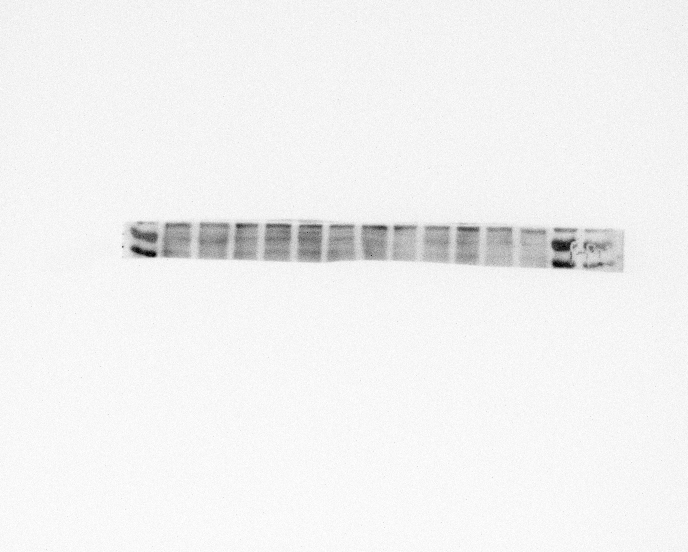

Supplement: Supplementary file 1 [file Data_Sheet_1.ZIP › original WB/SLSP-HEART - ╕▒▒╛/P-PI3K/PPI3K-3.tif]

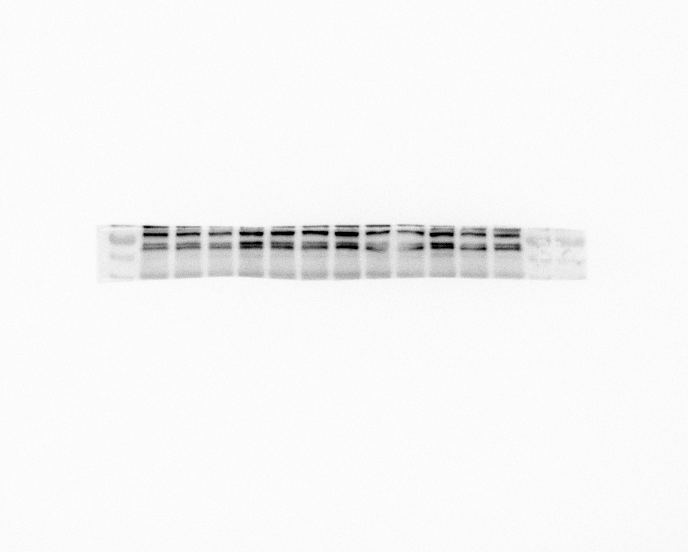

Supplement: Supplementary file 1 [file Data_Sheet_1.ZIP › original WB/SLSP-HEART - ╕▒▒╛/P-PI3K/PPI3K-4.tif]

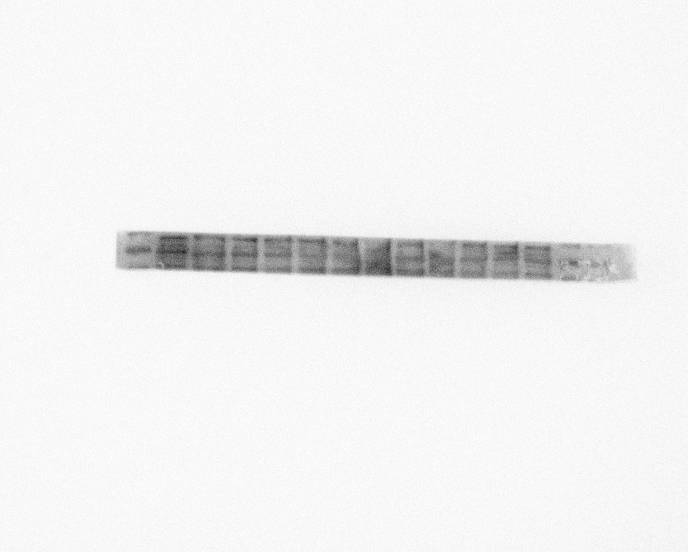

Supplement: Supplementary file 1 [file Data_Sheet_1.ZIP › original WB/SLSP-HEART - ╕▒▒╛/P-PI3K/PPI3K-5.tif]

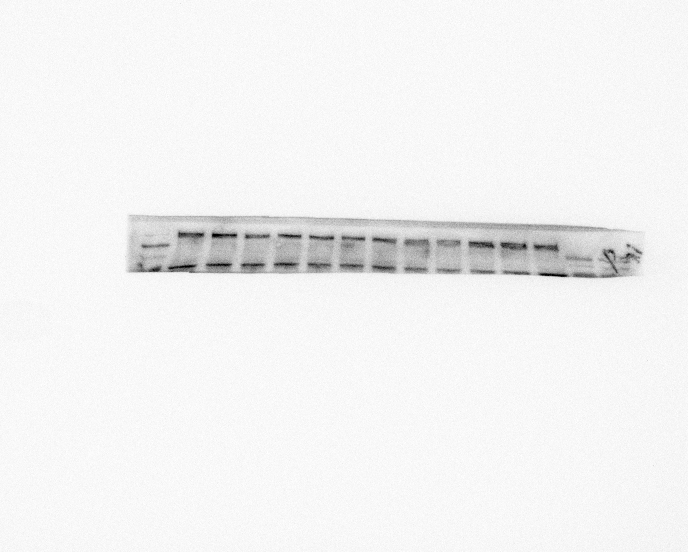

Supplement: Supplementary file 1 [file Data_Sheet_1.ZIP › original WB/SLSP-HEART - ╕▒▒╛/P-mTOR/PM-1-2.tif]

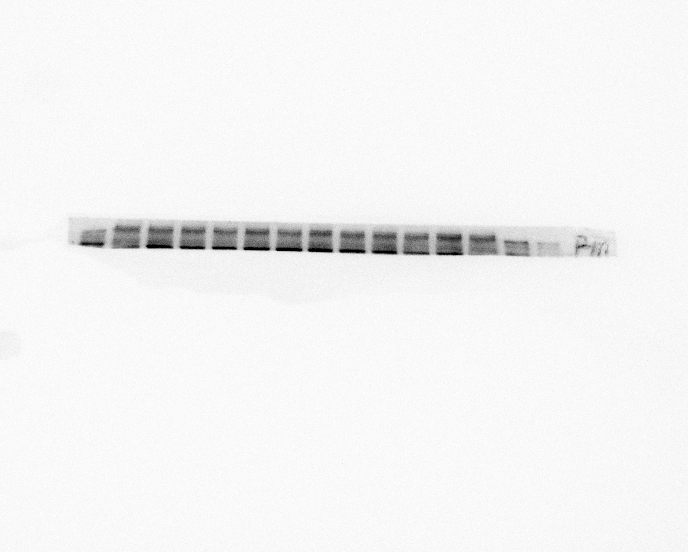

Supplement: Supplementary file 1 [file Data_Sheet_1.ZIP › original WB/SLSP-HEART - ╕▒▒╛/P-mTOR/PM-2-1.tif]

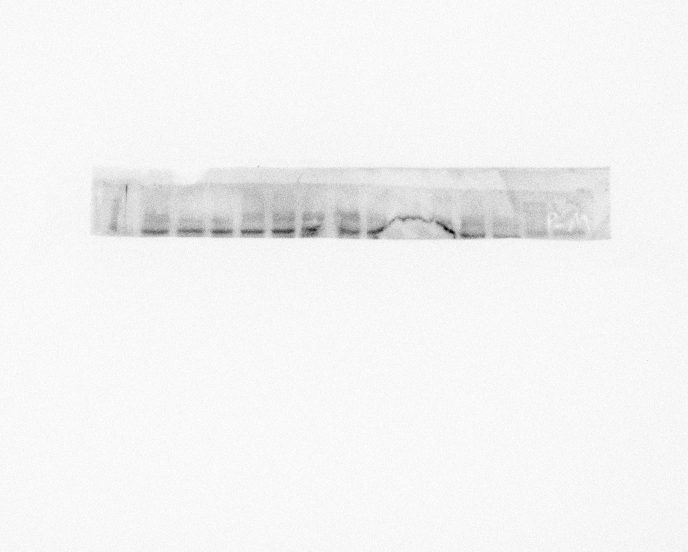

Supplement: Supplementary file 1 [file Data_Sheet_1.ZIP › original WB/SLSP-HEART - ╕▒▒╛/P-mTOR/PM-2-2.tif]

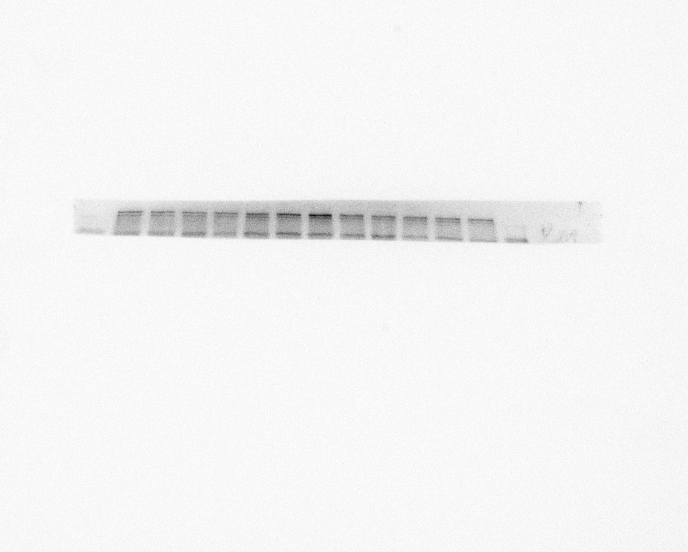

Supplement: Supplementary file 1 [file Data_Sheet_1.ZIP › original WB/SLSP-HEART - ╕▒▒╛/P-mTOR/PM-4-2.tif]

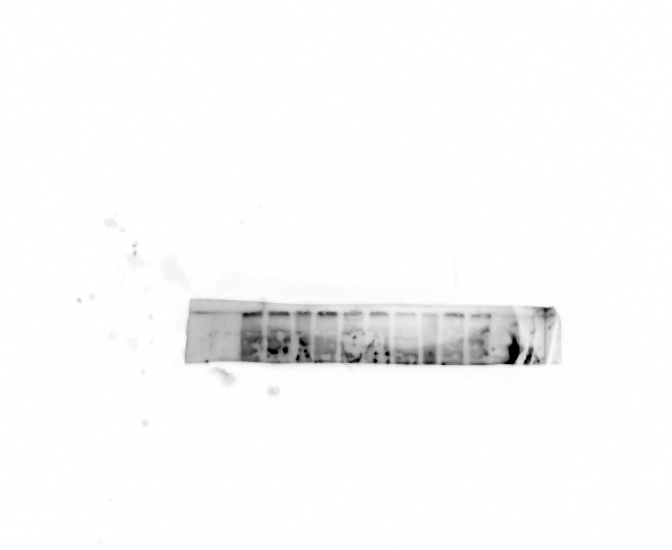

Supplement: Supplementary file 1 [file Data_Sheet_1.ZIP › original WB/SLSP-HEART - ╕▒▒╛/P-mTOR/PM-5.tif]

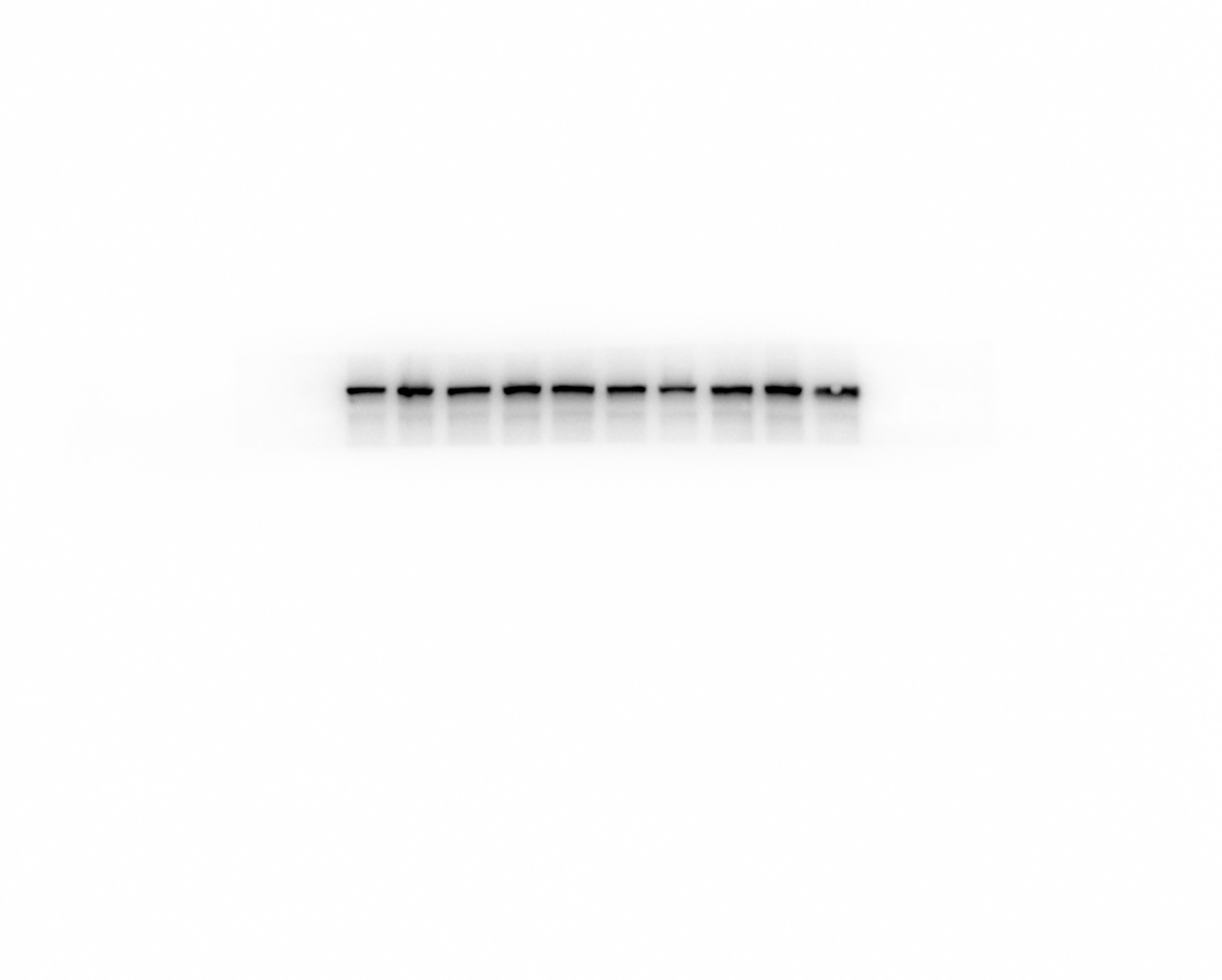

Supplement: Supplementary file 1 [file Data_Sheet_1.ZIP › original WB/SLSP-HEART - ╕▒▒╛/PI3K/PI3K-1.Tif]

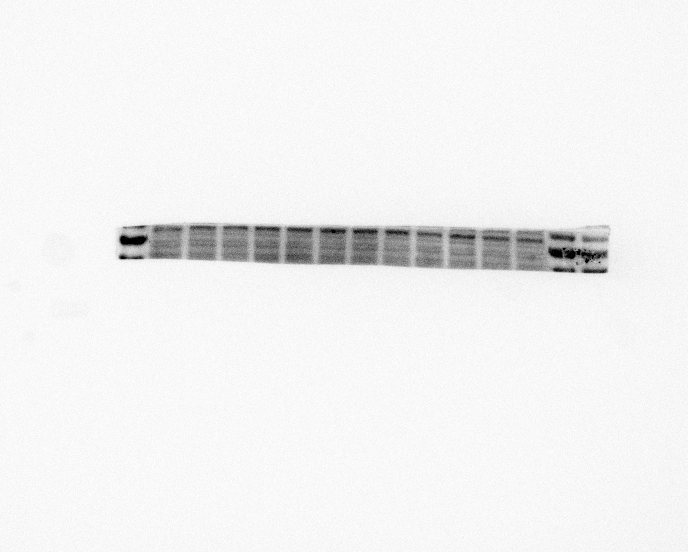

Supplement: Supplementary file 1 [file Data_Sheet_1.ZIP › original WB/SLSP-HEART - ╕▒▒╛/PI3K/PI3K-2.tif]

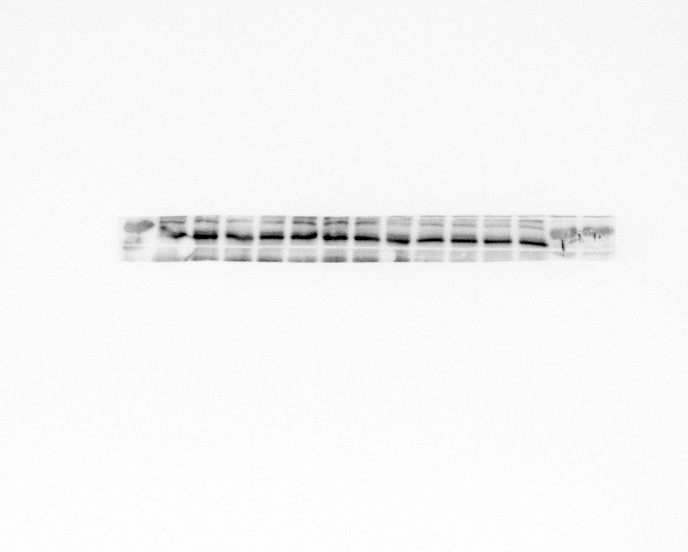

Supplement: Supplementary file 1 [file Data_Sheet_1.ZIP › original WB/SLSP-HEART - ╕▒▒╛/PI3K/PI3K-3.tif]

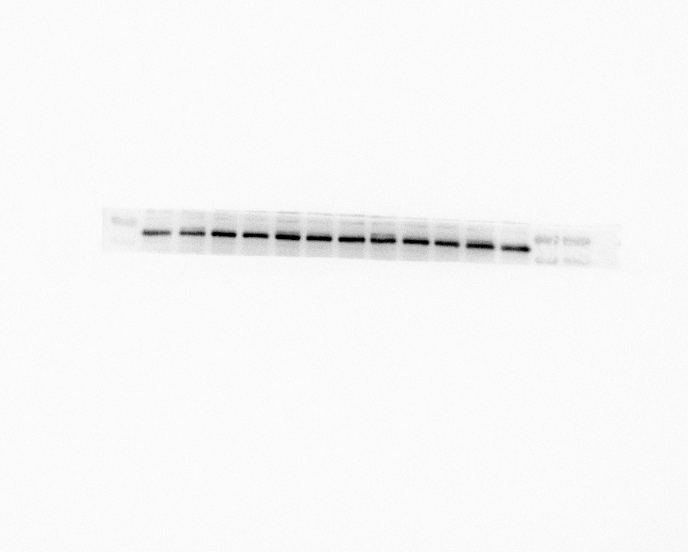

Supplement: Supplementary file 1 [file Data_Sheet_1.ZIP › original WB/SLSP-HEART - ╕▒▒╛/PI3K/PI3K-4.tif]

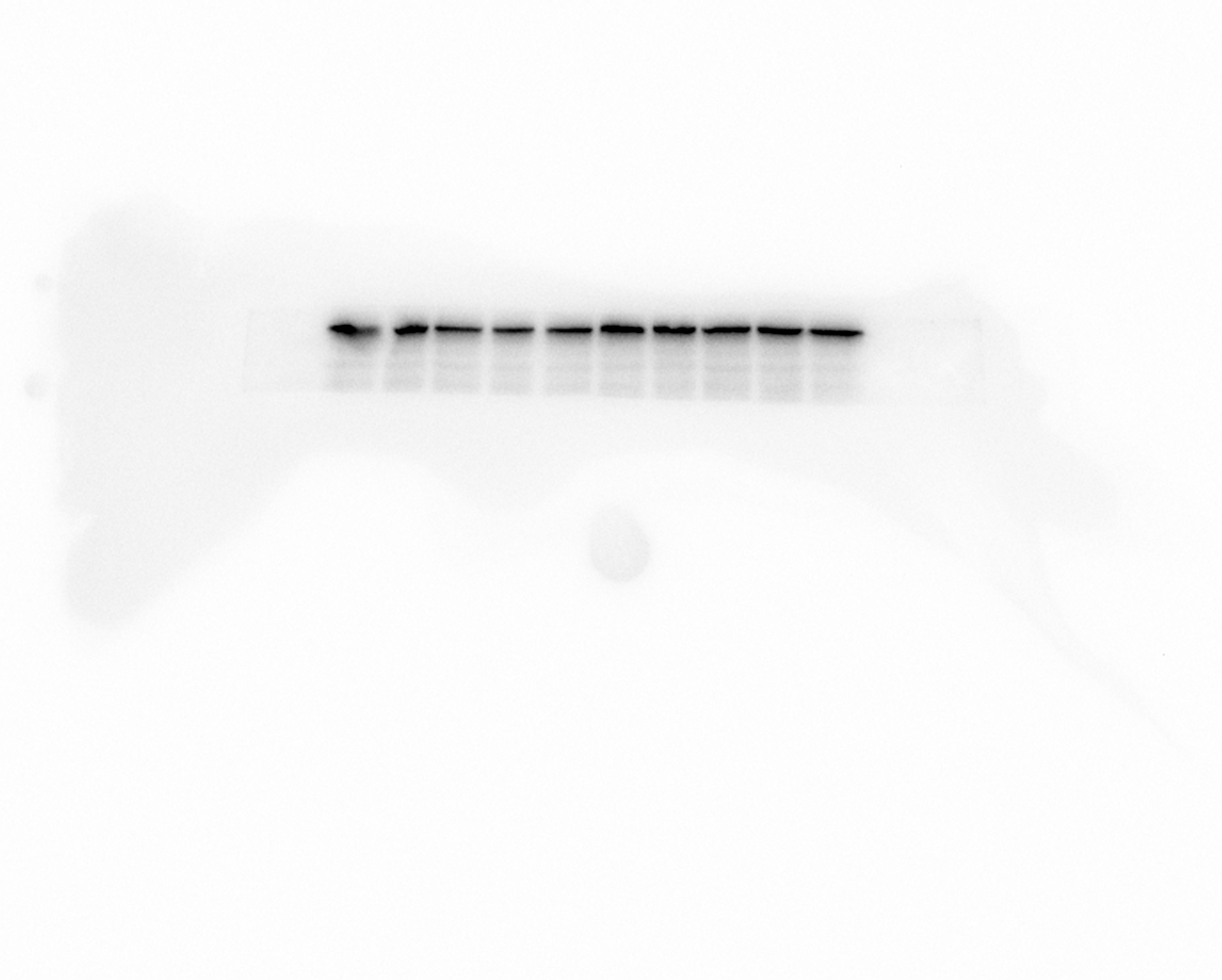

Supplement: Supplementary file 1 [file Data_Sheet_1.ZIP › original WB/SLSP-HEART - ╕▒▒╛/actin/ACTIN-1.Tif]

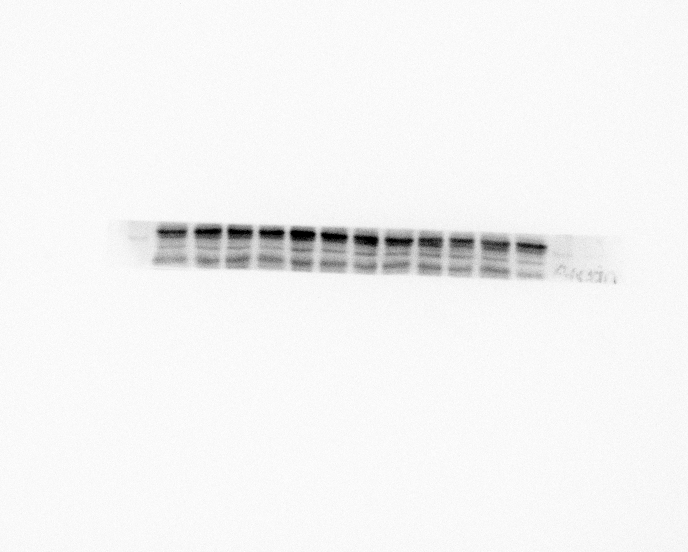

Supplement: Supplementary file 1 [file Data_Sheet_1.ZIP › original WB/SLSP-HEART - ╕▒▒╛/actin/ACTIN-2.tif]

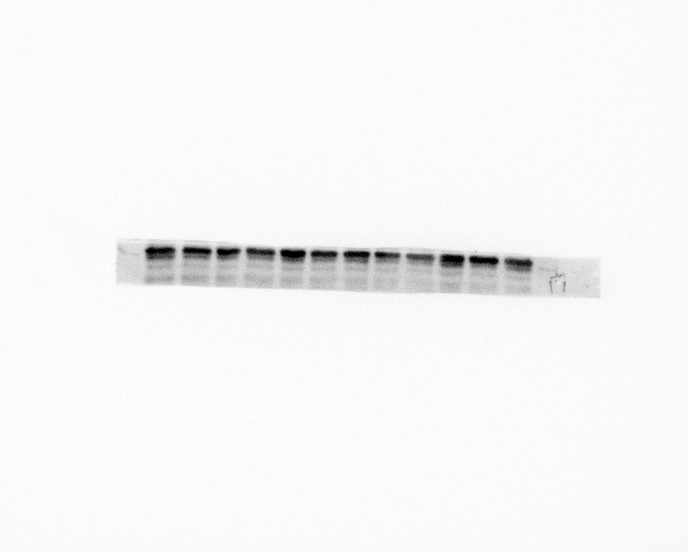

Supplement: Supplementary file 1 [file Data_Sheet_1.ZIP › original WB/SLSP-HEART - ╕▒▒╛/actin/ACTIN-3.tif]

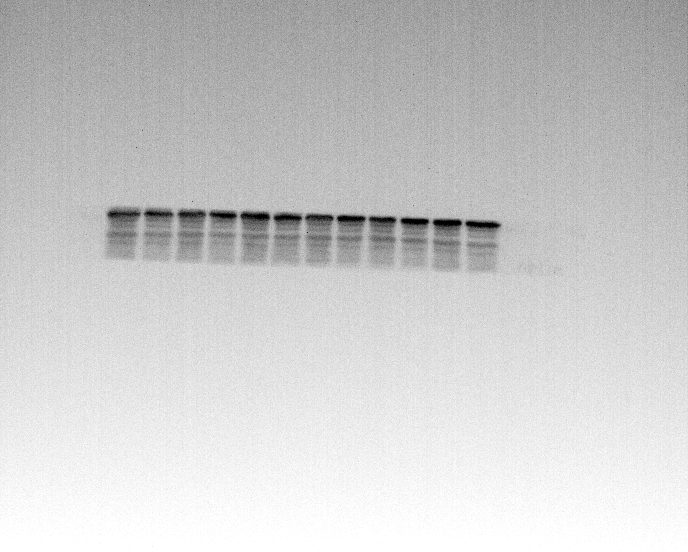

Supplement: Supplementary file 1 [file Data_Sheet_1.ZIP › original WB/SLSP-HEART - ╕▒▒╛/actin/ACTIN-4.tif]

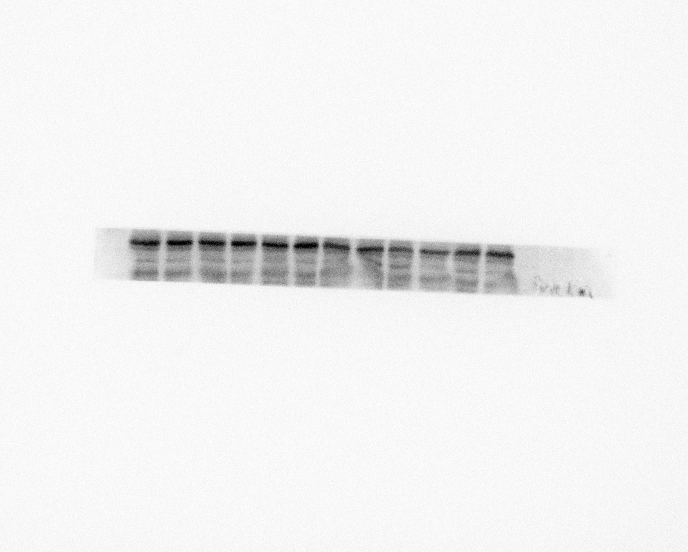

Supplement: Supplementary file 1 [file Data_Sheet_1.ZIP › original WB/SLSP-HEART - ╕▒▒╛/actin/ACTIN-5.tif]

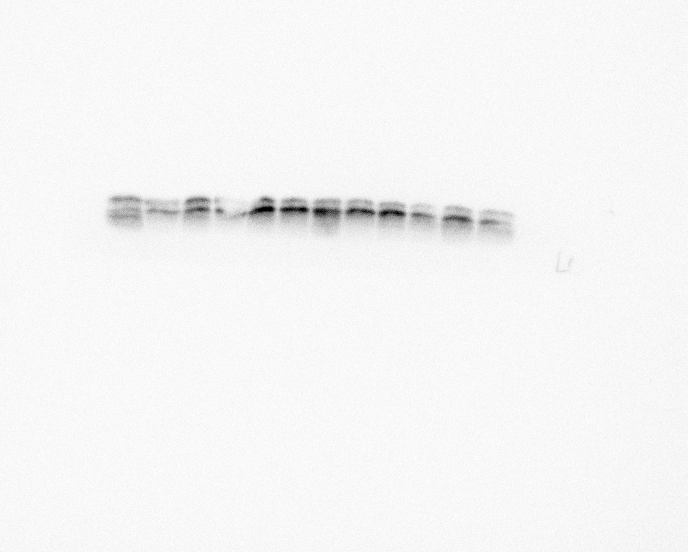

Supplement: Supplementary file 1 [file Data_Sheet_1.ZIP › original WB/SLSP-HEART - ╕▒▒╛/lc/LC-1-2.tif]

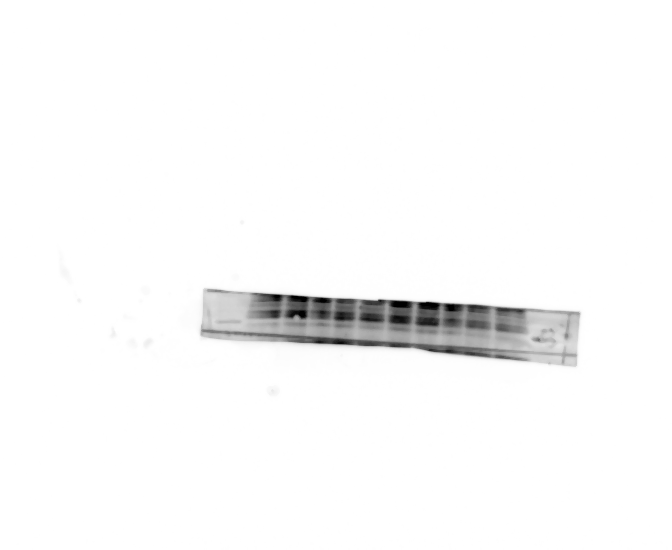

Supplement: Supplementary file 1 [file Data_Sheet_1.ZIP › original WB/SLSP-HEART - ╕▒▒╛/lc/LC-4.tif]

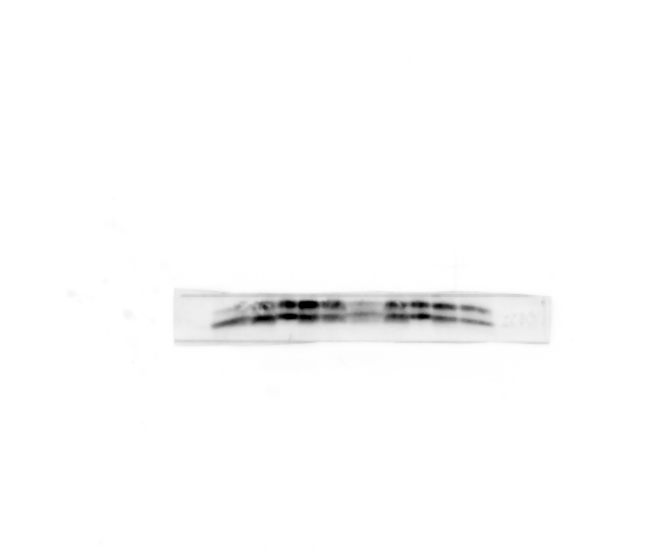

Supplement: Supplementary file 1 [file Data_Sheet_1.ZIP › original WB/SLSP-HEART - ╕▒▒╛/lc/LC-5.tif]

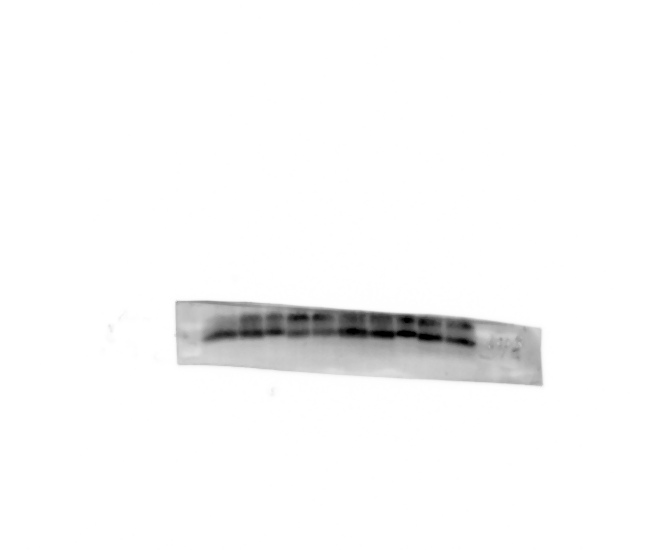

Supplement: Supplementary file 1 [file Data_Sheet_1.ZIP › original WB/SLSP-HEART - ╕▒▒╛/lc/lc-1.tif]

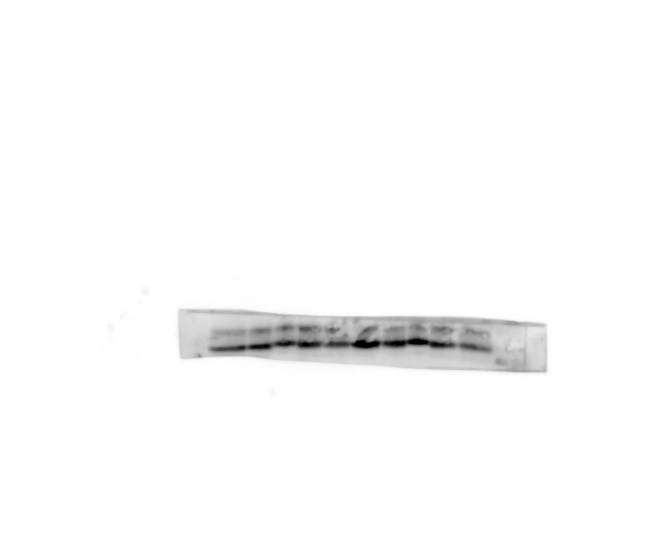

Supplement: Supplementary file 1 [file Data_Sheet_1.ZIP › original WB/SLSP-HEART - ╕▒▒╛/lc/lc-3.tif]

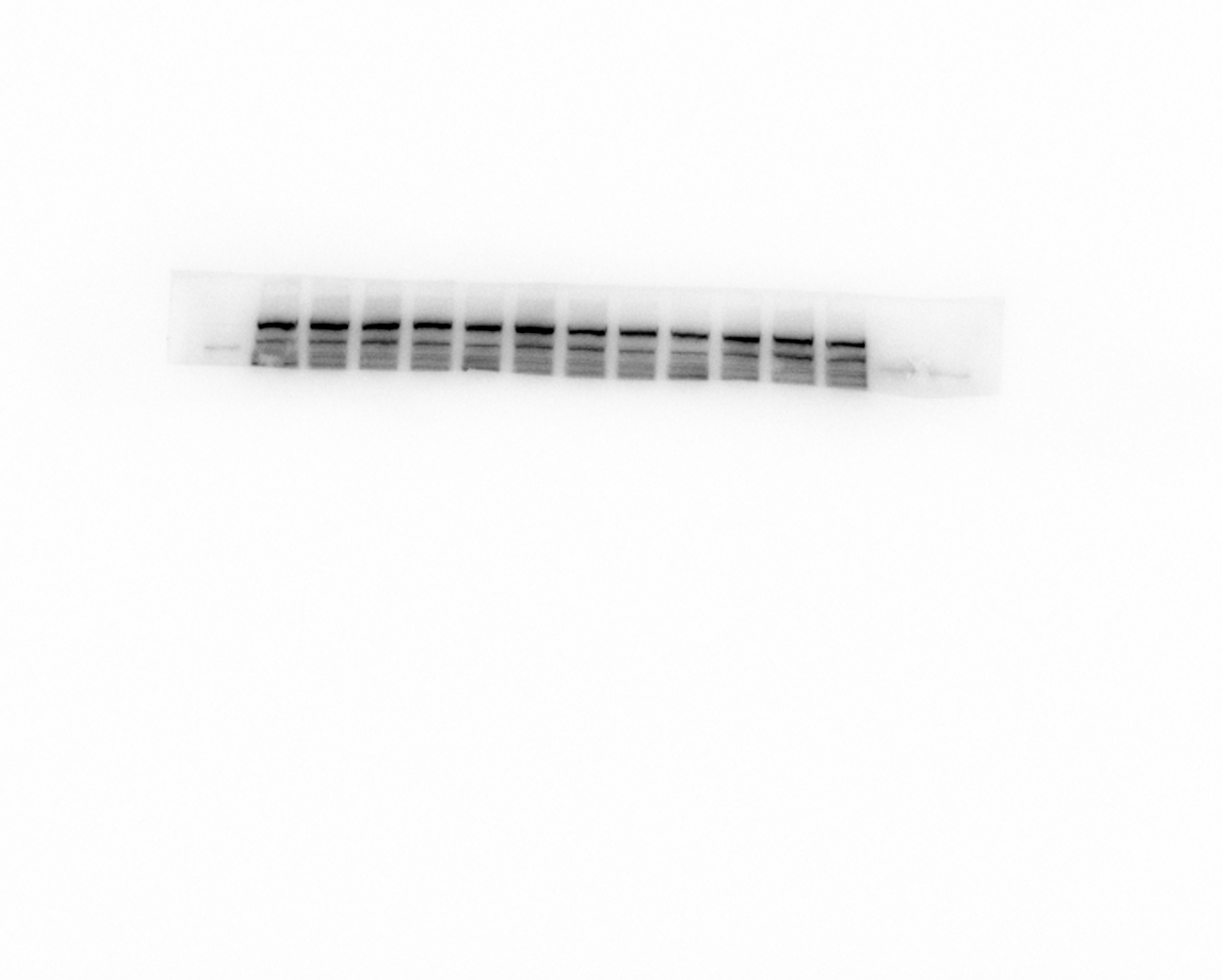

Supplement: Supplementary file 1 [file Data_Sheet_1.ZIP › original WB/SLSP-HEART - ╕▒▒╛/mtor/M1-1.Tif]

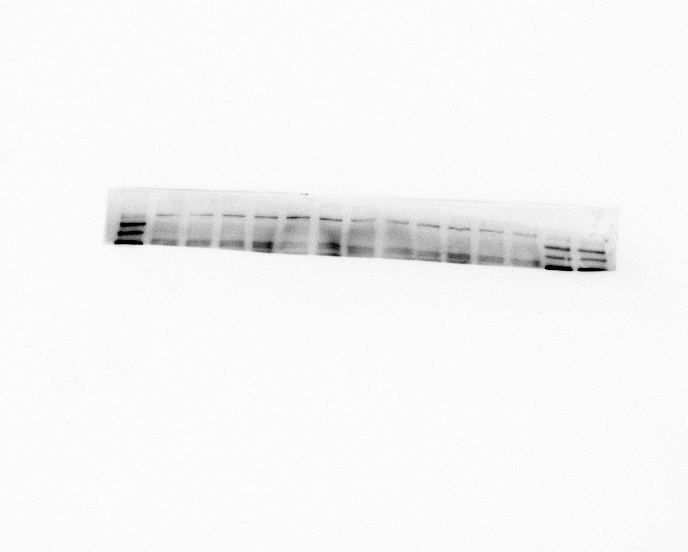

Supplement: Supplementary file 1 [file Data_Sheet_1.ZIP › original WB/SLSP-HEART - ╕▒▒╛/mtor/M1-2.tif]

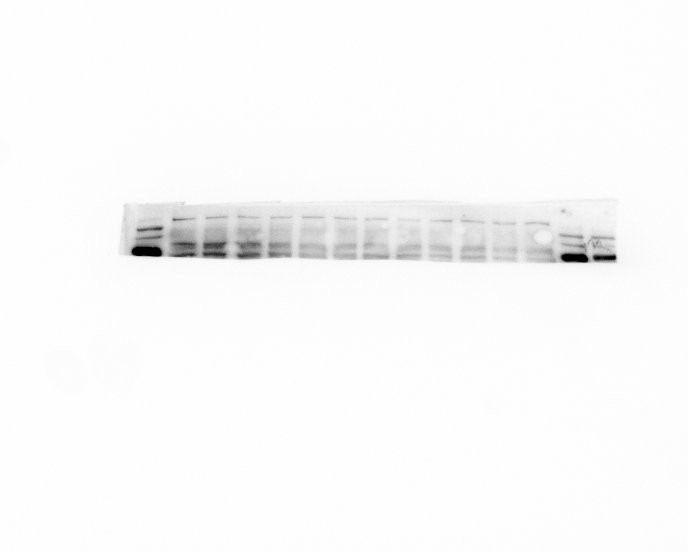

Supplement: Supplementary file 1 [file Data_Sheet_1.ZIP › original WB/SLSP-HEART - ╕▒▒╛/mtor/M2-2.tif]

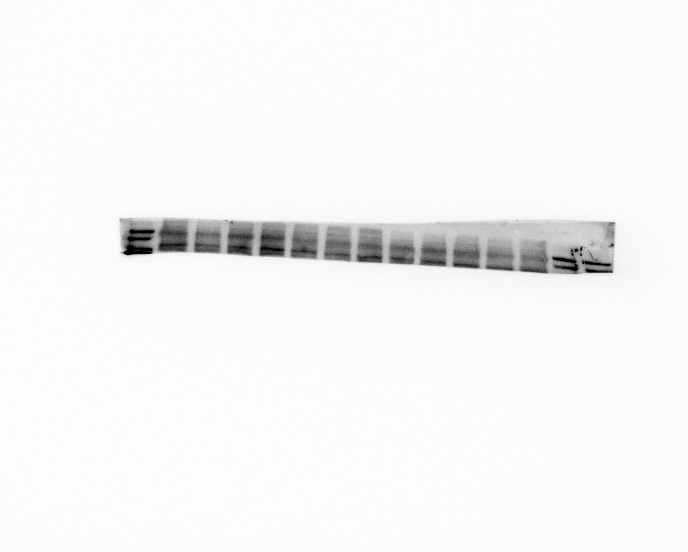

Supplement: Supplementary file 1 [file Data_Sheet_1.ZIP › original WB/SLSP-HEART - ╕▒▒╛/mtor/M2.tif]

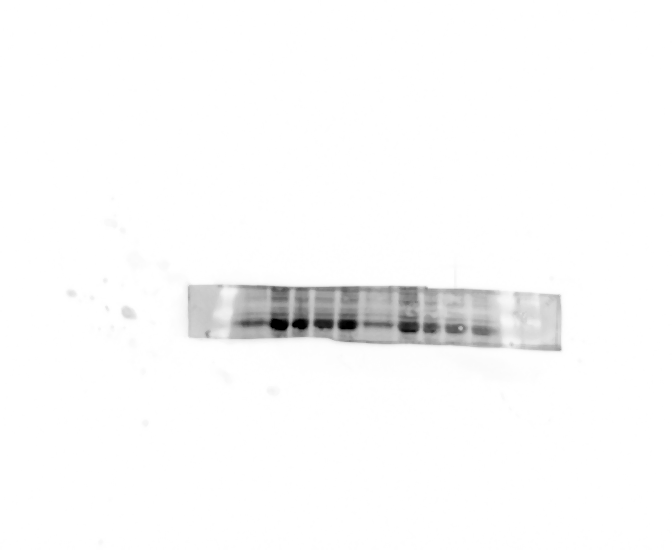

Supplement: Supplementary file 1 [file Data_Sheet_1.ZIP › original WB/SLSP-HEART - ╕▒▒╛/p62/P62-1.tif]

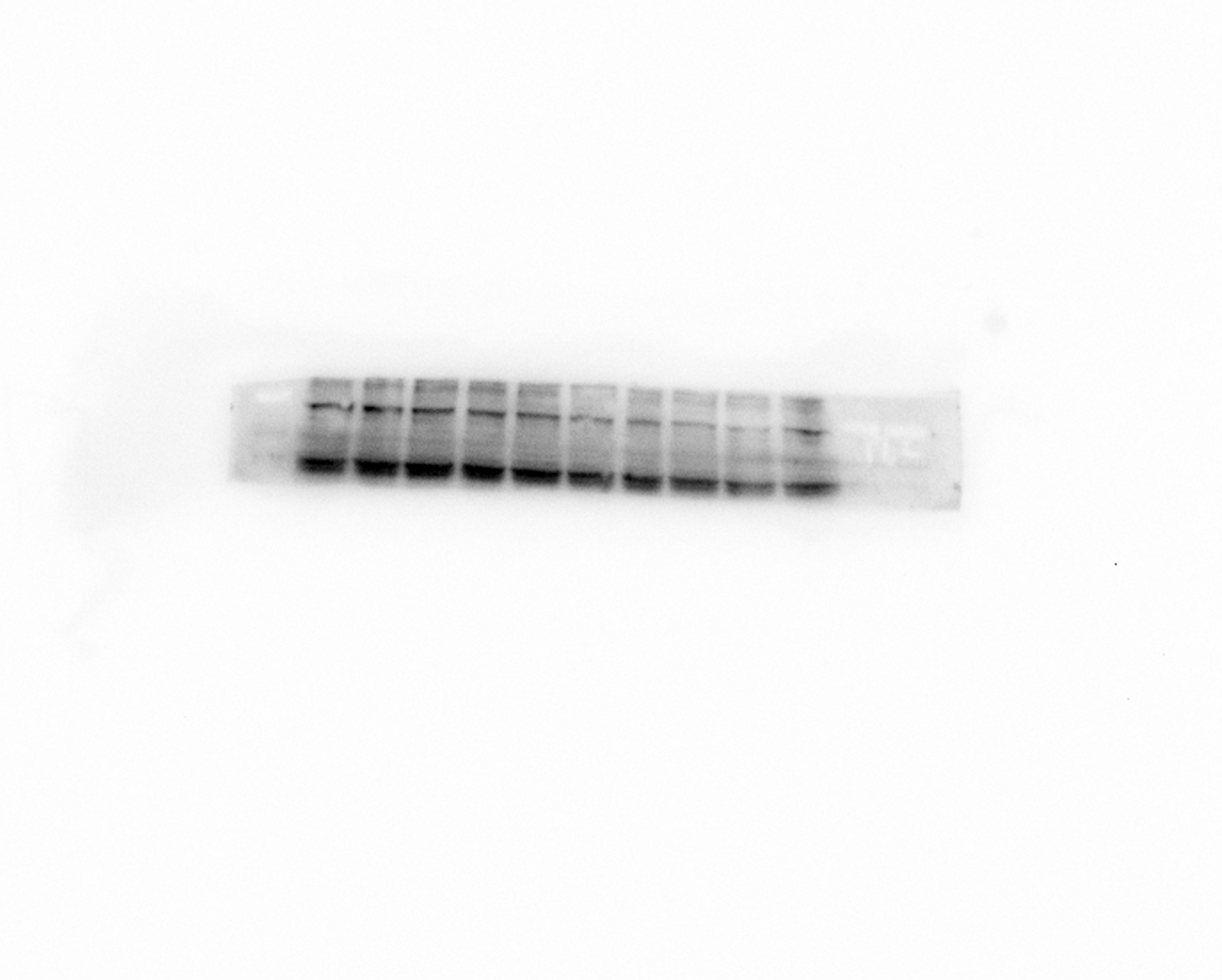

Supplement: Supplementary file 1 [file Data_Sheet_1.ZIP › original WB/SLSP-HEART - ╕▒▒╛/p62/P62-2-3.jpg]

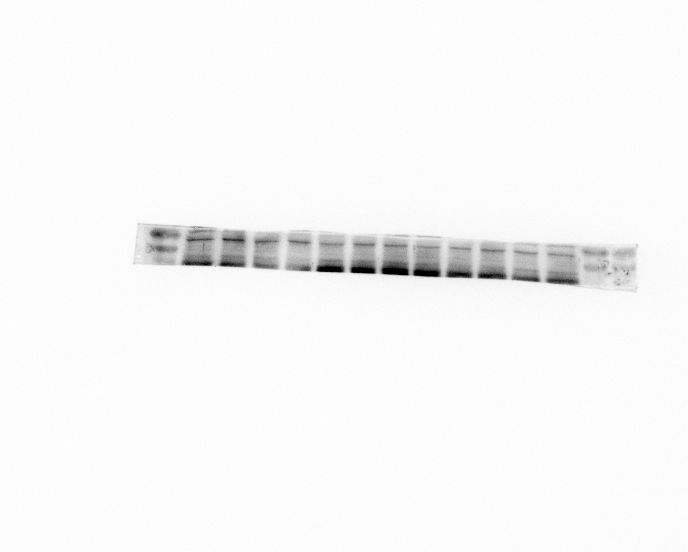

Supplement: Supplementary file 1 [file Data_Sheet_1.ZIP › original WB/SLSP-HEART - ╕▒▒╛/p62/P62-2.tif]

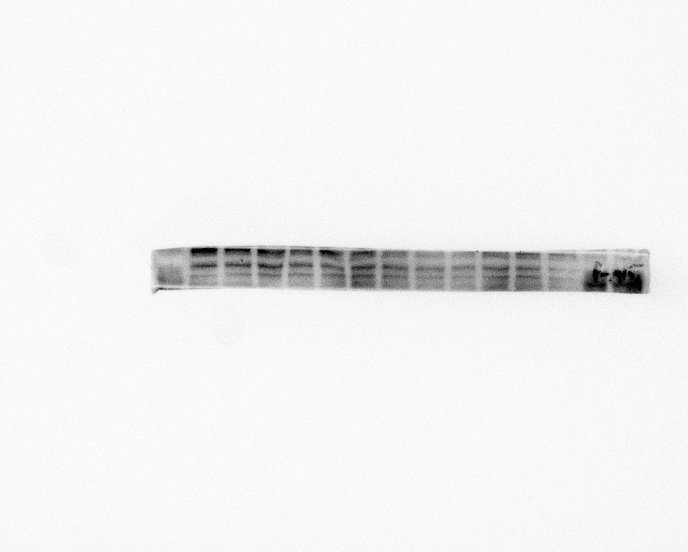

Supplement: Supplementary file 1 [file Data_Sheet_1.ZIP › original WB/SLSP-HEART - ╕▒▒╛/pakt/PAKT-1.tif]

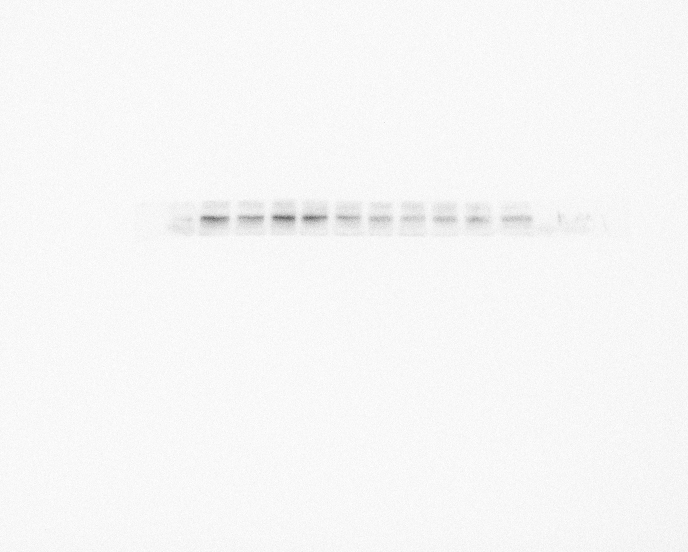

Supplement: Supplementary file 1 [file Data_Sheet_1.ZIP › original WB/SLSP-HEART - ╕▒▒╛/pakt/PAKT-2.tif]

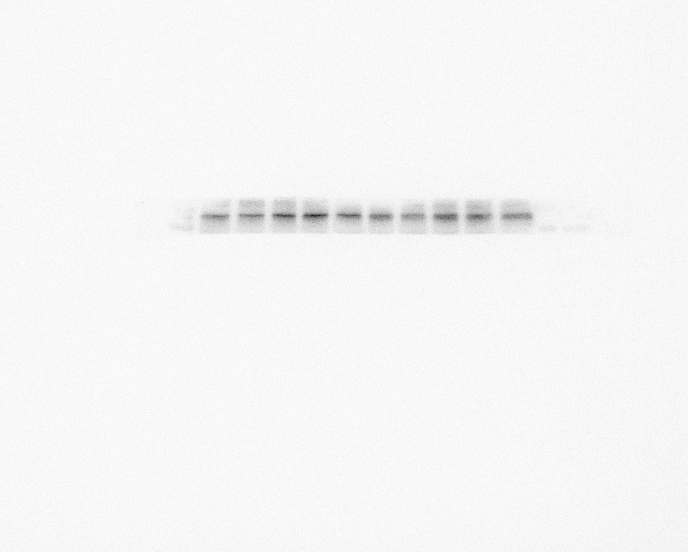

Supplement: Supplementary file 1 [file Data_Sheet_1.ZIP › original WB/SLSP-HEART - ╕▒▒╛/pakt/PAKT-3.tif]
